# The efficacy and safety of qiwei baizhu san in the treatment of type 2 diabetes mellitus: a systematic review and meta-analysis

**DOI:** 10.3389/fphar.2024.1501990

**Published:** 2025-01-07

**Authors:** Quan Zhang, Hongyan Liu, Jiahong Zhang, Yujie Ouyang, Xiaoxu Fu, Chunguang Xie

**Affiliations:** ^1^ Hospital of Chengdu University of Traditional Chinese Medicine, Chengdu, China; ^2^ TCM Regulating Metabolic Diseases Key Laboratory of Sichuan Province, Chengdu, China; ^3^ Department of Endocrinology, Hospital of Chengdu University of Traditional Chinese Medicine, Chengdu, China

**Keywords:** traditional Chinese medicine, qiwei baizhu san, type 2 diabetes mellitus, systematic review, meta-analysis

## Abstract

**Background:**

Type 2 diabetes mellitus (T2DM) is a metabolic disorder characterized by chronic hyperglycemia, mostly resulting from impaired insulin production and diminished glucose metabolism regulation. Qiwei Baizhu San (QWBZS) is a classic formula used in traditional Chinese medicine for the treatment of T2DM. A comprehensive analysis of the efficacy and safety of QWBZS in the treatment of T2DM is essential.

**Methods:**

This study’s protocol was registered with PROSPERO (CRD42024576129). As of August 2024, we searched eight databases to screen and include randomized controlled trials of QWBZS for T2DM. Heterogeneity sources were examined via subgroup analyses, the robustness of the results was determined by sensitivity analyses, publication bias was evaluated using funnel plots and Egger’s test, evidence quality was appraised with GRADEpro, and possible mechanisms of QWBZS for T2DM were categorized and summarized.

**Results:**

This analysis encompassed 14 qualifying trials with a total of 1,169 subjects. The analytical results suggested that QWBZS, when combined with conventional treatment, was more effective than conventional treatment alone in improving FBG, 2hPG, HbA1c, HOMA-IR, TC, TG, LDL-C, and HDL-C. When QWBZS was used alone, it was more effective than conventional therapy in FBG, 2hPG, and HbA1c. And QWBZS could improve the overall effectiveness of clinical treatment in T2DM patients. The impact of QWBZS therapy alone on HOMA-IR and lipid metabolism remained unclear due to the limited number of trials included. Analysis of adverse events suggested that QWBZS was relatively safe.

**Conclusion:**

This study suggested that QWBZS, when combined with conventional treatment, was more effective in improving glucose metabolism, insulin resistance, and lipid metabolism compared to conventional treatment alone in individuals with T2DM. QWBZS alone also contributed to the regulation of blood glucose levels. Meanwhile, QWBZS could improve the overall effective rate of clinical treatment with a relatively high safety profile. Nevertheless, owing to the inferior quality and significant heterogeneity of the existing evidence, additional high-quality studies are requisite to furnish more dependable evidence for the future clinical application of QWBZS.

**Systematic Review Registration:**

https://www.crd.york.ac.uk/PROSPERO/display_record.php?RecordID=576129, identifier [CRD42024576129].

## 1 Introduction

Type 2 Diabetes Mellitus (T2DM) is a chronic metabolic disorder marked by sustained hyperglycemia, impacted by various factors including genetics, environment, and immune response. The pathophysiology of T2DM is characterized by reduced insulin production and decreased insulin sensitivity in peripheral tissues, leading to an imbalance in glucose regulation and ultimately causing abnormal blood glucose levels (Patil et al., 2023). With economic development and lifestyle changes, the global epidemic of T2DM is mostly attributed to sedentary behaviors, high-calorie diets, obesity, and an aging population (Ye et al., 2023). The International Diabetes Federation (IDF) reported in 2021 that approximately 536 million individuals aged 20 to 79 globally have diabetes, with projections indicating an increase to 784 million adults by 2045, and more than 90% of them belong to T2DM (Sun et al., 2022). The rising incidence of T2DM imposes a significant strain on the global health economy. Furthermore, as the condition advances, T2DM results in various complications, including damage to the heart, blood vessels, eyes, kidneys, and nerves (Balooch Hasankhani et al., 2023). T2DM and its complications substantially contribute to the worldwide burden of mortality and morbidity. Consequently, the efficient prevention and management of T2DM and associated complications has emerged as a significant concern in worldwide public health (Li et al., 2020).

Weight management, dietary adjustments, and lifestyle modifications are helpful strategies for the management of T2DM (Association, 2021). Nevertheless, as the disease progresses, patients still need to use some antidiabetic drugs to control blood glucose levels. These drugs may bring about a variety of adverse effects, including hypoglycemia, fluid retention, osteoporosis, and heart failure (Aloke et al., 2022; Shi et al., 2023). In addition, due to the presence of metabolic memory, diabetic complications persist and evolve after the onset of diabetes, even if glycaemic control is restored through pharmacological interventions (Testa et al., 2017; Bheda, 2020). Therefore, there is an urgent need to find safer and more effective treatments.

Traditional Chinese Medicine (TCM), as a unique traditional medicine in China, is the cohesion of the wisdom and the accumulation of valuable experience of the Chinese people over thousands of years. TCM has been extensively embraced worldwide as a complementary and alternative remedy (Hong et al., 2017). With the continuous development of TCM, it has gained more and more recognition worldwide due to its mild and durable therapeutic effects and low toxic side effects (Feng et al., 2020). Chinese medicine has a long history of treating T2DM, and several studies in recent years have indicated that unit Chinese medicine such as Pueraria Mirifica as well as some Chinese medicine metabolites such as ginsenosides and glycyrrhetinic acid can improve clinical symptoms as well as glucose metabolism-related indexes of patients with diabetes and slow down the onset and progression of diabetes-related complications by modulating the immune system, attenuating inflammation responses, and reducing insulin resistance (Bai et al., 2018; Chen et al., 2018; Tan et al., 2022). T2DM belongs to the category of “wasting thirst” in TCM. Qiwei baizhu san (QWBZS), the original name of which is Baizhu San, is from the “Key to Diagnosis and Treatment of Children’s Diseases” by Qian Yi, a famous physician of the Northern Song Dynasty (Qian et al., 2000). Zhang Jiegu, a famous doctor in the Jin Yuan Dynasty, suggested: “People who have the disease of wasting thirst cannot eat and are thirsty, or have abdominal distension, can be treated with qiwei baizhu san.” Qing Dynasty physician Wu Qian listed QWBZS as one of the commonly used formulas for the treatment of wasting thirst in the “Golden Mirror of the Medical Tradition” (Wu et al., 1994). QWBZS is composed of Ginseng Root (Renshen, Panax ginseng C. A. Meyer), modern or replaced by Tangshen (Dangshen, Codonopsis pilosula (Franch.) Nannf.), Largehead Atractylodes (Baizhu, Atractylodes macrocephala Koidz.), Indian bread (Fuling, Poria cocos (Schw.) Wolf.), Liquorice Root (Gancao, Glycyrrhiza glabra L.), Common aucklandia root (Muxiang, Aucklandia lappa Decne.), Wrinkled gianthyssop hearb (Huoxiang, Agastache rugosa (Fisch.etMey.)), Thomson Kudzuvine root (Gegen, Pueraria montana var. thomsonii (Benth.) M. R. Almeida). It has the efficacy of invigorating the spleen and benefiting the qi, harmonizing the stomach, and generating body fluids, and it is a representative formula for the treatment of the insufficiency of qi and yin pattern for the treatment of wasting thirst disorders in TCM. Modern research indicates that QWBZS can enhance clinical symptoms and associated biochemical markers in patients with T2DM (Lin, 2022). In addition, related mechanism studies have also shown that QWBZS can regulate intestinal flora, attenuate the levels of inflammation and oxidative stress, improve disorders of glucose and lipid metabolism, and reduce insulin resistance, thereby lowering blood glucose levels (Liu et al., 2020; Liu et al., 2022). Although a series of clinical studies have indicated that QWBZS is effective in the treatment of T2DM, however, the clinical efficacy and safety of QWBZS in the treatment of T2DM still need to be further validated due to the limited sample size, inconsistent trial design, different efficacy indexes, and unclear methodological quality. Furthermore, there is an absence of a thorough description of evidence-based medicine to evaluate the efficacy and safety of QWBZS in the management of T2DM. Therefore, The objective of this study was to assess the clinical efficacy and safety of QWBZS in treating T2DM by systematically gathering randomized controlled trials (RCTs) of QWBZS administered alone or in conjunction with glucose-lowering medications for meta-analysis, and to categorize and summarize the possible mechanisms of QWBZS in T2DM management, thereby offering a scientific foundation for future research and clinical applications.

## 2 Materials and methods

### 2.1 Research registration

This systematic review and meta-analysis were performed following the Cochrane Handbook for Systematic Reviews of Intervention version 6.3 (2022 update) and the Preferred Reporting Items for Systematic Reviews and Meta-Analysis (PRISMA) 2020 statement (Moher et al., 2009; Page et al., 2021) (Supplementary Material S1). In addition, the protocol of this study was registered in the International Prospective Register of Systematic Reviews (PROSPERO) under the registration number CRD42024576129.

### 2.2 Databases and search strategies

We conducted a systematic search of Chinese and English databases from their inception until August 2024, encompassing PubMed, Cochrane Library, Embase, Web of Science, China Knowledge Network (CNKI), Wanfang Database, China Science and Technology Journal Database (VIP), and China Biomedical Database (CBM). Ongoing studies were also identified through searches of the ClinicalTrials.gov and the China Clinical Trial Registry (CHiCTR) databases. Searches were performed utilizing a combination of subject phrases and textual keywords, including: “Qiwei Baizhu”, “Qiwei Baizhu Powder”, “Qiwei Baizhu San”, “Qiweibaizhu”, “Qiweibaizhu Powder”, “Qiweibaizhu San”, “Type 2 Diabetes Mellitus,” “Diabetes Mellitus, Type 2,” “Type 2 Diabetes,” “Diabetes, Type 2” and “Noninsulin-Dependent Diabetes Mellitus”. Detailed search strategies are provided in Supplementary Material S2.

### 2.3 Inclusion criteria

Type of studies: This study exclusively comprised randomized controlled trials (RCTs). The publication language was restricted to English or Chinese.

Type of participants: This study encompasses persons aged 18 years and older diagnosed with T2DM with or without complications or comorbidities. Demographic factors such as age, race, or gender of the study population were not restricted.

Type of interventions: The intervention consists of the original QWBZS or modified QWBZS, with no restriction on the dosage form (powders, decoctions or granules, etc.), frequency, or dose. The treatment group could use the original QWBZS or the modified QWBZS independently, or alongside conventional therapy. Conventional therapy encompasses diabetes health education, dietary control, exercise intervention, blood glucose monitoring, and hypoglycemic medication. There was no restriction on the type or dosage form of glucose-reducing medication (oral preparation or injection). The control group was administered conventional therapy or a placebo. If the treatment group is combined with conventional therapy at the same time, the conventional therapy program of the treatment group should be the same as that of the control group.

Outcome indicators: Primary outcome indicators included fasting blood glucose (FBG), 2-h postprandial glucose (2hPG), and glycosylated hemoglobin (HbA1c). Secondary outcome indicators included insulin resistance index (homeostasis model assessment of insulin resistance, HOMA-IR), total cholesterol (TC), triglycerides (TG), low-density lipoprotein cholesterol (LDL-C), high-density lipoprotein cholesterol (HDL-C), overall effective rate, and adverse events rate.

### 2.4 Exclusion criteria

Type of studies: This study excluded non-randomized controlled trials, which included case-control studies, case reports, cross-sectional studies, cohort studies, reviews, and experiences of reputable physicians. Studies lacking pertinent data or inaccessible in full text despite author contact were removed. For repeat publications, studies with more complete data were selected.

Type of participants: Individuals with acute metabolic disorders, including diabetic ketoacidosis or infection, significant hepatic impairment (ALT/AST >2.5 times the upper limit of normal), severe renal impairment (Cr > the upper limit of normal or eGFR ≤60 umol/L), critical cardiovascular disease, and those who were pregnant or lactating were excluded.

Type of interventions: Studies that used multiple interventions at the same time or where QWBZS was not the primary intervention, e.g., where TCM formulas other than QWBZS, acupuncture, moxibustion, massage, or acupoint injections were used in combination, will be excluded. In addition, control groups will also be excluded if they use treatment measures other than conventional treatment or placebo.

### 2.5 Study selection and data extraction

In this study, a database was created by importing the search results in the form of a bibliography into EndNote 21 software. The literature was independently screened by two researchers (QZ and HL) under pre-established inclusion and exclusion criteria. First, duplicate literature was removed. Subsequently, abstracts and titles were reviewed to conduct an initial screening. Ultimately, literature that failed to satisfy the criteria was eliminated by reviewing the entire text of the remaining literature. If there were any discrepancies, it was determined after discussion with XF. The first author, year of publication, study design, diagnostic criteria, sample size, gender, average age, disease duration, treatment duration, interventions, outcome metrics, and comorbidities or complications were all extracted and cross-checked by two investigators (QZ and HL) independently using a pre-designed data extraction form. A third researcher (XF) was responsible for assessing and resolving any conflicts.

### 2.6 Risk of bias assessment for included studies

Using the Cochrane Handbook risk of bias assessment instrument for RCTs, the quality of the included studies was independently evaluated by two investigators (QZ and HL). Seven important sources of bias were all evaluated by the tool, including the randomized sequence generation, allocation concealment, blinding of subjects and investigators, blinding of study outcome assessments, completeness of outcome data, selective reporting of study results, and other biases. The risk of bias in each of the seven dimensions was evaluated for each of the included studies. Each aspect was evaluated as “high risk,” “low risk,” or “unclear risk” based on the study’s comprehensiveness and the methodology’s accurate implementation. In the event of a dispute between two researchers (QZ and HL) over the evaluation findings, the ultimate conclusion was deliberated with a third researcher (XF).

### 2.7 Statistical analysis

Review Manager 5.3 and Stata 15.0 were implemented in this investigation to execute meta-analysis. The effect sizes for dichotomous variables were calculated using the relative risk (RR) and 95% confidence interval (CI). For continuous variables, mean difference (MD) and 95% CI were used as effect sizes because the units for the same outcome indicator were consistent in this study. The magnitude of inter-study heterogeneity was assessed by χ^2^ test and I^2^ test. The fixed effect model was used to combine the effects if the heterogeneity was modest, as indicated by an I^2^ < 50% and *p* > 0.1. If I^2^ ≥ 50% or *p* ≤ 0.1 showed that there was considerable heterogeneity. The effects were pooled using a random effect model and subgroup analysis was used to further investigate the source of heterogeneity. A sensitivity analysis was conducted for each outcome indicator to ascertain the stability of the findings. To evaluate publication bias, we plotted funnel plots and conducted Egger’s test for FBG, 2hPG, and overall effective rate outcome metrics that encompassed over ten studies. If *p* > 0.05, it indicates that there is no significant publication bias. If *p* < 0.05, it indicates that there may be publication bias and the asymmetric funnel plots will be further corrected by the clipping and patching method, and the effect sizes of all studies without publication bias after clipping and patching will be compared with the effect sizes of all studies before clipping and patching to determine whether the results are reliable. Lastly, the GRADEpro procedure was implemented in this investigation to evaluate the integrity of the evidence.

### 2.8 Subgroup analysis

We identified factors that may influence treatment outcomes based on the characteristics of the extracted data and performed subgroup analyses based on these factors to explore sources of heterogeneity. Specific subgroup analyses included: Average age (≤50 years old or >50 years old); Disease duration (5–7 years, 7–9 years, >9 years, or not reported); Treatment duration (≤30 days or >30 days); The presence or absence of comorbidities (there are not comorbidities or there are comorbidities).

## 3 Results

### 3.1 Database search results and literature selection

**FIGURE 1:**
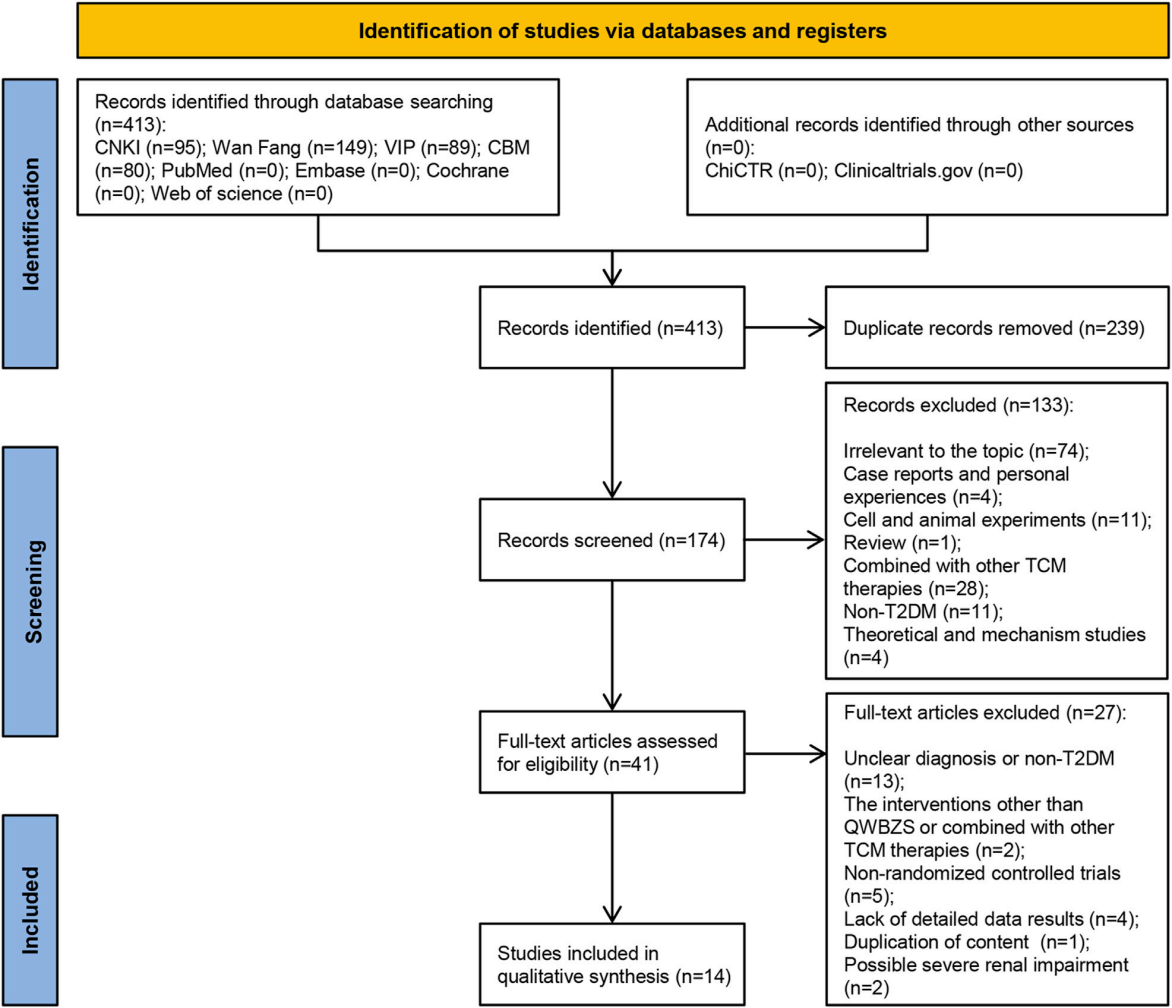
Flow diagram of studies selection process.

Through a search of Chinese and English databases, as well as clinical trial registries, we identified a total of 413 studies in the four Chinese databases, but no relevant research was found in the English databases and clinical trial registries. We eliminated 239 duplicate studies, and among the remaining 174 studies, 133 were excluded based on reading the title and abstract. The full text of the remaining 41 studies was further read and screened against predetermined inclusion and exclusion criteria, resulting in the elimination of 27 studies for following reasons: Unclear diagnosis or non-T2DM (n = 13); The interventions other than QWBZS or combined with other TCM therapies (n = 2); Non-randomized controlled trials (n = 5); Lack of detailed data results (n = 4); Duplication of content (n = 1); Possible severe renal impairment (n = 2). Finally, 14 eligible studies were included. The literature’s initial screening approach is explained in Supplementary Material S3. Supplementary Material S4 contains a list of literature that was excluded after the complete text was read, as well as the rationale behind excluding it. Figure 1 depicts a comprehensive flow diagram of the vetting procedure for eligible studies.

### 3.2 Characteristics of the included studies

This study encompassed 14 randomized controlled trials of QWBZS for the treatment of T2DM in China, which were published between 2014 and 2024. (He and Hu, 2014; Li, 2014; Niu, 2014; Fan, 2019; Liu, 2020; Lin, 2022; Meng et al., 2022; Qian, 2022; Wang et al., 2022; Liang et al., 2023; Wang, 2023; Yang and Zeng, 2023; Liao et al., 2024; Luo, 2024). A total of 1169 T2DM patients were involved, including 586 in the trial group and 583 in the control group. In terms of diagnostic criteria, two studies (He and Hu, 2014; Lin, 2022) used the World Health Organization (WHO) definition, eight studies (Li, 2014; Meng et al., 2022; Qian, 2022; Wang et al., 2022; Liang et al., 2023; Wang, 2023; Yang and Zeng, 2023; Liao et al., 2024) used the Chinese guideline diagnostic criteria, one study (Niu, 2014)used the diagnostic criteria for T2DM in Internal Medicine by Lu Zaiying, and three studies (Fan, 2019; Liu, 2020; Luo, 2024) did not report diagnostic criteria. Intervention durations ranged from a minimum of 10 days (Liao et al., 2024) to a maximum of 3 months (Li, 2014; Meng et al., 2022). 3 studies (Liu, 2020; Liang et al., 2023; Wang, 2023) used the original QWBZS and 11 studies (He and Hu, 2014; Li, 2014; Niu, 2014; Fan, 2019; Lin, 2022; Meng et al., 2022; Qian, 2022; Wang et al., 2022; Yang and Zeng, 2023; Liao et al., 2024; Luo, 2024) used the modified QWBZS, all of which added or subtracted medications based on the patient’s subsidiary syndrome or concomitant symptoms. The composition of the original QWBZS or modified QWBZS is demonstrated in Supplementary Material S5, and none of these studies reported on the quality control or chemical analyses of the QWBZS. 13 studies (He and Hu, 2014; Li, 2014; Niu, 2014; Fan, 2019; Liu, 2020; Meng et al., 2022; Qian, 2022; Wang et al., 2022; Liang et al., 2023; Wang, 2023; Yang and Zeng, 2023; Liao et al., 2024; Luo, 2024) used either the original QWBZS or the modified QWBZS in combination with conventional therapy in the treatment group and conventional therapy in the control group. 1 study (Lin, 2022) used the modified QWBZS alone in the treatment group and conventional therapy in the control group. The characteristics of the 14 randomized controlled trials that were included in the study are shown in Table 1.

**TABLE 1:** Characteristics of the included studies.

Study	Sample size	Gender (M/F)		Age (years)		Course of disease		Duration	Co-intervention	Intervention		Outcome index	Comorbidity
	(T/C)	T	C	T	C	T	C			T	C		
Fan (2019)	30/30	16/14	17/13	54.67	55.13	7.95 ± 2.78	7.92 ± 1.98	8 weeks	Diet and exercise intervention	Modified QWBZS, 1 dose/per day, 150 mL/per, bid + CG	Acarbose tablets, 50 mg, tid; Aspartate insulin,30 IU, bid, the drug dose is adjusted according to blood sugar to maintain the best control of blood sugar	①②③⑨⑩	NR
He and Hu (2014)	30/30	16/14	12/18	56.33 ± 7.96	55.66 ± 7.61	5.02 ± 4.06	5.22 ± 4.07	8 weeks	Diabetes health education + Diet and exercise intervention	Modified QWBZS, 1 dose/per day + CG	Continue to maintain the original hypoglycaemic medication, adjusting the dose to maintain optimal glycaemic control	①②③⑨	NR
Li (2014)	57/57	30/27	32/25	49.17 ± 9.70	48.63 ± 8.36	NR	NR	3 months	Diet and exercise intervention + antihypertensive, lipid adjustment, crown expansion, and complication treatment	Modified QWBZS, 1 dose/per day, 200 mL/per, bid + CG	According to the patient’s blood glucose level, oral hypoglycemic drugs or insulin treatment was given	①②③⑨	Hypertension, coronary heart disease and hyperlipidemia
Liang et al. (2023)	40/40	19/21	18/22	54.23 ± 5.17	53.86 ± 5.24	12.87 ± 2.57	12.41 ± 2.36	1 month	Diet and exercise intervention	Original QWBZS, 1 dose/per day, 200 mL/per, bid + CG	Metformin enteric-coated capsules, 0.2 g, tid	①②③⑨	Obesity
Liao et al. (2024)	40/40	21/19	24/16	34.82 ± 8.11	35.17 ± 8.24	5.09 ± 1.28	4.89 ± 1.17	10 days	Diabetes health education + Diet and exercise intervention	Modified QWBZS, 1 dose/per day, 150 mL/per, bid + CG	Insulin or oral hypoglycemic drugs are given depending on the condition	①②⑨⑩	NR
Lin (2022)	40/40	23/17	22/18	67.55 ± 6.78	67.16 ± 6.52	6.89 ± 2.74	6.72 ± 2.54	30 days	NR	Modified QWBZS, 1 dose/per day, bid	Acarbose tablets, 50mg, tid, the dosage was adjusted according to the blood glucose test results, with the highest dose	①②③⑨	NR
Liu (2020)	40/40	31/9	30/10	44.7 ± 6.1	44.8 ± 6.8	8.2 ± 4.2	8.16 ± 4.1	30 days	NR	Original QWBZS, 1 dose/per day, bid + CG	Metformin tablets, 1.0 g, bid	①②⑨	Asymptomatic hyperuricemia
Luo (2024)	35/35	18/17	21/14	61.54 ± 5.88	64.80 ± 5.94	7.62 ± 1.43	7.54 ± 1.54	2 weeks	NR	Modified QWBZS, 1 dose/per day, 150 mL/per, bid + CG	Metformin tablets, 0.25 g, tid + Glaclazide tablets, 80 mg, bid	①②③⑨	NR
Meng et al. (2022)	48/48	22/26	23/25	54.52 ± 7.08	54.36 ± 7.15	7.15 ± 2.05	7.16 ± 2.03	3 months	Diet, exercise intervention, lifestyle adjustments, and Control of body weight	Modified QWBZS, 1 dose/per day, 100 mL/per, bid + CG	Metformin hydrochloride tablets, 0.25 g, bid or tid. The dose is increased to 1.0–1.5 g/per day. Atorvastatin calcium tablets, 10 mg, qn	①②③⑤⑥⑦⑧⑨	Abnormal lipid metabolism
Niu (2014)	84/83	47/37	45/38	56.4 ± 9.7	56.3 ± 7.2	NR	5.3 ± 3.1	60 days	Diabetes health education + Diet and exercise intervention	Modified QWBZS, 1 dose/per day, bid + CG	Sulfonylurea biguanides and other hypoglycemic drugs or combined insulin therapy according to individual conditions	①②③⑨⑩	NR
Qian (2022)	30/31	14/16	12/19	52.17 ± 11.94	54.03 ± 10.28	8.10 ± 6.03	9.23 ± 5.79	8 weeks	Diabetes health education + Diet and exercise intervention	Modified QWBZS, 1 dose/per day, 200 mL/per, bid + CG	Metformin hydrochloride tablets, 0.5 g, tid	①②③④⑤⑥⑦⑨	NR
Wang et al. (2022)	50/50	30/20	29/21	47.35 ± 6.85	48.02 ± 7.57	5.72 ± 2.73	5.47 ± 2.75	12 weeks	Diet and exercise intervention	Modified QWBZS, 1 dose/per day, 150 mL/per, bid + CG	Metformin hydrochloride extended-release tablets, 0.5 g, bid + acarbose tablets, 50 mg, tid. Adjust the dosage of the medicine according to changes in blood sugar levels	①②③④⑤⑥⑦⑧⑨	NR
Wang (2023)	32/29	15/17	17/12	54.59 ± 7.56	50.86 ± 8.38	10.50 ± 5.81	9.66 ± 4.83	4 weeks	Diabetes health education + Diet and exercise intervention	Original QWBZS (Granules), 1 dose/per day, bid + CG	Oral Western medicine or subcutaneous insulin injection, depending on the individual situation	①④⑨	NR
Yang and Zeng (2023)	30/30	25/5	22/8	52.79 ± 6.78	52.48 ± 6.52	6.71 ± 2.58	6.45 ± 2.71	1 month	Hypoglycemic drugs or insulin injections with the original hypoglycemic regimen	Modified QWBZS, 1 dose/per day, bid + CG	The wound edge and wound were cleaned with an iodine cotton ball, the wound was filled with Rehabilitation new fluid gauze strip, and the wound was wrapped with sterile gauze, bid	①②⑨	Anal fistula

Abbreviations: F, female; M, male; NR, not reported; T, treatment group; C, control group; CG, control group interventions; QWBZS, qiwei baizhu san; Outcome index: ①FBG; ②2hPG; ③HbA1c; ④HOMA-IR; ⑤TC; ⑥TG; ⑦LDL-C; ⑧HDL-C; ⑨Overall effective rate; ⑩Adverse events rate.

### 3.3 Risk of bias assessment

**FIGURE 2:**
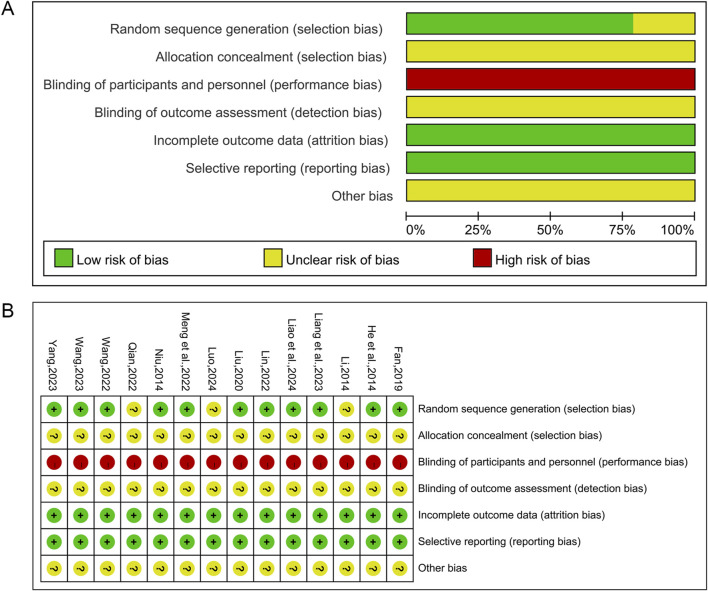
Risk of bias assessment for included studies. **(A)** Risk of bias graph; **(B)** Risk of bias summary.

The Cochrane risk of bias tool was used to assess the risk of bias in this study. Of the 14 included studies, 11 studies (He and Hu, 2014; Niu, 2014; Fan, 2019; Liu, 2020; Lin, 2022; Meng et al., 2022; Wang et al., 2022; Liang et al., 2023; Wang, 2023; Yang and Zeng, 2023; Liao et al., 2024) used randomized number table methods to generate random sequences and were assessed as “low risk”. The remaining three studies (Li, 2014; Qian, 2022; Luo, 2024) claimed to have performed randomization but did not specifically report the method of random sequence generation, and these studies were assessed as “unclear risk”. None of the studies showed blinding of subjects or investigators and were therefore assessed as “high risk”. All studies did not indicate whether the outcome assessors were blinded and were therefore assessed as “unclear risk”. All studies were classified as “low risk” for incomplete data and reporting bias since they reported specific data on relevant indicators and had no or very few patient withdrawals. All studies had unclear allocation concealment and insufficient information to report other biases and were therefore assessed as “unclear risk”. Figure 2 illustrates the findings of the risk of bias assessment conducted on the studies that were included.

### 3.4 Primary outcome indicators

#### 3.4.1 FBG

##### 3.4.1.1 QWBZS combined with conventional treatment vs. conventional treatment

**FIGURE 3:**
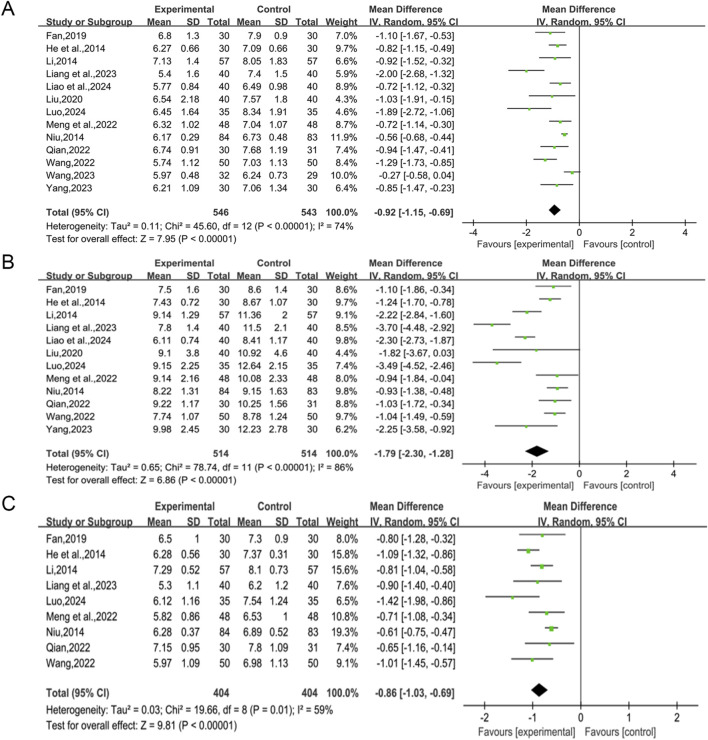
Forest plot for primary outcomes. **(A)** FBG; **(B)** 2hPG; **(C)** HbA1c.

A total of 13 studies (He and Hu, 2014; Li, 2014; Niu, 2014; Fan, 2019; Liu, 2020; Meng et al., 2022; Qian, 2022; Wang et al., 2022; Liang et al., 2023; Wang, 2023; Yang and Zeng, 2023; Liao et al., 2024; Luo, 2024) reported the efficacy of QWBZS combined with conventional therapy *versus* conventional therapy alone on FBG levels in 1,089 patients. A random effect model was selected for statistical analysis according to the heterogeneity test (I^2^ = 74%, *p* < 0.00001, Figure 3A). However, due to significant heterogeneity across studies, we performed subgroup analyses based on different average ages, different disease durations, different intervention durations, and the presence of comorbidities. In the results of the subgroup analyses, there were no differences between different average ages (*p* = 0.73), different disease durations (*p* = 0.74), different intervention durations (*p* = 0.48), and presence of comorbidities (*p* = 0.39). Subgroup analyses showed that heterogeneity within subgroups was not fully reduced, so these factors cannot be considered a major source of heterogeneity at this time (Table 2, Supplementary Material S6.1). Furthermore, sensitivity analyses were implemented to evaluate whether the combined effect sizes could have been substantially affected by a particular study. Sensitivity analyses showed that the combined statistics were similar and the results were robust (Supplementary Material S7A).

**TABLE 2:** Subgroup analysis for FBG, 2hPG, and HbA1c.

	Number of comparisons	Result: MD/RR (95%CI)	*p*−value for overall effect	*p*−value for heterogeneity	I^2^ (%)	*p*−value for subgroup difference
FBG						
All comparisons	13	−0.92 [-1.15,-0.69]	<0.00001	<0.00001	74	
Average age						0.73
≤50 years old	4	−0.98 [-1.26,-0.69]	<0.00001	0.31	16	
>50 years old	9	−0.90 [-1.19,-0.62]	<0.0001	<0.0001	78	
Course of disease						0.74
5–7 years	5	−0.80 [-1.06,-0.54]	<0.00001	0.02	66	
7–9 years	5	−1.04 [-1.38,-0.70]	<0.00001	0.18	36	
>9 years	2	−1.11 [-2.80,0.59]	0.20	<0.00001	95	
Not reported	1	−0.92 [-1.52,-0.32]	0.003	-	-	
Treatment duration						0.48
≤30 days	6	−1.07 [-1.62,-0.52]	0.0001	<0.0001	83	
>30 days	7	−0.86 [-1.08,-0.63]	<0.00001	0.02	61	
Comorbidities						0.39
There are no comorbidities	8	−0.85 [-1.11,-0.58]	<0.00001	0.0001	76	
There are comorbidities	5	−1.07 [-1.51,-0.63]	<0.00001	0.04	61	
2hPG						
All comparisons	12	−1.79 [-2.30,-1.28]	<0.00001	<0.00001	86	
Average age						0.91
≤50 years old	4	−1.84 [-2.60,-1.08]	<0.00001	0.0005	83	
>50 years old	8	−1.78 [-2.50,-1.06]	<0.00001	<0.00001	88	
Course of disease						<0.0001
5–7 years	5	−1.48 [-2.07,-0.89]	<0.0001	<0.0001	84	
7–9 years	5	−1.62 [-2.53,-0.71]	0.0005	0.0009	79	
>9 years	1	−3.70 [-4.48,-2.92]	<0.0001	-	-	
Not reported	1	−2.22 [-2.84, −1.60]	<0.00001	-	-	
Treatment duration						0.0001
≤30 days	5	−2.81 [-3.57,-2.05]	<0.00001	0.01	70	
>30 days	7	−1.22 [-1.54,-0.89]	<0.00001	0.04	54	
Comorbidities						0.23
There are no comorbidities	7	−1.52 [-2.07,-0.97]	<0.00001	<0.00001	86	
There are comorbidities	5	−2.23 [-3.23,-1.23]	<0.0001	0.0003	81	
HbA1c						
All comparisons	9	−0.86 [-1.03,-0.69]	<0.00001	0.01	59	
Average age						0.99
≤50 years old	2	−0.85 [-1.06,-0.65]	<0.00001	0.43	0	
>50 years old	7	−0.86 [-1.08,-0.63]	<0.00001	0.005	67	
Course of disease						0.98
5–7 years	3	−0.88 [-1.25,-0.52]	<0.00001	0.0010	86	
7–9 years	4	−0.86 [-1.17,-0.55]	<0.00001	0.16	41	
>9 years	1	−0.90 [-1.40,-0.40]	0.0005	-	-	
Not reported	1	−0.81 [-1.04,-0.58]	<0.00001	-	-	
Treatment duration						0.22
≤30 days	2	−1.14 [-1.65,-0.64]	<0.0001	0.18	45	
>30 days	7	−0.81 [-0.98,-0.64]	<0.00001	0.03	58	
Comorbidities						0.52
There are no comorbidities	6	−0.90 [-1.17,-0.64]	<0.00001	0.002	74	
There are comorbidities	3	−0.86 [-1.03,-0.69]	<0.00001	0.83	0	

##### 3.4.1.2 QWBZS vs. conventional treatment

One study (Lin, 2022) that included 80 patients with T2DM reported that QWBZS was more effective than conventional therapy in reducing FBG levels after 30 days of treatment, with a statistically significant difference (MD = −1.16 mmol/L, 95% CI [-1.68, −0.64], *p* < 0.001).

#### 3.4.2 2hPG

##### 3.4.2.1 QWBZS combined with conventional treatment vs. conventional treatment

A total of 12 studies (He and Hu, 2014; Li, 2014; Niu, 2014; Fan, 2019; Liu, 2020; Meng et al., 2022; Qian, 2022; Wang et al., 2022; Liang et al., 2023; Yang and Zeng, 2023; Liao et al., 2024; Luo, 2024) reported the efficacy of QWBZS combined with conventional therapy *versus* conventional therapy alone on 2hPG levels in 1,028 patients. A random effect model was selected for statistical analysis according to the heterogeneity test (I^2^ = 86%, *p* < 0.00001). The results of the analysis showed that QWBZS combined with conventional therapy was more effective in improving 2hPG levels in patients with T2DM compared with conventional therapy alone, and the difference was statistically significant (MD = −1.79 mmol/L, 95% CI [-2.30, −1.28], *p* < 0.00001, Figure 3B). Considering the prominent heterogeneity among studies, subgroup analysis was still performed. In the results of the subgroup analysis, there was no difference between different average ages (0.91) and the presence of comorbidities (*p* = 0.23). There were differences between different disease durations (*p* < 0.00001) and different treatment durations (*p* = 0.0001). However, heterogeneity within subgroups was not completely reduced, so these factors cannot currently be considered sources of heterogeneity (Table 2, Supplementary Material S6.2). Sensitivity analyses showed that the combined statistics were similar and the results were robust (Supplementary Material S7B).

##### 3.4.2.2 QWBZS vs. conventional treatment

One study (Lin, 2022) that included 80 patients with T2DM reported that QWBZS was more effective than conventional therapy in reducing 2hPG levels after 30 days of treatment, and the difference was statistically significant (MD = −3.92 mmol/L, 95% CI [-4.91,-2.93], *p* < 0.001).

#### 3.4.3 HbA1c

##### 3.4.3.1 QWBZS combined with conventional treatment vs conventional treatment

A total of nine studies (He and Hu, 2014; Li, 2014; Niu, 2014; Fan, 2019; Meng et al., 2022; Qian, 2022; Wang et al., 2022; Liang et al., 2023; Luo, 2024) including 808 patients reported the efficacy of QWBZS combined with conventional therapy *versus* conventional therapy alone on HbA1c. A random effect model was selected for statistical analysis according to the heterogeneity test (I^2^ = 59%, *p* = 0.01). The results showed that QWBZS combined with conventional therapy was effective in reducing HbA1c levels compared with conventional therapy alone, and the difference was statistically significant (MD = −0.86%,95%CI [-1.03,-0.69], *p* < 0.00001, Figure 3C). The effect of the primary study characteristics on HbA1c was examined using subgroup analysis. No significant differences were observed between subgroups of varying average ages, disease durations, treatment durations, and the presence of comorbidities (*p* = 0.99, 0.98, 0.22, and 0.52, respectively). Heterogeneity within subgroups was not completely reduced; therefore, different ages, disease duration, treatment duration, and presence of comorbidities cannot currently be considered sources of heterogeneity (Table 2, Supplementary Material S6.3). Sensitivity analyses indicated that the results were robust (Supplementary Material S7C).

##### 3.4.3.2 QWBZS vs. conventional treatment

One study (Lin, 2022) that included 80 patients with T2DM demonstrated that QWBZS was more effective than conventional therapy in decreasing HbA1c levels after 30 days of treatment, and the difference was statistically significant (MD = −1.01%,95% CI [-1.33,-0.69], *p* < 0.001).

### 3.5 Secondary outcome indicators

For the secondary outcome measures HOMA-IR, TC, TG, LDL-C, and HDL-C, the included studies only reported a comparison of QWBZS combined with conventional treatment *versus* conventional treatment, and no comparison of QWBZS alone *versus* conventional treatment.

#### 3.5.1 HOMA-IR

**FIGURE 4:**
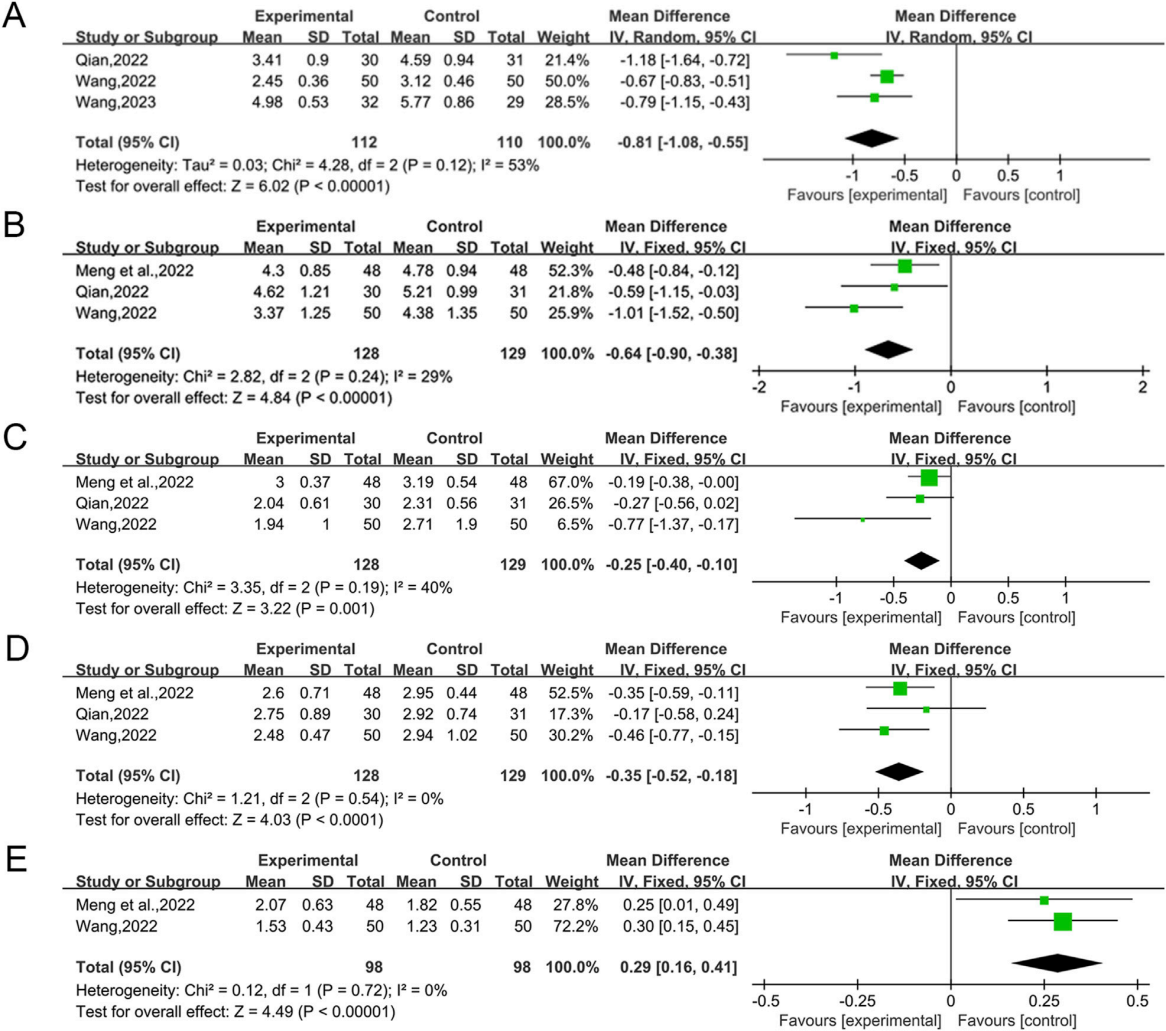
Forest plot for secondary outcomes. **(A)** HOMA-IR; **(B)** TC; **(C)** TG; **(D)** LDL-C; **(E)** HDL-C.

A total of three studies (Qian, 2022; Wang et al., 2022; Wang, 2023) reported the efficacy of QWBZS in combination with conventional therapy *versus* conventional therapy alone for HOMA-IR, involving 222 patients. A random effect model was selected for statistical analysis according to the heterogeneity test (I^2^ = 53%, *p* = 0.12). The analysis showed that QWBZS combined with conventional therapy was statistically more effective than conventional therapy alone in improving HOMA-IR (MD = −0.81, 95% CI [-1.08, −0.55], *p* < 0.00001; Figure 4A). Subgroup analyses were not conducted due to the insufficient number of studies to ascertain the source of heterogeneity through subgroup analysis. Sensitivity analyses indicated that the results were robust (Supplementary Material S7D).

#### 3.5.2 TC

A total of three studies (Meng et al., 2022; Qian, 2022; Wang et al., 2022) reported the efficacy of QWBZS in combination with conventional therapy *versus* conventional therapy alone for TC, involving a total of 257 patients. Statistical analyses were performed using a fixed effect model based on the heterogeneity test (I^2^ = 29%, *p* = 0.24). Overall analysis showed that QWBZS combined with conventional therapy improved TC levels better than conventional therapy alone, and the difference was statistically significant (MD = -0.64 mmol/L,0.95% CI [-0.90,-0.38], *p* < 0.00001; Figure 4B). Sensitivity analyses indicated that the results were robust (Supplementary Material S7E).

#### 3.5.3 TG

A total of three studies (Meng et al., 2022; Qian, 2022; Wang et al., 2022) including 257 patients reported the efficacy of QWBZS combined with conventional therapy *versus* conventional therapy alone for TG. Statistical analyses were performed using a fixed effect model based on the heterogeneity test (I^2^ = 40%, *p* = 0.19). The combined use of QWBZS and conventional therapy resulted in a statistically significant reduction in TC levels when compared to conventional therapy alone, as indicated by the pooled results (MD = −0.25 mmol/L, 95% CI [-0.40, −0.10], *p* = 0.001; Figure 4C). Sensitivity analyses indicated that the results were robust (Supplementary Material S7F).

#### 3.5.4 LDL-C

A total of three studies (Meng et al., 2022; Qian, 2022; Wang et al., 2022) reported the efficacy of QWBZS in combination with conventional therapy *versus* conventional therapy alone on LDL-C, involving 257 patients. Statistical analyses were performed using a fixed effect model based on the heterogeneity test (I^2^ = 0%, *p* = 0.54). The combined use of QWBZS and conventional therapy resulted in a statistically significant improvement in LDL-C compared to conventional therapy alone, as indicated by the pooled results (MD = −0.35 mmol/L, 95% CI [-0.52, −0.18], *p* < 0.0001; Figure 4D). Sensitivity analyses indicated that the results were robust (Supplementary Material S7G).

#### 3.5.5 HDL-C

A total of two studies (Meng et al., 2022; Wang et al., 2022) reported the efficacy of QWBZS combined with conventional therapy *versus* conventional therapy alone on HDL-C, involving a total of 196 patients. Statistical analyses were performed using a fixed effect model based on the heterogeneity test (I^2^ = 0%, *p* = 0.72). The results indicated that the combination of conventional therapy and QWBZS resulted in a statistically significant enhancement in HDL-C levels in T2DM that was superior to conventional therapy alone (MD = 0.29 mmol/L, 95% CI [0.16,0.41], *p* < 0.00001; Figure 4E). Sensitivity analyses indicated that the results were robust (Supplementary Material S7H).

#### 3.5.6 Overall effective rate

**FIGURE 5:**
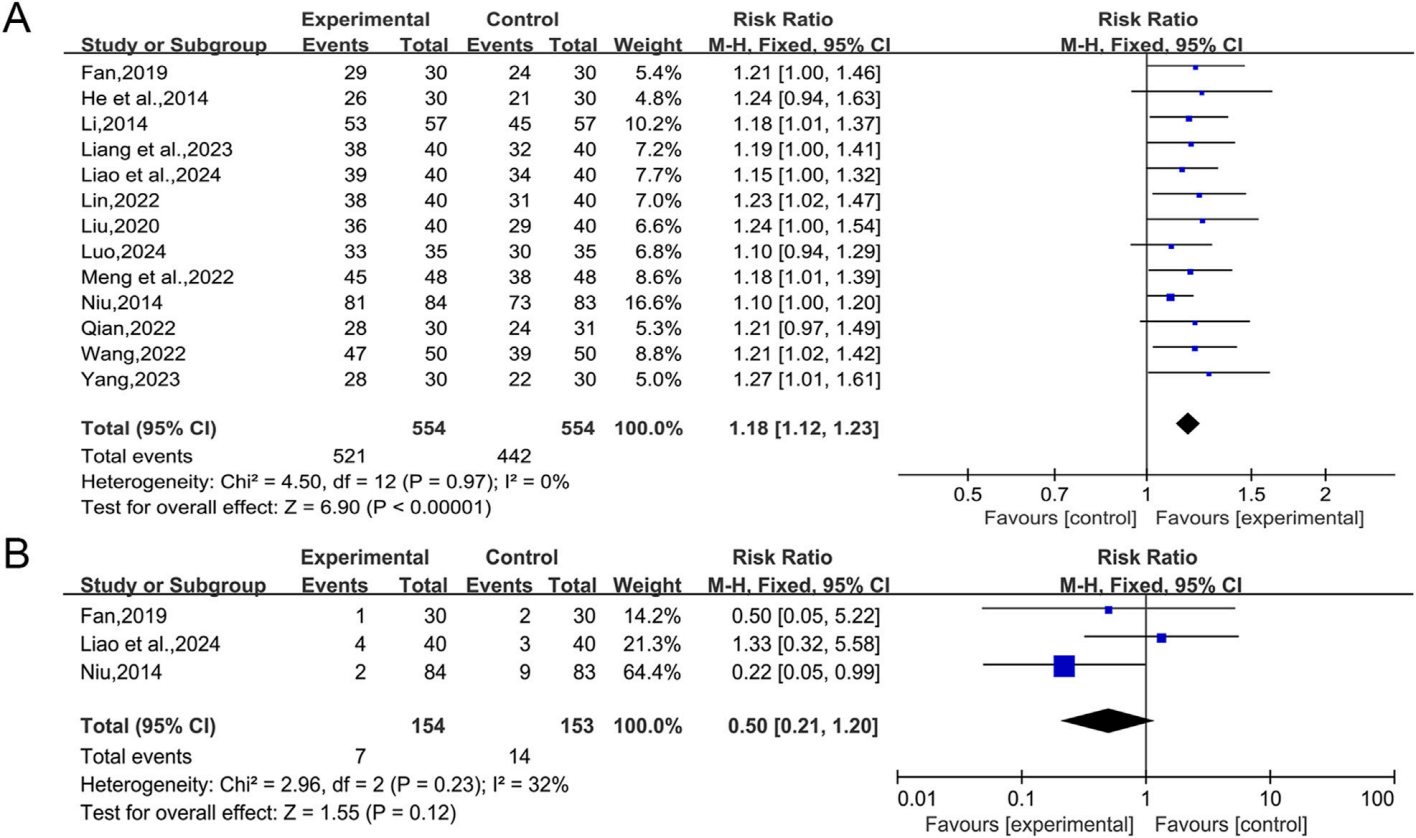
Forest plot for secondary outcomes. **(A)** Overall effective rate; **(B)** Adverse events rate.

The 14 studies included (He and Hu, 2014; Li, 2014; Niu, 2014; Fan, 2019; Liu, 2020; Lin, 2022; Meng et al., 2022; Qian, 2022; Wang et al., 2022; Liang et al., 2023; Wang, 2023; Yang and Zeng, 2023; Liao et al., 2024; Luo, 2024) all reported overall effective rate, involving a total of 1,169 patients. The heterogeneity test (I^2^ = 41%, *p* = 0.05) indicated a high degree of heterogeneity, and a large effect of Wang (2023) on the heterogeneity was found by Labbe plot analysis (Supplementary Material S8). The heterogeneity test was performed again after removing this study, and the results showed a significant reduction in heterogeneity between the remaining 13 studies (I^2^ = 0%, *p* = 0.97), and a fixed effect model was selected for statistical analysis. The results showed that QWBZS combined with conventional therapy enhanced the overall effective rate of T2DM better than conventional therapy alone, and the difference was statistically significant (RR = 1.18, 95% CI [1.12,1.23], *p* < 0.00001; Figure 5A). Sensitivity analyses indicated that the results were robust (Supplementary Material S7I).

#### 3.5.7 Adverse events rate

Among the 14 studies included, adverse events were mentioned in seven studies. Four of these studies (Meng et al., 2022; Qian, 2022; Wang, 2023; Luo, 2024) showed that patients did not experience adverse events while taking the drug, while the other three studies (Niu, 2014; Fan, 2019; Liao et al., 2024) reported adverse events. Fan (2019) stated that one patient (3.33%) in the combination treatment group suffered loose and frequent feces, but this was due to the patient’s taking the drug cold on an empty stomach, and the symptoms improved after the patient was instructed to take the drug warmly after meals. Two patients (6.67%) in the conventional treatment group developed mild dry mouth, dry skin, itchy skin and gastrointestinal discomfort after 1 week of taking the drug, but they were tolerated without treatment. Liao et al. (2024) identified 4 cases (10.00%) of adverse events in the combination treatment group, including 2 cases of nausea and vomiting, 1 case of dizziness, and 1 case of abdominal distension. 3 cases (7.50%) of adverse events were reported in the conventional treatment group, including 1 case of diarrhea, 1 case of nausea, and 1 case of dizziness. The difference in the occurrence of adverse responses between the two groups was not statistically significant (*p* > 0.05). Niu (2014) reported 2 cases (2.38%) of adverse events in the combined treatment group, including 1 case of gastrointestinal symptoms such as nausea and abdominal distension, and 1 case of transient transaminase elevation, and the later treatment was not affected by all the adverse events. 9 cases (10.84%) of adverse events occurred in the conventional treatment group, including 5 cases of gastrointestinal symptoms such as abdominal distension, and loss of appetite, 2 cases of transient transaminase elevation, 1 case of skin itching, and 1 case of transient headache. A statistically significant difference in the incidence of adverse events between the two groups was observed (*p* < 0.05). A fixed effect model was chosen for statistical analysis according to the heterogeneity test (I^2^ = 32%, *p* = 0.23). The adverse events rate between the conventional therapy group and the combination therapy group did not exhibit any significant difference in the aggregated results (RR = 0.50, 95% CI [0.21, 1.20], *p* = 0.12; Figure 5B). Sensitivity analyses indicated that the results were robust (Supplementary Material S7J). None of the three studies that reported adverse events stated whether there were specific adverse reactions to QWBZS, and no adverse events were mentioned in any of the other seven studies. The results suggest that QWBZS is relatively safe. However, due to the limited number of included studies, most of which had small sample sizes, there may be unrecognized adverse events, and more high-quality studies are needed in the future to comprehensively and accurately assess its safety and the presence of specific adverse reactions.

### 3.6 Publication bias

**FIGURE 6:**
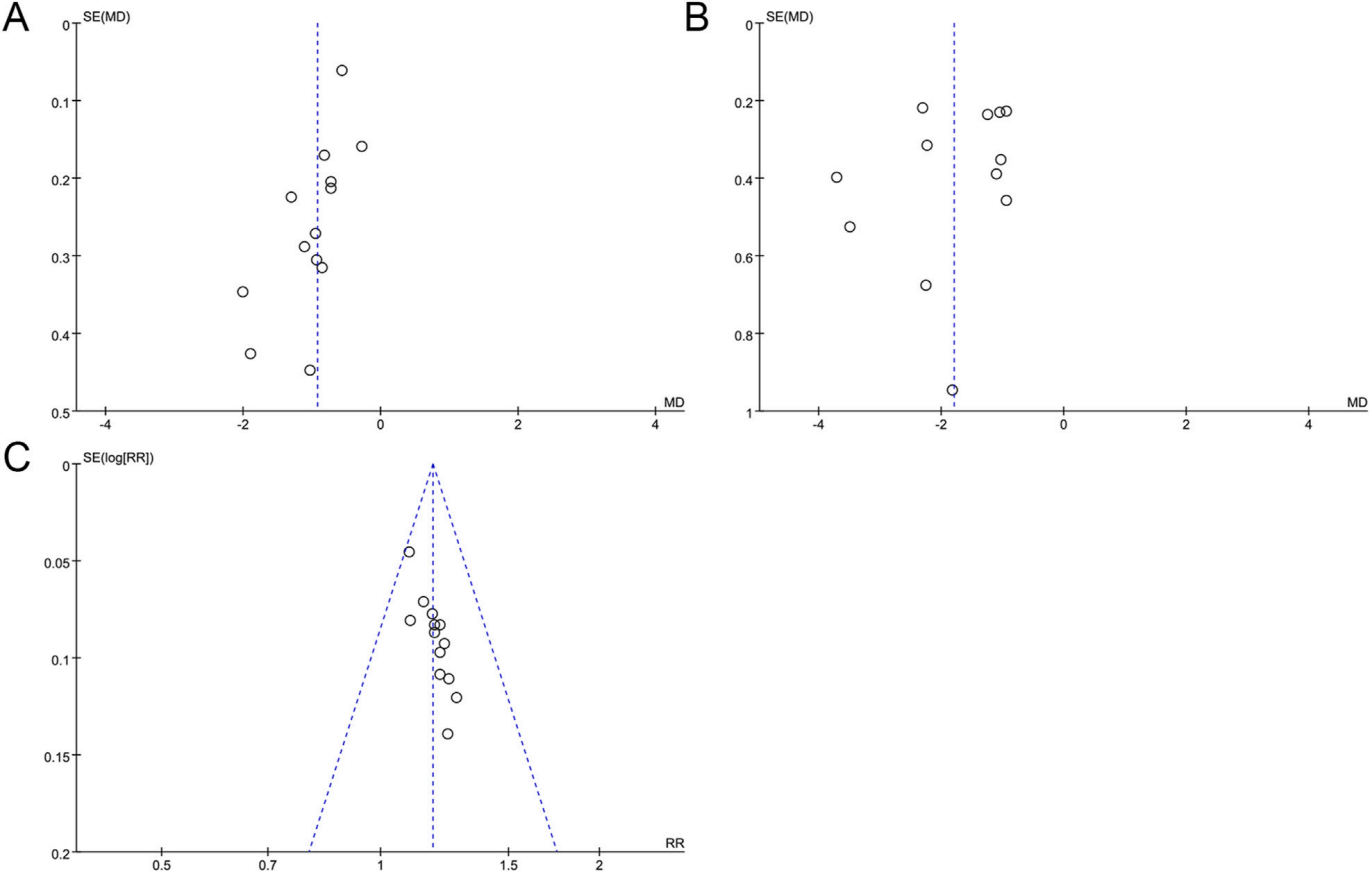
Funnel plots for assessing publication bias. **(A)** FBG; **(B)** 2hPG; **(C)** Overall effective rate.

For outcomes that contained 10 or more included studies, we performed a publication bias assessment. Publication bias needed to be assessed by drawing funnel plots and Egger’s test. The funnel plot of FBG showed an asymmetrical left and right distribution (Figure 6A), and Egger’s test result (*p* = 0.006) indicated a statistical difference (Supplementary Material S9A), which suggests that there may be a publication bias in the studies of FBG. The asymmetric funnel plot was further corrected using the clipping and patching method, which after two iterations resulted in a final virtualized result of 0 literature with no additional studies added (Supplementary Material S10A). The research using the clipping and patching method revealed no asymmetry in the funnel plot indicative of publication bias. The asymmetry of the funnel plot may result from sources beyond publication bias. The results of the clipping and patching analysis were consistent with the preliminary analysis, which indicated that the results were relatively robust. There was a slight asymmetry in the funnel plot for 2hPG (Figure 6B), but Egger’s test result (*p* = 0.329) indicated that there was no significant publication bias in the studies of 2hPG (Supplementary Material S9B). For the overall effective rate, a funnel plot was plotted (Figure 6C) and Egger’s test was performed (*p* < 0.05), which indicated that there was a publication bias in the studies of the overall effective rate (Supplementary Material S9C). The correction was performed using the clipping and patching method, and after five iterations, the results of six papers were finally virtualized, while a total of 19 papers after clipping and patching (Supplementary Material S10B) were free of publication bias, and the effect size of the 19 papers combined was 1.14. Comparing the results before clipping and patching (RR = 1.18) with those after clipping and patching, the results showed minimal changes. This suggests that the results of the existing analyses are stable and that if new findings or new reports are made in the future, they will not significantly affect the results of the analyses.

### 3.7 Assessment of the quality of evidence

To evaluate the quality of the evidence, we used GRADEpro. Limitations, inconsistency, indirectness, precision, and publication bias were taken into consideration while evaluating studies. The results showed that the quality of evidence was low to very low for all outcomes (Supplementary Material S11). The primary causes of the declining quality of evidence were inconsistent study designs, imprecise outcomes, and a lack of blinding. Therefore, the clinical efficacy and safety of QWBZS for T2DM need to be further assessed in the future through large-scale, high-quality, comprehensive, and standardized randomized controlled trials.

## 4 Discussion

### 4.1 Main results of this research

Systematic review and meta-analysis have emerged as critical research methodologies for evaluating clinical evidence as a result of the widespread application and ongoing advancement of evidence-based medicine. Such studies have a high level of medical evidence and can provide a solid foundation for clinical decision-making. The latest systematic evidence for the clinical application of QWBZS was provided by this study, which for the first time analyzed the efficacy and safety of QWBZS in the treatment of T2DM. We conducted an extensive examination of both Chinese and English databases, ultimately incorporating 14 papers following a meticulous series of screenings. A comprehensive meta-analysis was performed focusing on glucose metabolism, insulin resistance index, lipid metabolism, overall effective rate, and adverse events rate. To elucidate or mitigate the observed heterogeneity, we undertook comprehensive subgroup analyses predicated on the specific characteristics of participants (e.g., average age, duration of disease, treatment duration, and status of comorbidities). Furthermore, we evaluated the robustness of the findings via sensitivity analyses, examined publication bias employing funnel plots and Egger’s test, and appraised the quality of evidence utilizing GRADEpro. Analysis of the data suggested that QWBZS in combination with conventional therapy was more effective in improving FBG, 2hPG, HbA1c, HOMA-IR, TC, TG, LDL-C, and HDL-C than conventional therapy alone. When QWBZS was used alone, its efficacy in improving FBG, 2hPG, and HbA1c was superior to conventional therapy. In addition, QWBZS could improve the overall effective rate of clinical treatment. In evaluating the safety profile of QWBZS, it was noted that the majority of adverse events went unreported and no serious adverse events were identified, suggesting that QWBZS was relatively safe. Funnel plots and Egger’s test indicated the presence of publication bias for certain indicators. However, the results remained largely unchanged following the correction using the clipping and patching method. There was significant heterogeneity and poor methodological quality in the literature included in the study, leading to a reduction in the quality of the evidence assessed by GRADEpro, but sensitivity analyses indicated that the results remained robust.

Some of the outcome measures were highly heterogeneous. In most of the subgroup analyses, we found that heterogeneity was not completely reduced and that QWBZS had comparable efficacy in T2DM populations of different ages, disease durations, treatment durations, and the presence of comorbidities. We speculate that, firstly, the heterogeneity could be associated with the varying methodological quality of the studies included. There may have been differences in randomization methods, allocation concealment, and implementation of blinding across studies, especially in the implementation of blinding which was not specifically described. These differences in study methodology may have allowed variation in results between studies, which in turn led to heterogeneity. Secondly, the specificity of QWBZS for the treatment of T2DM should also be considered. T2DM is classified under the “wasting thirst” category in TCM, with a prevalent underlying pathogenesis characterized by a “qi and yin deficiency pattern.” The Yellow Emperor’s Classic of Internal Medicine pointed out that wasting thirst and the spleen have a close relationship, spleen qi deficiency and dysfunctional transport and transformation, dampness stagnation, fluids can not be normally transported, so the human body yin deficiency, the viscera lose nourishment and moistening, resulting in wasting thirst. Every classic formula in TCM has a defined effect, and so does QWBZS. QWBZS can strengthen the spleen and remove dampness, benefit qi, nourish the stomach, generate fluids, and quench thirst, and is a classic formula in TCM for the treatment of deficiency of qi and yin pattern in wasting thirst. It can be considered for patients with T2DM who are identified in TCM as having insufficiency of qi and yin pattern or accompanied by spleen deficiency and dampness stagnation pattern, regardless of age, duration of disease, and the presence or absence of comorbidities. Furthermore, the differentiation of syndromes and treatment, along with holistic concepts, are the characteristics of TCM in addressing illnesses. In the clinical setting, the discrepancies in a patient’s constitution, accompanying syndromes, and associated symptoms are considerable. Additionally, the physiological and pathological traits of the human body are different across different seasons and geographical locations, leading to distinct approaches in medication usage. In clinical practice, practitioners of TCM will choose various botanical drug remedies for addition and subtraction based on the foundational formula of QWBZS, taking into account the specific circumstances of each patient along with seasonal and geographical variations. This approach aims to specifically address the individual condition of each patient to enhance therapeutic outcomes. Thereby, it also produces different botanical drug formulas and dosages for each patient. In conclusion, QWBZS has good efficacy in T2DM patients of different ages, disease durations, and the presence or absence of comorbidities who are characterized by qi and yin deficiency pattern in TCM. The clinical application of QWBZS in treating T2DM focuses on assessing if the patient exhibits traits of qi and yin deficiency pattern in TCM, including symptoms like thirst and excessive drinking, diminished mental energy, lethargy, fatigue, or possibly reduced appetite and loose stools.

### 4.2 Possible mechanisms of QWBZS for T2DM treatment

The intricate mechanisms underlying T2DM and the limited comprehension of the protective effects of QWBZS in this pathological context present significant obstacles to the investigation and advancement of the clinical use of QWBZS. To establish more complete evidence, we have summarised the possible mechanisms of QWBZS in the treatment of T2DM based on current relevant studies as follows.

#### 4.2.1 Regulating intestinal flora

The gut flora is considered a “microbial organ”. A disequilibrium in the balance between its microbial communities may trigger or exacerbate diabetes and its associated complications, such as obesity, metabolic syndrome, and inflammatory bowel disease (Alexander et al., 2017; Aarnoutse et al., 2019). A high-sugar and high-fat diet disrupts the ecological balance of the intestinal flora, leading to an imbalance of the intestinal flora, which in turn triggers insulin resistance and ultimately leads to the development of T2DM (Perry et al., 2016). It is unclear how changes in gut flora directly induce T2DM, but may involve a variety of factors such as low-grade chronic inflammation, production of short-chain fatty acids, and activation of nuclear receptor signaling pathways (Everard and Cani, 2013; Li et al., 2013; Perry et al., 2016).

Several experimental studies have found that QWBZS has the potential to regulate the intestinal flora, can inhibit or kill harmful bacteria in the intestinal tract, and at the same time helps to promote the proliferation of beneficial bacteria, thereby increasing the diversity of intestinal flora (Guo et al., 2015; Pu et al., 2017). QWBZS can promote the growth of beneficial bacteria such as lactobacilli, yeast, bifidobacteria, etc., by regulating the distribution of nutrients in the intestine, acting on the cell wall, cell membrane, or metabolic pathway of harmful bacteria, inhibiting the growth of pathogenic bacteria such as *Staphylococcus aureus*, *E. coli*, etc., and regulate the balance of intestinal microorganisms to restore the normal micro-ecological environment of the intestine. The restoration of intestinal microecological balance helps to enhance intestinal barrier function and reduce the release of intestinal endotoxins and inflammatory factors, thus enhancing the sensitivity of peripheral tissues to insulin and improving insulin resistance (Jiang et al., 2013; Guo et al., 2018). Therefore, improving the imbalance of intestinal flora may be the key to treating T2DM with QWBZS. According to TCM, the spleen can transform water and food into fine and microscopic essence, and the function of the spleen in transport transformation aligns with the metabolic and immune roles of intestinal flora in the body to some degree. Clinically, QWBZS is commonly used to regulate the spleen and stomach functions of T2DM patients (Wang et al., 2022), and is a commonly used formula for the treatment of T2DM and intestinal flora dysbiosis (Liao, 2022).

#### 4.2.2 Inhibiting oxidative stress

Oxidative stress is acknowledged as a possible mechanism contributing to the failure of pancreatic β-cells in T2DM. A persistent hyperglycemic state leads to excessive production of reactive oxygen species (ROS) by increasing mitochondrial oxygen consumption, disrupting mitochondrial function, and activating nicotinamide adenine dinucleotide phosphate oxidase. Excess ROS induces β-cell dysfunction and insulin resistance, which ultimately leads to the progression of T2DM and its complications (Zhang et al., 2020; Bhatti et al., 2022).

QWBZS was found to be effective in improving anti-oxidative stress-related indexes in rats with T2DM pattern of qi and yin deficiency. QWBZS was able to increase the activities of superoxide dismutase (SOD) and glutathione peroxidase (GSH-Px) in T2DM rats with a deficiency of qi and yin. The increase in the activities of these antioxidant enzymes can help to inhibit oxidative stress and reduce the damage of free radicals to the tissues of the body, which in turn can improve the metabolic disorders due to oxidative stress in the diabetic state and reduce the fasting blood glucose level of the T2DM model rats (Yang et al., 2019).

#### 4.2.3 Reducing the inflammatory response

T2DM is a metabolic disease marked by a low-level inflammatory state, and this inflammatory response is manifested by increased release of inflammatory factors and infiltration of inflammatory cells (Burcelin et al., 2012). Tumor necrosis factor-α (TNF-α) serves as an immunomodulatory factor in the pro-inflammatory response and is significantly linked to the onset of insulin resistance (Chait and den Hartigh, 2020). Nuclear factor-κB (NF-κB) is crucial in the context of T2DM, as obesity triggers the activation of this transcription factor. This activation leads to the upregulation of various inflammatory and tumor-promoting cytokines, such as IL-6, IL-1α, and TNF-α, thereby heightening the risk of developing T2DM (Jurjus et al., 2016). Lipopolysaccharide (LPS), originating from the outer membrane of Gram-negative bacteria, serves as a significant inflammatory trigger and is acknowledged as a catalyst for low-grade inflammation. LPS is a key bridge connecting intestinal flora, endotoxemia, and chronic inflammation, and an important contributor to the development of diabetes (Burcelin et al., 2012). Circulating LPS associates with CD14-Toll-like receptor, facilitated by CD14, thereby activating TLR-4, which subsequently activates the transcription factor NF-κB, initiating the expression of inflammatory mediators (e.g., TNF-α, IL-1, IL-6, IL-8, etc.), provoking a cascade of nonspecific inflammatory responses and resulting in the onset of insulin resistance (Mohammad and Thiemermann, 2020).

QWBZS was found to reduce serum lipids and inflammatory factors in T2DM mice, thereby exhibiting therapeutic effects by significantly lowering fasting blood glucose (Liu et al., 2024). A well-functioning intestinal barrier facilitates the effective transcellular transport of nutrients, while simultaneously blocking the entry of harmful bacterial products into the bloodstream through this barrier. QWBZS has the potential to enhance the repair of the mechanical barrier of the intestinal mucosa by increasing the expression of intestinal tight junction proteins in diabetic rats. This action helps maintain the integrity of the tight junctions, lowers serum LPS levels, inhibits the TLR4/NF-κB pathway, and decreases the release of inflammatory factors such as IL-6 and TNF-α. Consequently, this leads to a reduction in the body’s inflammatory response, which may improve insulin resistance and lower blood glucose levels (Wang et al., 2023). Another study based on T2DM patients also found that QWBZS granules significantly reduced the levels of inflammatory factors, improved insulin resistance, lowered blood glucose, and improved the clinical symptoms and Chinese medicine symptom scores of patients with spleen deficiency pattern T2DM by inhibiting the TLR4/NF-κB signaling pathway (Wang, 2023).

#### 4.2.4 Improving insulin resistance

In T2DM, the liver, muscle, and adipose tissue serve as the primary organs exhibiting insulin resistance. A significant pathological mechanism contributing to this resistance is the disorder of the AMPK signaling pathway (Entezari et al., 2022). Activation of the AMPK signaling pathway can decrease PEPCK and G6pase expression, limit hepatic gluconeogenesis, enhance insulin signaling, elevate hepatic and muscle insulin sensitivity, and facilitate glucose uptake and utilization. AMPK activation has been shown to considerably improve blood glucose levels in T2DM patients (Weis et al., 2017; Abulizi et al., 2020).

It was found that QWBZS can increase the expression of AMPK, PI3K, and Akt, and its mechanism of action may be to upregulate AMPK expression by affecting upstream regulators and regulating intracellular redox status, promote the activity of PI3K upstream signaling molecules to enhance its expression, and increase the expression of Akt through the activation and inhibition of the negative regulator Protein Tyrosine Phosphatase Gene (PTEN) by PI3K. AMPK activation regulates glucose uptake and promotes fatty acid entry into mitochondria for oxidative metabolism, reducing the inhibitory effect of fatty acids on the insulin signaling pathway. The PI3K-Akt signaling pathway promotes glucose transporter protein translocation and glycogen synthesis, which increases glucose uptake and utilization by cells. Meanwhile, Akt can also inhibit hepatic glucose production and further improve the insulin resistance status by inhibiting the activity of transcription factors such as FoxO1 and reducing the expression of gluconeogenesis-related genes. This suggests that the mechanism by which QWBZS ameliorates insulin resistance may be related to the activation of the AMPK/PI3K/Akt signaling pathway and the enhancement of cellular sensitivity to insulin (Lv, 2022). Another study also found that QWBZS alleviated insulin resistance in diabetic encephalopathy (DE) rats by a mechanism that may be related to the repair of hippocampal neuronal damage in DE rats by activating the PI3K/Akt/GSK-3β signaling pathway (Gao et al., 2024). Moreover, increased fatty acid levels impede insulin-mediated glucose transport activity, hence causing insulin resistance. Meanwhile, insulin resistance also causes lipid metabolism disorders (Wei et al., 2022). QWBZS was able to improve insulin resistance, regulate insulin secretion, increase hepatic glycogen synthesis, regulate lipid metabolism disorders, and reduce the levels of blood glucose, TC, and TG in T2DM mice (Wang et al., 2020).

#### 4.2.5 Promoting the secretion of GLP-1

Glucagon-like peptide-1 (GLP-1) is an endogenous short-molecule peptide hormone typically synthesized by intestinal L-cells upon food intake. GLP-1 performs several functions, including the stimulation of insulin production, suppression of glucagon secretion, delay of stomach emptying, and diminution of food consumption. The release of GLP-1 is exclusively reliant on glucose, hence reducing the risk of hypoglycemia (EISSELE et al., 1992; Wong et al., 2014). Patients with T2DM have a diminished GLP-1 response to oral glucose and possess lower baseline GLP-1 levels compared to non-diabetic obese individuals (Mannucci et al., 2000).

The QWBZS, represented by the method of strengthening the spleen and qi and resolving dampness, can effectively improve the clinical symptoms of the patients, reduce the blood glucose level, and regulate the glucagon-like peptide-1 (GLP-1) level. Its regulation of GLP-1 in T2DM patients with spleen deficiency and entrapped dampness may be realized by improving intestinal function, increasing nutrient absorption, and stimulating intestinal L cells to secrete GLP-1. QWBZS can promote normal intestinal peristalsis and digestion and absorption by enhancing the transport and digestion ability of the spleen and stomach, thus optimizing the intestinal environment, maintaining the integrity of the intestinal mucosa, and creating a suitable environment for the survival and secretion of intestinal L-cells. After intestinal function is improved, the absorption of nutrients is increased, which provides a material basis for the synthesis and secretion of GLP-1 by intestinal L cells. In addition, adequate nutritional supply can stimulate the activity of intestinal L-cells and ensure their normal synthesis and secretion of GLP-1, which can promote the proliferation of pancreatic islet β-cells, enhance the level of insulin secretion, improve the function of β-cells, and ultimately realize the effective regulation of blood glucose level (He and Hu, 2014; Fan, 2019).

In conclusion, QWBZS serves a distinctive function in the prevention and treatment of T2DM due to its multi-metabolites, multi-pathway, and multi-target benefits. The mechanism of QWBZS in the prevention and treatment of T2DM may include the regulation of intestinal flora, suppression of oxidative stress, decrease of inflammatory response, improvement of insulin resistance, and stimulation of GLP-1 secretion. Nonetheless, the chemical makeup of Chinese medicine formulations is intricate, and the mechanism of therapeutic action of QWBZS on T2DM remains incompletely understood, and more definitive therapeutic mechanisms need to be further investigated in the future.

### 4.3 Strengths and limitations of this study

This study is the inaugural investigation evaluating the efficacy and safety of QWBZS in treating T2DM via systematic review and meta-analysis, while also summarizing the possible mechanisms of QWBZS in T2DM management, thereby offering a reference basis for future research and clinical applications. A thorough and methodical search of recognized databases and clinical registry centers was conducted using a wide range of search terms. Throughout this study, we adhered rigorously to the methods and criteria of the systematic review and used GRADEpro to assess the quality of evidence for the findings. The results were interpreted with care to ensure accuracy and avoid misleading conclusions. The study’s findings suggested that the combination of QWBZS with conventional treatment improved glucose-lipid metabolism and insulin resistance related to T2DM compared to conventional therapy alone, whereas QWBZS alone improved FBG, 2hPG, and HbA1c levels in T2DM patients. In addition, QWBZS enhanced the overall effective rate of clinical treatment. In terms of safety, no significant adverse effects were noted, suggesting that QWBZS was comparatively safe. Consequently, this study offers novel insights and pathways for therapeutic therapy.

Nonetheless, this study does have many limitations. First, the analysis indicated considerable heterogeneity among the studies that were incorporated. Despite conducting subgroup analyses to investigate the origin of the significant heterogeneity, the findings from these analyses suggested that the source of the heterogeneity continued to be unclear. The heterogeneity may also be associated with the quality of botanical drugs, the techniques employed in drug decoction, and the specific traits of syndrome differentiation and treatment in TCM. The subgroup analyses established the thresholds for age, disease duration, and treatment duration primarily on the pertinent data derived from the studies included, and thus more clinical evidence is needed. Second, the number of included studies was small and of low methodological quality. The method of randomization was unclear in a subset of studies, and all included studies lacked clear reporting of blinded allocation and allocation concealment, which could lead to serious bias. Therefore, our conclusions should be regarded as preliminary and subject to uncertainty and need to be further validated by designing more rigorous randomized controlled trials. Third, the literature included in this study lacked placebo control, so the net efficacy of QWBZS in T2DM could not be determined. Fourth, all research included was derived from Chinese literature, which may have ethnic and geographic limitations, and it remains to be elucidated whether QWBZS can be applied to other races. Fifth, none of the included studies were registered or had access to study protocols. Positive results were more likely to be published in China, and there was some publication bias. Finally, most of the literature included in this study did not report whether there were any adverse events, and those that did reported only gastrointestinal, skin-related symptoms, patient self-perception, and hepatic function, and did not cover more relevant metrics for evaluating its safety. Consequently, further high-quality trials and more extensive indicators are required to further validate the effectiveness and safety of QWBZS in T2DM.

### 4.4 Suggestions for future clinical research in TCM

Based on the above findings and limitations of this study, the following recommendations are provided for future clinical research in TCM: First, the criteria for TCM evidence-based treatment should be further improved to clarify the diagnostic points of various types of evidence and the quantitative diagnostic criteria for different TCM types in terms of tongue, pulse, and symptoms, so as to provide a basis for the standardization of clinical research in TCM. Second, the quality standards and specifications of Chinese herbal medicines and Chinese medicine exempted from decoction granules should be standardized. Strict quality standards for Chinese herbal medicines should be formulated, and a traceability system for Chinese herbal medicines should be established to minimize the impact of differences in the quality of Chinese herbal medicines on the accuracy of research results. Great efforts should be made to develop decoction-free granule preparations of Chinese medicines and set strict standards for granule preparations so that different studies will have consistency in terms of Chinese medicine preparations. Third, the quality of clinical research design has been improved. Study design strictly follows internationally recognized clinical research norms, such as the Consolidated Standards of Reporting Trials (CONSORT) statement. Ensure the scientific design and accurate implementation of randomization methods, blind allocation, and allocation concealment. Adopt computer-generated randomized sequences and an independent third party to be responsible for allocation concealment to ensure the randomness and true objectivity of the research process. Conduct multi-center and large-sample clinical studies to improve the reliability and generalizability of the study results. Fourth, placebo control should be set up as far as possible. For decoction-free granules of traditional Chinese medicine, placebos that mimic the appearance, odor, and taste of the drug without affecting the therapeutic effect or the patient’s health can be used to eliminate the interference of psychological factors and other confounding factors so as to accurately assess the net therapeutic efficacy of the traditional Chinese medicine. Fifth, international multi-center clinical studies should be conducted to expand the scope of research to different races and geographical areas. Cooperate with foreign medical institutions or research institutes to include patients of different races in the study. At the same time, fully consider the differences in cultural and medical environments and make adaptive adjustments to the research program to ensure the smooth conduct of the study. Sixth, a unified registration platform for Chinese medicine clinical research should be established, requiring all Chinese medicine clinical research to be registered and research protocols submitted before they are carried out, as well as strengthening the tracking and management of registered research and stipulating that research results can only be published after registration, in order to avoid publication bias caused by prioritizing positive results for publication. Seventh, the safety index system for clinical research in Chinese medicine should be improved. In addition to common indicators such as gastrointestinal, skin, and liver functions, it should also include related indicators such as renal function, cardiovascular function, and blood system so as to comprehensively assess the safety of Chinese medicine prescriptions. The safety of TCM formulas is comprehensively assessed through regular follow-up and dynamic monitoring during the research process to detect, handle, and record adverse events in a timely manner.

## 5 Conclusion

In conclusion, this study suggests that the integration of QWBZS with conventional treatment offers greater benefits compared to conventional treatment alone in managing T2DM, further enhancing glucose and lipid metabolism and ameliorating insulin resistance. QWBZS alone can also play a role in regulating glucose, but its effect on lipid metabolism and insulin resistance is not clear. Meanwhile, QWBZS improves the overall effective rate of clinical treatment and is relatively safe. Nevertheless, given the limited number of included studies, the small sample size, the poor methodological quality, and the low quality of the evidence, in particular the inclusion of studies with unreported or under-reported key aspects such as blinding and allocation concealment, this greatly affects the quality of the studies and the reliability of the results. Therefore the evidence from this study remains inconclusive and should be used with caution. When treating T2DM in the clinic, it is still necessary to consider the patient’s overall condition to develop a more appropriate and comprehensive treatment strategy. There is still a need for more high-quality, large-sample, multicenter, randomized, double-blind, and placebo-controlled randomized controlled trials in the future, and these studies need to strictly follow better trial design criteria to obtain more reliable conclusions to provide stronger evidence for the clinical application of QWBZS in the treatment of T2DM. In addition, the mechanism of action of QWBZS in the treatment of T2DM has not been elucidated yet. Preliminary studies have found that QWBZS has a good regulating effect on the function of the spleen and stomach as well as the intestinal flora in patients with T2DM, and in the future, we can use the multiomics technology to further study the specific mechanism of action of QWBZS to improve the improvement of T2DM by regulating the intestinal flora from the perspective of genes, transcripts, and metabolic levels.

## Data Availability

The original contributions presented in the study are included in the article/Supplementary Material, further inquiries can be directed to the corresponding authors.

## References

[B1] Aarnoutse R. Ziemons J. Penders J. Rensen S. S. de Vos-Geelen J. Smidt M. L. (2019). The clinical link between human intestinal microbiota and systemic cancer therapy. Int. J. Mol. Sci. 20 (17), 4145. 10.3390/ijms20174145 31450659 PMC6747354

[B2] Abulizi A. Cardone R. L. Stark R. Lewandowski S. L. Zhao X. Hillion J. (2020). Multi-tissue acceleration of the mitochondrial phosphoenolpyruvate cycle improves whole-body metabolic health. Cell Metab. 32 (5), 751–766. 10.1016/j.cmet.2020.10.006 33147485 PMC7679013

[B3] Alexander J. L. Wilson I. D. Teare J. Marchesi J. R. Nicholson J. K. Kinross J. M. (2017). Gut microbiota modulation of chemotherapy efficacy and toxicity. Nat. Rev. Gastroenterology and Hepatology 14 (6), 356–365. 10.1038/nrgastro.2017.20 28270698

[B4] Aloke C. Egwu C. O. Aja P. M. Obasi N. A. Chukwu J. Akumadu B. O. (2022). Current advances in the management of diabetes mellitus. Biomedicines 10 (10), 2436. 10.3390/biomedicines10102436 36289697 PMC9599361

[B5] Association A. D. (2021). 9. Pharmacologic approaches to glycemic treatment: *Standards of medical Care in diabetes—2021* . Diabetes Care 44 (Suppl. ment_1), S111–S124. 10.2337/dc21-S009 33298420

[B6] Bai L. Gao J. Wei F. Zhao J. Wang D. Wei J. (2018). Therapeutic potential of ginsenosides as an adjuvant treatment for diabetes. Front. Pharmacol. 9, 423. 10.3389/fphar.2018.00423 29765322 PMC5938666

[B7] Balooch Hasankhani M. Mirzaei H. Karamoozian A. (2023). Global trend analysis of diabetes mellitus incidence, mortality, and mortality-to-incidence ratio from 1990 to 2019. Sci. Rep. 13 (1), 21908. 10.1038/s41598-023-49249-0 38081899 PMC10713611

[B8] Bhatti J. S. Sehrawat A. Mishra J. Sidhu I. S. Navik U. Khullar N. (2022). Oxidative stress in the pathophysiology of type 2 diabetes and related complications: current therapeutics strategies and future perspectives. Free Radic. Biol. Med. 184, 114–134. 10.1016/j.freeradbiomed.2022.03.019 35398495

[B9] Bheda P. (2020). Metabolic transcriptional memory. Mol. Metab. 38, 100955. 10.1016/j.molmet.2020.01.019 32240621 PMC7300383

[B10] Burcelin R. Garidou L. Pomié C. (2012). Immuno-microbiota cross and talk: the new paradigm of metabolic diseases. Seminars Immunol. 24 (1), 67–74. 10.1016/j.smim.2011.11.011 22265028

[B11] Chait A. den Hartigh L. J. (2020). Adipose tissue distribution, inflammation and its metabolic consequences, including diabetes and cardiovascular disease. Front. Cardiovasc. Med. 7, 22. 10.3389/fcvm.2020.00022 32158768 PMC7052117

[B12] Chen X. Yu J. Shi J. (2018). Management of diabetes mellitus with puerarin, a natural isoflavone from*Pueraria lobata* . Am. J. Chin. Med. 46 (08), 1771–1789. 10.1142/S0192415X18500891 30525896

[B13] Eissele R. Göke R. Willemer S. Harthus H. P. Vermeer H. Arnold R. (1992). Glucagon‐like peptide‐1 cells in the gastrointestinal tract and pancreas of rat, pig and man. Eur. J. Clin. Investigation 22 (4), 283–291. 10.1111/j.1365-2362.1992.tb01464.x 1499644

[B14] Entezari M. Hashemi D. Taheriazam A. Zabolian A. Mohammadi S. Fakhri F. (2022). AMPK signaling in diabetes mellitus, insulin resistance and diabetic complications: a pre-clinical and clinical investigation. Biomed. and Pharmacother. 146, 112563. 10.1016/j.biopha.2021.112563 35062059

[B15] Everard A. Cani P. D. (2013). Diabetes, obesity and gut microbiota. Best Pract. and Res. Clin. Gastroenterology 27 (1), 73–83. 10.1016/j.bpg.2013.03.007 23768554

[B16] Fan Q. (2019). Clinical observation on invigorating spleen for eliminating dampness method in the treatment of type 2 diabetes and its influence on GLP-1 level. Chin. Med. Mod. Distance Educ. China 17 (22), 89–91. 10.3969/j.issn.1672-2779.2019.22.036

[B17] Feng X. Chen Y.-T. Li L.-Y. Sun Y.-P. Wang H.-R. Zhang L.-T. (2020). Preparation, evaluation and metabolites study in rats of novel Isoginkgetin-loaded TPGS/soluplus mixed nanomicelles. J. Food Drug Analysis 28 (2), 309–321. 10.38212/2224-6614.1065 PMC9261864 35696106

[B18] Gao J. Wang J. Xue Y. Sun J. Wang D. Han K. (2024). Effect of qiwei baizhusan on cognitive dysfunction in rats with diabetic encephalopathy based on PI3K/Akt/GSK-3β signaling pathway. Chin. J. Exp. Traditional Med. Formulae 30 (3), 10–17. 10.13422/j.cnki.syfjx.20231163

[B19] Guo K. Peng M. Peng X. Hui H. Tan Z. (2018). Effects of Qiweibaizhu powder on the intestinal bacterial diversity in dysbacteriotic diarrhea mice. Microbiol. China 45 (7), 1470–1478. 10.13344/j.microbiol.china.170751

[B20] Guo K. Tan Z. Xie M. She Y. Wang X. (2015). The synergic effect of ultra-micro powder Qiweibaizhusan combined with yeast on dysbacteriotic diarrhea mice. J. Appl. Environ. Biol. 21 (1), 61–67. 10.3724/SP.J.1145.2013.10002

[B21] He X. Hu Q. (2014). Invigorating spleen-yiqi for eliminating dampness method on GLP-1 in patients with type 2 diabetes mellitus (spleen deficiency with dampness). Liaoning J. Traditional Chin. Med. 41 (09), 1903–1906. 10.13192/j.issn.1000-1719.2014.09.046

[B22] Hong C. Schüffler A. Kauhl U. Cao J. Wu C.-F. Opatz T. (2017). Identification of NF-κB as determinant of posttraumatic stress disorder and its inhibition by the Chinese herbal remedy free and easy wanderer. Front. Pharmacol. 8, 181. 10.3389/fphar.2017.00181 28428751 PMC5382210

[B23] Jiang J. Guo K. Long L. Li D. Tan Z. Yuan Z. (2013). Inhibiting effect of ultra-micro powder qiweibaizhusan on bacteria *in vitro* . Chin. J. Inf. Traditional Chin. Med. 20 (11), 28–30. 10.3969/j.issn.1005-5304.2013.11.011

[B24] Jurjus A. Eid A. Al Kattar S. Zeenny M. N. Gerges-Geagea A. Haydar H. (2016). Inflammatory bowel disease, colorectal cancer and type 2 diabetes mellitus: the links. BBA Clin. 5, 16–24. 10.1016/j.bbacli.2015.11.002 27051585 PMC4802401

[B25] Li F. Jiang C. Krausz K. W. Li Y. Albert I. Hao H. (2013). Microbiome remodelling leads to inhibition of intestinal farnesoid X receptor signalling and decreased obesity. Nat. Commun. 4 (1), 2384. 10.1038/ncomms3384 24064762 PMC6595219

[B26] Li Y. Teng D. Shi X. Qin G. Qin Y. Quan H. (2020). Prevalence of diabetes recorded in mainland China using 2018 diagnostic criteria from the American Diabetes Association: national cross sectional study. BMJ 369, m997. 10.1136/bmj.m997 32345662 PMC7186854

[B27] Li Z. (2014). Study on the clinical efficacy of Qiwei Baizhu San in the treatment of Wasting and thirst disorders. Asia-Pacific Tradit. Med. 10 (07), 111–112.

[B28] Liang J. Xiao J. Pan W. Luo L. Peng L. (2023). Clinical observation of qiweibazhi powder combined with western medicine in the treatment of obesity diabetes mellitus type 2. Chin. J. Ethnomedicine Ethnopharmacy 32 (12), 103–105.

[B29] Liao H. Chen G. Dai R. (2024). Efficacy of Qiwei Baizhu San combined with conventional Western medicine in the treatment of patients with type 2 diabetes. Shenzhen J. Integr. Traditional Chin. West. Med. 34 (05), 37–39. 10.16458/j.cnki.1007-0893.2024.05.010

[B30] Liao S. (2022). Clinical effect of adding and subtracting qiwei baizhu san in the treatment of paediatric diarrhoea of spleen deficiency type. Inn. Mong. J. Traditional Chin. Med. 41 (9), 5–6. 10.16040/j.cnki.cn15-1101.2022.09.023

[B31] Lin W. (2022). Analysis of the effect of qiweibaizhu powder modified and subtracted as an adjuvant treatment on diabetes with deficiency of both qi and yin. Diabetes New World 25 (02), 96–98+110. 10.16658/j.cnki.1672-4062.2022.02.096

[B32] Liu S. Li J. Wang Y. (2020). Regulatory effect of qiwei baizhusan on liver tissue insulin PI3K/Akt signal pathway in diabetic mice. Chin. J. Exp. Traditional Med. Formulae 26 (20), 153–160. 10.13422/j.cnki.syfjx.20201903

[B33] Liu S. Qi Z. Wang Y. Fu Q. Li J. (2024). Effects of qiwei baizhu powder on inflammatory factors and intestinal flora in diabetics mice. Liaoning J. Chin. Med. 51 (7), 188-193+222–224. 10.13192/j.issn.1000-1719.2024.07.049

[B34] Liu W. (2020). Clinical observation of Qiwei Baizhu San combined with metformin in the treatment of asymptomatic hyperuricemia complicated with type 2 diabetes mellitus. Diabetes New World 23 (01), 52–53. 10.16658/j.cnki.1672-4062.2020.01.052

[B35] Liu X. Zhou L. Liu Y. Liu R. Ma X. Wang Z. (2022). Effects of qiwei baizhu san on glycolipid metabolism, insulin resistance and gastric starvation hormone in diabetic KKAy mice. Lishizhen Med. Materia Medica Res. 33 (11), 2630–2632. 10.3969/j.issn.1008-0805.2022.11.18

[B36] Luo X. (2024). Observation of the clinical efficacy of Qiwei Baizhu San combined with hypoglycemic drugs in the treatment of spleen and stomach qi deficiency diabetes. China Sci. Technol. Periodicals Database Med. (6).

[B37] Lv J. (2022) “To explore the *in vitro* study of Qiwei Baizhu Powder improving insulin resistance based on AMPK/PI3K/Akt signaling pathway and CFTR,” in Master's degree thesis (MSc). Shandong University of Traditional Chinese Medicine.

[B38] Mannucci E. Ognibene A. Cremasco F. Bardini G. Mencucci A. Pierazzuoli E. (2000). Glucagon‐like peptide (GLP)‐1 and leptin concentrations in obese patients with Type 2 diabetes mellitus. Diabet. Med. 17 (10), 713–719. 10.1046/j.1464-5491.2000.00367.x 11110504

[B39] Meng H. Liu H. Jiang P. Tan J. Wang Y. (2022). Clinical study on modified Qiwei Baizhu Powder combined with conventional therapy in the treatment of type 2 diabetes mellitus with abnormal lipid metabolism. Int. J. Traditional Chin. Med. 44 (10), 1117–1121. 10.3760/cma.j.cn115398-20211028-00317

[B40] Mohammad S. Thiemermann C. (2020). Role of metabolic endotoxemia in systemic inflammation and potential interventions. Front. Immunol. 11, 594150. 10.3389/fimmu.2020.594150 33505393 PMC7829348

[B41] Moher D. Liberati A. Tetzlaff J. Altman D. G. Group P. (2009). Preferred reporting Items for systematic reviews and meta-analyses: the PRISMA statement. PLoS Med. 6 (7), e1000097. 10.1371/journal.pmed.1000097 19621072 PMC2707599

[B42] Niu Y. (2014). An effective analysis of treating 84 cases of diabetes II with Qiwei Baizhu San. Clin. J. Chin. Med. 6 (31), 59–60. 10.3969/j.issn.1674-7860.2014.31.031

[B43] Page M. J. McKenzie J. E. Bossuyt P. M. Boutron I. Hoffmann T. C. Mulrow C. D. (2021). The PRISMA 2020 statement: an updated guideline for reporting systematic reviews. BMJ 372, n71. 10.1136/bmj.n71 33782057 PMC8005924

[B44] Patil S. R. Chavan A. B. Patel A. M. Chavan P. D. Bhopale J. V. (2023). A review on diabetes mellitus its types, pathophysiology, epidermiology and its global burden. J. Res. Appl. Sci. Biotechnol. 2 (4), 73–79. 10.55544/jrasb.2.4.9

[B45] Perry R. J. Peng L. Barry N. A. Cline G. W. Zhang D. Cardone R. L. (2016). Acetate mediates a microbiome-brain-β-cell axis to promote metabolic syndrome. Nature 534 (7606), 213–217. 10.1038/nature18309 27279214 PMC4922538

[B46] Pu J. a. Zheng T. Li C. Peng X. Shu L. Tan Z. (2017). Effect of sucrose on qiweibaishusan to intestinal bacteria diversity in dysbacteriotic diarrhea mice. J. Food Sci. Biotechnol. 36 (6), 583–589. 10.3969/j.issn.1673-1689.2017.06.004

[B47] Qian Y. (2022). Clinical observation on improving the insulin resistance of spleen deficiency type 2 diabetes mellitus with Jiawei Qiwei Baizhu San.

[B48] Qian Y. Yang J. Yu J. (2000). Key to diagnosis and treatment of Children’s diseases (Tianjin: Tianjin Science and Technology Press).

[B49] Shi Q. Nong K. Vandvik P. O. Guyatt G. H. Schnell O. Rydén L. (2023). Benefits and harms of drug treatment for type 2 diabetes: systematic review and network meta-analysis of randomised controlled trials. BMJ 381, e074068. 10.1136/bmj-2022-074068 37024129 PMC10077111

[B50] Sun H. Saeedi P. Karuranga S. Pinkepank M. Ogurtsova K. Duncan B. B. (2022). IDF Diabetes Atlas: global, regional and country-level diabetes prevalence estimates for 2021 and projections for 2045. DIABETES Res. Clin. Pract. 183, 109119. 10.1016/j.diabres.2021.109119 34879977 PMC11057359

[B51] Tan D. Tseng H. H. L. Zhong Z. Wang S. Vong C. T. Wang Y. (2022). Glycyrrhizic acid and its derivatives: promising candidates for the management of type 2 diabetes mellitus and its complications. Int. J. Mol. Sci. 23 (19), 10988. 10.3390/ijms231910988 36232291 PMC9569462

[B52] Testa R. Bonfigli A. R. Prattichizzo F. La Sala L. De Nigris V. Ceriello A. (2017). The “metabolic memory” theory and the early treatment of hyperglycemia in prevention of diabetic complications. Nutrients 9 (5), 437. 10.3390/nu9050437 28452927 PMC5452167

[B53] Wang A. Han Y. Yuan Y. (2022). Exploration on the eff ects of fortune eupatorium herb combined with Qiwei Baizhu San on the treatment of patients with type 2 diabetes. J. Changchun Univ. Chin. Med. 38 (01), 75–79. 10.13463/j.cnki.cczyy.2022.01.018

[B54] Wang C. (2023). Based on the TLR4/NF-κB signaling pathway to observe the clinical efficacy of Qiwei Baizhu powder on type 2 diabetes

[B55] Wang C. Miao X. Guo L. Han X. Liu L. Qian Y. (2023). Effects of Qiwei Baizhu San on intestinal barrier and TLR4/NF-κB signaling pathway in diabetic rats. Microbiol. China 50 (1), 313–323. 10.13344/j.microbiol.china.220295

[B56] Wang Y. Dong Z. Zhang L. Wang K. Liu P. Wang P. (2020). Evaluation research of gradient ethanol extract from sijunzi decoction intervening glucolipid metabolic disorder of diabetes 2 mice. Pharmacol. Clin. Chin. Materia Medica 36 (4), 93–98. 10.13412/j.cnki.zyyl.2020.04.006

[B57] Wei J. Tian J. Tang C. Fang X. Miao R. Wu H. (2022). The influence of different types of diabetes on vascular complications. J. Diabetes Res. 2022, 3448618–3448712. 10.1155/2022/3448618 35242879 PMC8888068

[B58] Weis S. Carlos A. R. Moita M. R. Singh S. Blankenhaus B. Cardoso S. (2017). Metabolic adaptation establishes disease tolerance to sepsis. Cell 169 (7), 1263–1275. 10.1016/j.cell.2017.05.031 28622511 PMC5480394

[B59] Wong S. L. Priestman A. Holmes D. T. (2014). Recurrent hypoglycemia from insulin autoimmune syndrome. J. General Intern. Med. 29 (1), 250–254. 10.1007/s11606-013-2588-9 PMC3889959 23979685

[B60] Wu Q. Yan Z. a. He Y. (1994). Golden mirror of the medical tradition (Beijing: China Traditional Chinese Medicine Press).

[B61] Yang Q. Hua X. Zhou S. (2019). Effect of qiwei baizhu powder on oxidative stress in diabetic rats. Acta Chin. Med. 34 (11), 2408–2411. 10.16368/j.issn.1674-8999.2019.11.555

[B62] Yang Y. Zeng J. (2023). Effect of Qiwei Baizhu Powder combined with Kangfuxin Liquid on postoperative wound healing in patients of type 2 diabetes mellitus with anal fistula complicated. J. Shanxi Univ. Chin. Med. 24(01), 67–70. 10.19763/j.cnki.2096-7403.2023.01.11

[B63] Ye J. Wu Y. Yang S. Zhu D. Chen F. Chen J. (2023). The global, regional and national burden of type 2 diabetes mellitus in the past, present and future: a systematic analysis of the Global Burden of Disease Study 2019. Front. Endocrinol. 14, 1192629. 10.3389/fendo.2023.1192629 PMC10376703 37522116

[B64] Zhang P. Li T. Wu X. Nice E. C. Huang C. Zhang Y. (2020). Oxidative stress and diabetes: antioxidative strategies. Front. Med. 14 (5), 583–600. 10.1007/s11684-019-0729-1 32248333

