# Supplementary material for: The efficacy and safety of qiwei baizhu san in the treatment of type 2 diabetes mellitus: a systematic review and meta-analysis
Source: Front Pharmacol. 2025 Jan 7;15:1501990. doi: 10.3389/fphar.2024.1501990 (PMC11752898; doi:10.3389/fphar.2024.1501990)
Supplement: Supplementary file 2 [file Table1.docx]

Supplementary Material

**Contents Page**

[1 Supplementary Material S1. PRISMA 2020 checklist 2](#_Toc177902356)

[2 Supplementary Material S2. Database and Search Strategies 8](#_Toc177902357)

[2.1 PubMed 8](#_Toc177902358)

[2.2 Embase 10](#_Toc177902359)

[2.3 Cochrane 11](#_Toc177902360)

[2.4 WOS 11](#_Toc177902361)

[2.5 CNKI 12](#_Toc177902362)

[2.6 Wan Fang 13](#_Toc177902363)

[2.7 VIP 14](#_Toc177902364)

[2.8 CBM 15](#_Toc177902365)

[2.9 ClinicalTrials.gov 16](#_Toc177902366)

[2.10 Chinese Clinical Trial Registry 16](#_Toc177902367)

[3 Supplementary Material S3. The procedure for preliminary screening of the literature 17](#_Toc177902368)

[4 Supplementary Material S4. Literature excluded after reading the full text and reasons 17](#_Toc177902369)

[4.1 The diagnosis in these studies was unclear or non-type 2 diabetes 17](#_Toc177902370)

[4.2 The intervention in these studies was not QWBZS or was combined with other TCM therapies 19](#_Toc177902371)

[4.3 The study design was not a randomized controlled trial 19](#_Toc177902372)

[4.4 Lack of sufficient data results 19](#_Toc177902373)

[4.5 Duplicate literature 20](#_Toc177902374)

[4.6 Subjects may have severe renal impairment 20](#_Toc177902375)

[5 Supplementary Material S5. The components of the original QWBZS or modified QWBZS 20](#_Toc177902376)

[6 Supplementary Material S6. Subgroup analysis 24](#_Toc177902377)

[6.1 Subgroup analysis of FBG for QWBZS combined with conventional treatment vs. conventional treatment 24](#_Toc177902378)

[6.2 Subgroup analysis of 2hPG for QWBZS combined with conventional treatment vs. conventional treatment 24](#_Toc177902379)

[6.3 Subgroup analysis of HbA1c for QWBZS combined with conventional treatment vs. conventional treatment 25](#_Toc177902380)

[7 Supplementary Material S7. Sensitivity analysis 25](#_Toc177902381)

[8 Supplementary Material S8. Labbe plot of overall effective rate 26](#_Toc177902382)

[9 Supplementary Material S9. Egger’s test of FBG, 2hPG and overall effective rate 27](#_Toc177902383)

[10 Supplementary Material S10. Filled funnel plot 28](#_Toc177902384)

[11 Supplementary Material S11. Assessment of evidence quality for each outcome 30](#_Toc177902385)

# Supplementary Material S1. PRISMA 2020 checklist

| **Section and Topic** | **Item #** | **Checklist item** | **Location where item is reported** |
| --- | --- | --- | --- |
| **TITLE** | | |  |
| Title | 1 | Identify the report as a systematic review. | P1 |
| **ABSTRACT** | | |  |
| Abstract | 2 | See the PRISMA 2020 for Abstracts checklist. | P1-2 |
| **INTRODUCTION** | | |  |
| Rationale | 3 | Describe the rationale for the review in the context of existing knowledge. | P2-3 |
| Objectives | 4 | Provide an explicit statement of the objective(s) or question(s) the review addresses. | P3 |
| **METHODS** | | |  |
| Eligibility criteria | 5 | Specify the inclusion and exclusion criteria for the review and how studies were grouped for the syntheses. | P4-5 |
| Information sources | 6 | Specify all databases, registers, websites, organisations, reference lists and other sources searched or consulted to identify studies. Specify the date when each source was last searched or consulted. | P4 |
| Search strategy | 7 | Present the full search strategies for all databases, registers and websites, including any filters and limits used. | Supplementary Material S2 |
| Selection process | 8 | Specify the methods used to decide whether a study met the inclusion criteria of the review, including how many reviewers screened each record and each report retrieved, whether they worked independently, and if applicable, details of automation tools used in the process. | P5 |
| Data collection process | 9 | Specify the methods used to collect data from reports, including how many reviewers collected data from each report, whether they worked independently, any processes for obtaining or confirming data from study investigators, and if applicable, details of automation tools used in the process. | P5 |
| Data items | 10a | List and define all outcomes for which data were sought. Specify whether all results that were compatible with each outcome domain in each study were sought (e.g. for all measures, time points, analyses), and if not, the methods used to decide which results to collect. | P5 |
|  | 10b | List and define all other variables for which data were sought (e.g. participant and intervention characteristics, funding sources). Describe any assumptions made about any missing or unclear information. | P5 |
| Study risk of bias assessment | 11 | Specify the methods used to assess risk of bias in the included studies, including details of the tool(s) used, how many reviewers assessed each study and whether they worked independently, and if applicable, details of automation tools used in the process. | P5 |
| Effect measures | 12 | Specify for each outcome the effect measure(s) (e.g. risk ratio, mean difference) used in the synthesis or presentation of results. | P5-6 |
| Synthesis methods | 13a | Describe the processes used to decide which studies were eligible for each synthesis (e.g. tabulating the study intervention characteristics and comparing against the planned groups for each synthesis (item #5)). | P5 |
|  | 13b | Describe any methods required to prepare the data for presentation or synthesis, such as handling of missing summary statistics, or data conversions. | P5 |
|  | 13c | Describe any methods used to tabulate or visually display results of individual studies and syntheses. | P5 |
|  | 13d | Describe any methods used to synthesize results and provide a rationale for the choice(s). If meta-analysis was performed, describe the model(s), method(s) to identify the presence and extent of statistical heterogeneity, and software package(s) used. | P5 |
|  | 13e | Describe any methods used to explore possible causes of heterogeneity among study results (e.g. subgroup analysis, meta-regression). | P5 |
|  | 13f | Describe any sensitivity analyses conducted to assess robustness of the synthesized results. | P5 |
| Reporting bias assessment | 14 | Describe any methods used to assess risk of bias due to missing results in a synthesis (arising from reporting biases). | P5 |
| Certainty assessment | 15 | Describe any methods used to assess certainty (or confidence) in the body of evidence for an outcome. | P6 |
| **RESULTS** | | |  |
| Study selection | 16a | Describe the results of the search and selection process, from the number of records identified in the search to the number of studies included in the review, ideally using a flow diagram. | P6, Figure 1 |
|  | 16b | Cite studies that might appear to meet the inclusion criteria, but which were excluded, and explain why they were excluded. | Supplementary Material S4 |
| Study characteristics | 17 | Cite each included study and present its characteristics. | P6-7, Table 1, Supplementary Material S5 |
| Risk of bias in studies | 18 | Present assessments of risk of bias for each included study. | P7, Figure 2 |
| Results of individual studies | 19 | For all outcomes, present, for each study: (a) summary statistics for each group (where appropriate) and (b) an effect estimate and its precision (e.g. confidence/credible interval), ideally using structured tables or plots. | P7-11 |
| Results of syntheses | 20a | For each synthesis, briefly summarise the characteristics and risk of bias among contributing studies. | P7-11 |
|  | 20b | Present results of all statistical syntheses conducted. If meta-analysis was done, present for each the summary estimate and its precision (e.g. confidence/credible interval) and measures of statistical heterogeneity. If comparing groups, describe the direction of the effect. | P7-11 |
|  | 20c | Present results of all investigations of possible causes of heterogeneity among study results. | P7-11 |
|  | 20d | Present results of all sensitivity analyses conducted to assess the robustness of the synthesized results. | P7-11 |
| Reporting biases | 21 | Present assessments of risk of bias due to missing results (arising from reporting biases) for each synthesis assessed. | P11 |
| Certainty of evidence | 22 | Present assessments of certainty (or confidence) in the body of evidence for each outcome assessed. | P11, Supplementary Material S11 |
| **DISCUSSION** | | |  |
| Discussion | 23a | Provide a general interpretation of the results in the context of other evidence. | P11-13 |
|  | 23b | Discuss any limitations of the evidence included in the review. | P16-17 |
|  | 23c | Discuss any limitations of the review processes used. | P16-17 |
|  | 23d | Discuss implications of the results for practice, policy, and future research. | P11-17 |
| **OTHER INFORMATION** | | |  |
| Registration and protocol | 24a | Provide registration information for the review, including register name and registration number, or state that the review was not registered. | P4, CRD42024576129 |
|  | 24b | Indicate where the review protocol can be accessed, or state that a protocol was not prepared. | - |
|  | 24c | Describe and explain any amendments to information provided at registration or in the protocol. | - |
| Support | 25 | Describe sources of financial or non-financial support for the review, and the role of the funders or sponsors in the review. | P18 |
| Competing interests | 26 | Declare any competing interests of review authors. | P17 |
| Availability of data, code and other materials | 27 | Report which of the following are publicly available and where they can be found: template data collection forms; data extracted from included studies; data used for all analyses; analytic code; any other materials used in the review. | P18 |

*From:*  Page MJ, McKenzie JE, Bossuyt PM, Boutron I, Hoffmann TC, Mulrow CD, et al. The PRISMA 2020 statement: an updated guideline for reporting systematic reviews. BMJ 2021;372:n71. doi: 10.1136/bmj.n71

# Supplementary Material S2. Database and Search Strategies

## PubMed

The retrieval of the PubMed database was conducted on August 2, 2024, and no records were retrieved.

| Search | Query | Results |
| --- | --- | --- |
| #5 | ((Diabetes Mellitus, Type 2[MeSH Terms]) OR ("adult onset diabetes"[Title/Abstract] OR "adult onset diabetes mellitus"[Title/Abstract] OR "Adult-Onset Diabetes Mellitus"[Title/Abstract] OR "diabetes mellitus type 2"[Title/Abstract] OR "diabetes mellitus type ii"[Title/Abstract] OR "Diabetes Mellitus, Adult Onset"[Title/Abstract] OR "Diabetes Mellitus, Adult-Onset"[Title/Abstract] OR "Diabetes Mellitus, Ketosis Resistant"[Title/Abstract] OR "Diabetes Mellitus, Ketosis-Resistant"[Title/Abstract] OR "diabetes mellitus, maturity onset"[Title/Abstract] OR "Diabetes Mellitus, Maturity-Onset"[Title/Abstract] OR "diabetes mellitus, non insulin dependent"[Title/Abstract] OR "Diabetes Mellitus, Noninsulin Dependent"[Title/Abstract] OR "Diabetes Mellitus, Noninsulin-Dependent"[Title/Abstract] OR "diabetes mellitus, non-insulin-dependent"[Title/Abstract] OR "Diabetes Mellitus, Slow Onset"[Title/Abstract] OR "Diabetes Mellitus, Slow-Onset"[Title/Abstract] OR "Diabetes Mellitus, Stable"[Title/Abstract] OR "Diabetes Mellitus, Type 2"[Title/Abstract] OR "diabetes mellitus, type II"[Title/Abstract] OR "diabetes type 2"[Title/Abstract] OR "diabetes type II"[Title/Abstract] OR "diabetes, adult onset"[Title/Abstract] OR "Diabetes, Maturity-Onset"[Title/Abstract] OR "Diabetes, Type 2"[Title/Abstract] OR "dm 2"[Title/Abstract] OR "insulin independent diabetes"[Title/Abstract] OR "insulin independent diabetes mellitus"[Title/Abstract] OR "ketosis resistant diabetes mellitus"[Title/Abstract] OR "Ketosis-Resistant Diabetes Mellitus"[Title/Abstract] OR "maturity onset diabetes"[Title/Abstract] OR "maturity onset diabetes mellitus"[Title/Abstract] OR "Maturity-Onset Diabetes"[Title/Abstract] OR "Maturity-Onset Diabetes Mellitus"[Title/Abstract] OR "MODY"[Title/Abstract] OR "niddm"[Title/Abstract] OR "NIDDM (non insulin dependent diabetes mellitus)"[Title/Abstract] OR "non insulin dependent (type 2) diabetes mellitus"[Title/Abstract] OR "non insulin dependent diabetes"[Title/Abstract] OR "non insulin dependent diabetes mellitus"[Title/Abstract] OR "noninsulin dependent (type 2) diabetes mellitus"[Title/Abstract] OR "noninsulin dependent diabetes"[Title/Abstract] OR "noninsulin dependent diabetes mellitus"[Title/Abstract] OR "Noninsulin-Dependent Diabetes Mellitus"[Title/Abstract] OR "non-insulin-dependent diabetes mellitus"[Title/Abstract] OR "Slow-Onset Diabetes Mellitus"[Title/Abstract] OR "Stable Diabetes Mellitus"[Title/Abstract] OR "T2DM"[Title/Abstract] OR "TIIDM"[Title/Abstract] OR "type 2 (insulin independent) diabetes"[Title/Abstract] OR "type 2 diabetes"[Title/Abstract] OR "type 2 diabetes mellitus"[Title/Abstract] OR "Type 2 Diabetes Mellitus"[Title/Abstract] OR "type II diabetes"[Title/Abstract] OR "type II diabetes mellitus"[Title/Abstract])) AND ("Atractylodes Macrocephala Decoction with Seven Ingredients"[Title/Abstract] OR "Formula of Seven Ingredients with Atractylodes "[Title/Abstract] OR "Qiwei Baizhu"[Title/Abstract] OR "Qiwei Baizhu Powder"[Title/Abstract] OR "Qiwei Baizhu San"[Title/Abstract] OR "Qiweibaizhu"[Title/Abstract] OR "Qiweibaizhu Powder"[Title/Abstract] OR "Qiweibaizhu San"[Title/Abstract] OR "Seven flavors of atractylodes powder"[Title/Abstract] OR "Seven-Flavor Atractylodes Formula"[Title/Abstract] OR "Seven-Ingredient Atractylodes Powder"[Title/Abstract] OR "Seven-ingredient Bai Zhu Powder"[Title/Abstract]) | 0 |
| #4 | "Atractylodes Macrocephala Decoction with Seven Ingredients"[Title/Abstract] OR "Formula of Seven Ingredients with Atractylodes "[Title/Abstract] OR "Qiwei Baizhu"[Title/Abstract] OR "Qiwei Baizhu Powder"[Title/Abstract] OR "Qiwei Baizhu San"[Title/Abstract] OR "Qiweibaizhu"[Title/Abstract] OR "Qiweibaizhu Powder"[Title/Abstract] OR "Qiweibaizhu San"[Title/Abstract] OR "Seven flavors of atractylodes powder"[Title/Abstract] OR "Seven-Flavor Atractylodes Formula"[Title/Abstract] OR "Seven-Ingredient Atractylodes Powder"[Title/Abstract] OR "Seven-ingredient Bai Zhu Powder"[Title/Abstract] | 16 |
| #3 | (Diabetes Mellitus, Type 2[MeSH Terms]) OR ("adult onset diabetes"[Title/Abstract] OR "adult onset diabetes mellitus"[Title/Abstract] OR "Adult-Onset Diabetes Mellitus"[Title/Abstract] OR "diabetes mellitus type 2"[Title/Abstract] OR "diabetes mellitus type ii"[Title/Abstract] OR "Diabetes Mellitus, Adult Onset"[Title/Abstract] OR "Diabetes Mellitus, Adult-Onset"[Title/Abstract] OR "Diabetes Mellitus, Ketosis Resistant"[Title/Abstract] OR "Diabetes Mellitus, Ketosis-Resistant"[Title/Abstract] OR "diabetes mellitus, maturity onset"[Title/Abstract] OR "Diabetes Mellitus, Maturity-Onset"[Title/Abstract] OR "diabetes mellitus, non insulin dependent"[Title/Abstract] OR "Diabetes Mellitus, Noninsulin Dependent"[Title/Abstract] OR "Diabetes Mellitus, Noninsulin-Dependent"[Title/Abstract] OR "diabetes mellitus, non-insulin-dependent"[Title/Abstract] OR "Diabetes Mellitus, Slow Onset"[Title/Abstract] OR "Diabetes Mellitus, Slow-Onset"[Title/Abstract] OR "Diabetes Mellitus, Stable"[Title/Abstract] OR "Diabetes Mellitus, Type 2"[Title/Abstract] OR "diabetes mellitus, type II"[Title/Abstract] OR "diabetes type 2"[Title/Abstract] OR "diabetes type II"[Title/Abstract] OR "diabetes, adult onset"[Title/Abstract] OR "Diabetes, Maturity-Onset"[Title/Abstract] OR "Diabetes, Type 2"[Title/Abstract] OR "dm 2"[Title/Abstract] OR "insulin independent diabetes"[Title/Abstract] OR "insulin independent diabetes mellitus"[Title/Abstract] OR "ketosis resistant diabetes mellitus"[Title/Abstract] OR "Ketosis-Resistant Diabetes Mellitus"[Title/Abstract] OR "maturity onset diabetes"[Title/Abstract] OR "maturity onset diabetes mellitus"[Title/Abstract] OR "Maturity-Onset Diabetes"[Title/Abstract] OR "Maturity-Onset Diabetes Mellitus"[Title/Abstract] OR "MODY"[Title/Abstract] OR "niddm"[Title/Abstract] OR "NIDDM (non insulin dependent diabetes mellitus)"[Title/Abstract] OR "non insulin dependent (type 2) diabetes mellitus"[Title/Abstract] OR "non insulin dependent diabetes"[Title/Abstract] OR "non insulin dependent diabetes mellitus"[Title/Abstract] OR "noninsulin dependent (type 2) diabetes mellitus"[Title/Abstract] OR "noninsulin dependent diabetes"[Title/Abstract] OR "noninsulin dependent diabetes mellitus"[Title/Abstract] OR "Noninsulin-Dependent Diabetes Mellitus"[Title/Abstract] OR "non-insulin-dependent diabetes mellitus"[Title/Abstract] OR "Slow-Onset Diabetes Mellitus"[Title/Abstract] OR "Stable Diabetes Mellitus"[Title/Abstract] OR "T2DM"[Title/Abstract] OR "TIIDM"[Title/Abstract] OR "type 2 (insulin independent) diabetes"[Title/Abstract] OR "type 2 diabetes"[Title/Abstract] OR "type 2 diabetes mellitus"[Title/Abstract] OR "Type 2 Diabetes Mellitus"[Title/Abstract] OR "type II diabetes"[Title/Abstract] OR "type II diabetes mellitus"[Title/Abstract]) | 258,950 |
| #2 | "adult onset diabetes"[Title/Abstract] OR "adult onset diabetes mellitus"[Title/Abstract] OR "Adult-Onset Diabetes Mellitus"[Title/Abstract] OR "diabetes mellitus type 2"[Title/Abstract] OR "diabetes mellitus type ii"[Title/Abstract] OR "Diabetes Mellitus, Adult Onset"[Title/Abstract] OR "Diabetes Mellitus, Adult-Onset"[Title/Abstract] OR "Diabetes Mellitus, Ketosis Resistant"[Title/Abstract] OR "Diabetes Mellitus, Ketosis-Resistant"[Title/Abstract] OR "diabetes mellitus, maturity onset"[Title/Abstract] OR "Diabetes Mellitus, Maturity-Onset"[Title/Abstract] OR "diabetes mellitus, non insulin dependent"[Title/Abstract] OR "Diabetes Mellitus, Noninsulin Dependent"[Title/Abstract] OR "Diabetes Mellitus, Noninsulin-Dependent"[Title/Abstract] OR "diabetes mellitus, non-insulin-dependent"[Title/Abstract] OR "Diabetes Mellitus, Slow Onset"[Title/Abstract] OR "Diabetes Mellitus, Slow-Onset"[Title/Abstract] OR "Diabetes Mellitus, Stable"[Title/Abstract] OR "Diabetes Mellitus, Type 2"[Title/Abstract] OR "diabetes mellitus, type II"[Title/Abstract] OR "diabetes type 2"[Title/Abstract] OR "diabetes type II"[Title/Abstract] OR "diabetes, adult onset"[Title/Abstract] OR "Diabetes, Maturity-Onset"[Title/Abstract] OR "Diabetes, Type 2"[Title/Abstract] OR "dm 2"[Title/Abstract] OR "insulin independent diabetes"[Title/Abstract] OR "insulin independent diabetes mellitus"[Title/Abstract] OR "ketosis resistant diabetes mellitus"[Title/Abstract] OR "Ketosis-Resistant Diabetes Mellitus"[Title/Abstract] OR "maturity onset diabetes"[Title/Abstract] OR "maturity onset diabetes mellitus"[Title/Abstract] OR "Maturity-Onset Diabetes"[Title/Abstract] OR "Maturity-Onset Diabetes Mellitus"[Title/Abstract] OR "MODY"[Title/Abstract] OR "niddm"[Title/Abstract] OR "NIDDM (non insulin dependent diabetes mellitus)"[Title/Abstract] OR "non insulin dependent (type 2) diabetes mellitus"[Title/Abstract] OR "non insulin dependent diabetes"[Title/Abstract] OR "non insulin dependent diabetes mellitus"[Title/Abstract] OR "noninsulin dependent (type 2) diabetes mellitus"[Title/Abstract] OR "noninsulin dependent diabetes"[Title/Abstract] OR "noninsulin dependent diabetes mellitus"[Title/Abstract] OR "Noninsulin-Dependent Diabetes Mellitus"[Title/Abstract] OR "non-insulin-dependent diabetes mellitus"[Title/Abstract] OR "Slow-Onset Diabetes Mellitus"[Title/Abstract] OR "Stable Diabetes Mellitus"[Title/Abstract] OR "T2DM"[Title/Abstract] OR "TIIDM"[Title/Abstract] OR "type 2 (insulin independent) diabetes"[Title/Abstract] OR "type 2 diabetes"[Title/Abstract] OR "type 2 diabetes mellitus"[Title/Abstract] OR "Type 2 Diabetes Mellitus"[Title/Abstract] OR "type II diabetes"[Title/Abstract] OR "type II diabetes mellitus"[Title/Abstract] | 212,251 |
| #1 | Diabetes Mellitus, Type 2[MeSH Terms] | 182,770 |

## Embase

The retrieval of the Embase database was conducted on August 2, 2024, and no records were retrieved.

| Search |  | Results |
| --- | --- | --- |
| #4 | (#1 OR #2) AND #3 | 0 |
| #3 | 'atractylodes macrocephala decoction with seven ingredients':ti,ab,kw OR 'formula of seven ingredients with atractylodes':ti,ab,kw OR 'qiwei baizhu':ti,ab,kw OR 'qiwei baizhu powder':ti,ab,kw OR 'qiwei baizhu san':ti,ab,kw OR 'qiweibaizhu':ti,ab,kw OR 'qiweibaizhu powder':ti,ab,kw OR 'qiweibaizhu san':ti,ab,kw OR 'seven flavors of atractylodes powder':ti,ab,kw OR 'seven-flavor atractylodes formula':ti,ab,kw OR 'seven-ingredient atractylodes powder':ti,ab,kw OR 'seven-ingredient bai zhu powder':ti,ab,kw | 19 |
| #2 | 'adult onset diabetes':ti,ab,kw OR 'adult onset diabetes mellitus':ti,ab,kw OR 'adult-onset diabetes mellitus':ti,ab,kw OR 'diabetes mellitus type 2':ti,ab,kw OR 'diabetes mellitus type ii':ti,ab,kw OR 'diabetes mellitus, adult onset':ti,ab,kw OR 'diabetes mellitus, adult-onset':ti,ab,kw OR 'diabetes mellitus, ketosis resistant':ti,ab,kw OR 'diabetes mellitus, ketosis-resistant':ti,ab,kw OR 'diabetes mellitus, maturity onset':ti,ab,kw OR 'diabetes mellitus, maturity-onset':ti,ab,kw OR 'diabetes mellitus, non insulin dependent':ti,ab,kw OR 'diabetes mellitus, noninsulin dependent':ti,ab,kw OR 'diabetes mellitus, noninsulin-dependent':ti,ab,kw OR 'diabetes mellitus, non-insulin-dependent':ti,ab,kw OR 'diabetes mellitus, slow onset':ti,ab,kw OR 'diabetes mellitus, slow-onset':ti,ab,kw OR 'diabetes mellitus, stable':ti,ab,kw OR 'diabetes mellitus, type 2':ti,ab,kw OR 'diabetes mellitus, type ii':ti,ab,kw OR 'diabetes type 2':ti,ab,kw OR 'diabetes type ii':ti,ab,kw OR 'diabetes, adult onset':ti,ab,kw OR 'diabetes, maturity-onset':ti,ab,kw OR 'diabetes, type 2':ti,ab,kw OR 'dm 2':ti,ab,kw OR 'insulin independent diabetes':ti,ab,kw OR 'insulin independent diabetes mellitus':ti,ab,kw OR 'ketosis resistant diabetes mellitus':ti,ab,kw OR 'ketosis-resistant diabetes mellitus':ti,ab,kw OR 'maturity onset diabetes':ti,ab,kw OR 'maturity onset diabetes mellitus':ti,ab,kw OR 'maturity-onset diabetes':ti,ab,kw OR 'maturity-onset diabetes mellitus':ti,ab,kw OR 'mody':ti,ab,kw OR 'niddm':ti,ab,kw OR 'niddm (non insulin dependent diabetes mellitus)':ti,ab,kw OR 'non insulin dependent (type 2) diabetes mellitus':ti,ab,kw OR 'non insulin dependent diabetes':ti,ab,kw OR 'non insulin dependent diabetes mellitus':ti,ab,kw OR 'noninsulin dependent (type 2) diabetes mellitus':ti,ab,kw OR 'noninsulin dependent diabetes':ti,ab,kw OR 'noninsulin dependent diabetes mellitus':ti,ab,kw OR 'noninsulin-dependent diabetes mellitus':ti,ab,kw OR 'non-insulin-dependent diabetes mellitus':ti,ab,kw OR 'slow-onset diabetes mellitus':ti,ab,kw OR 'stable diabetes mellitus':ti,ab,kw OR 't2dm':ti,ab,kw OR 'tiidm':ti,ab,kw OR 'type 2 (insulin independent) diabetes':ti,ab,kw OR 'type 2 diabetes':ti,ab,kw OR 'type 2 diabetes mellitus':ti,ab,kw OR 'type ii diabetes':ti,ab,kw OR 'type ii diabetes mellitus':ti,ab,kw | 382,132 |
| #1 | 'non insulin dependent diabetes mellitus'/exp OR 'non insulin dependent diabetes mellitus' | 384,366 |


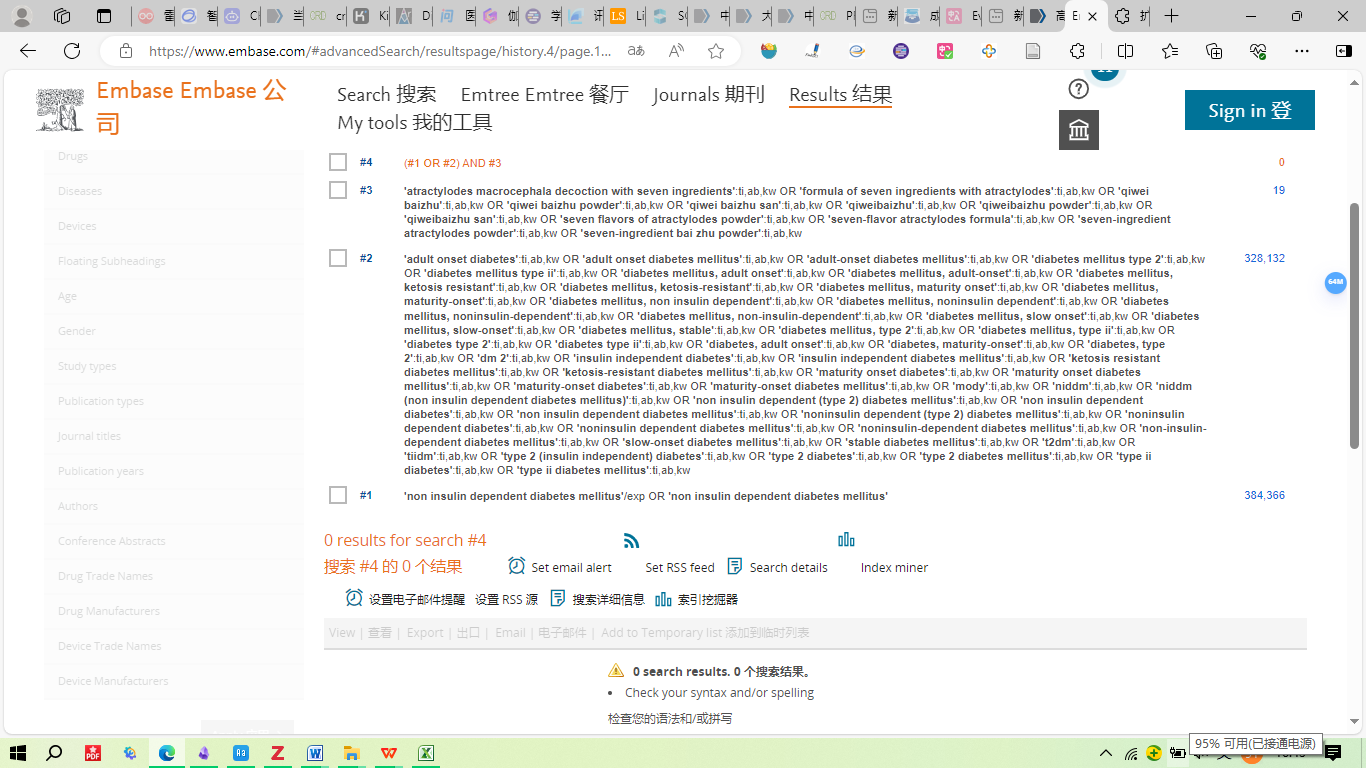


## Cochrane

The retrieval of the Cochrane Library was conducted on August 2, 2024, and no records were retrieved.


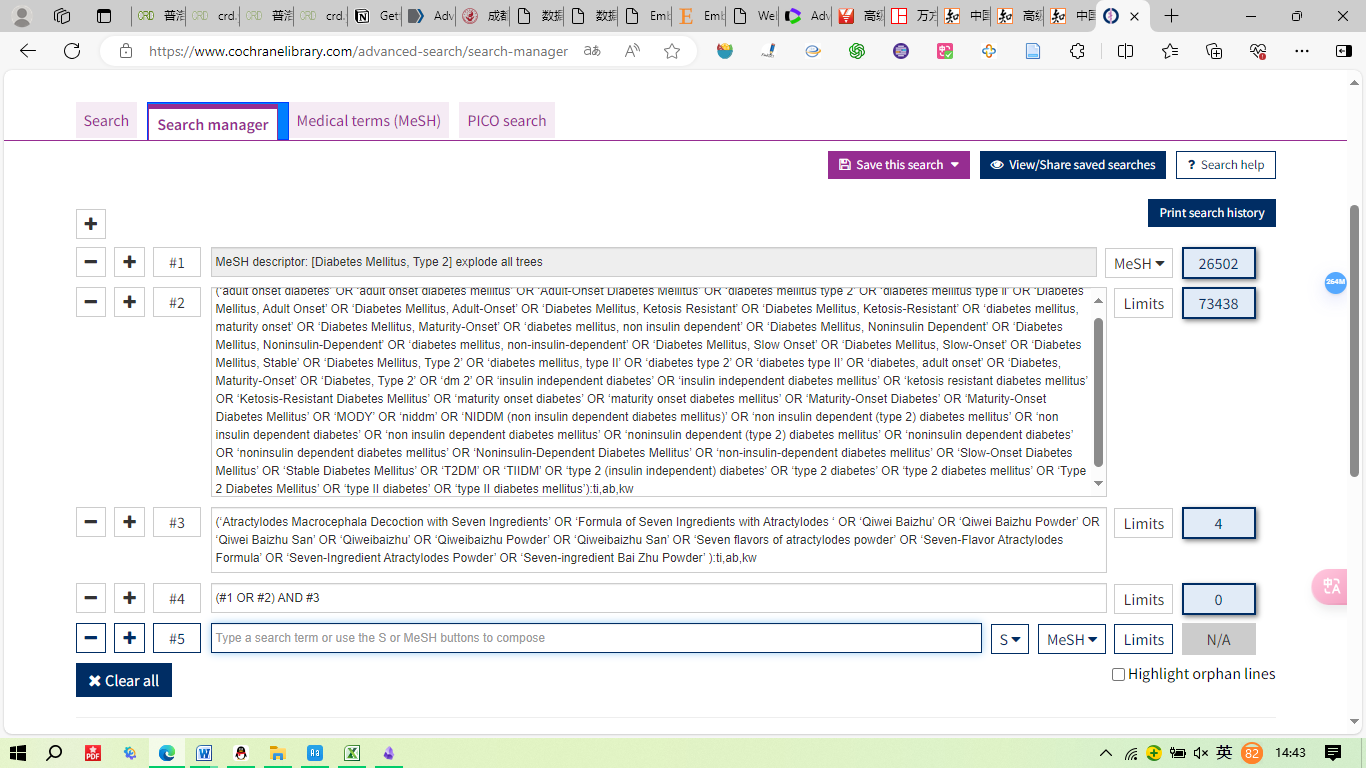


## WOS

The retrieval of the WOS database was conducted on August 2, 2024, and no records were retrieved.

| History | Search Query | Results |
| --- | --- | --- |
| #3 | #1 AND #2 | 0 |
| #2 | TS=((“Atractylodes Macrocephala Decoction with Seven Ingredients”) OR (“Formula of Seven Ingredients with Atractylodes “) OR (“Qiwei Baizhu”) OR (“Qiwei Baizhu Powder”) OR (“Qiwei Baizhu San”) OR (“Qiweibaizhu”) OR (“Qiweibaizhu Powder”) OR (“Qiweibaizhu San”) OR (“Seven flavors of atractylodes powder”) OR (“Seven-Flavor Atractylodes Formula”) OR (“Seven-Ingredient Atractylodes Powder”) OR (“Seven-ingredient Bai Zhu Powder”)) | 12 |
| #1 | TS=((“adult onset diabetes”) OR (“adult onset diabetes mellitus”) OR (“Adult-Onset Diabetes Mellitus”) OR (“diabetes mellitus type 2”) OR (“diabetes mellitus type ii”) OR (“Diabetes Mellitus, Adult Onset”) OR (“Diabetes Mellitus, Adult-Onset”) OR (“Diabetes Mellitus, Ketosis Resistant”) OR (“Diabetes Mellitus, Ketosis-Resistant”) OR (“diabetes mellitus, maturity onset”) OR (“Diabetes Mellitus, Maturity-Onset”) OR (“diabetes mellitus, non insulin dependent”) OR (“Diabetes Mellitus, Noninsulin Dependent”) OR (“Diabetes Mellitus, Noninsulin-Dependent”) OR (“diabetes mellitus, non-insulin-dependent”) OR (“Diabetes Mellitus, Slow Onset”) OR (“Diabetes Mellitus, Slow-Onset”) OR (“Diabetes Mellitus, Stable”) OR (“Diabetes Mellitus, Type 2”) OR (“diabetes mellitus, type II”) OR (“diabetes type 2”) OR (“diabetes type II”) OR (“diabetes, adult onset”) OR (“Diabetes, Maturity-Onset”) OR (“Diabetes, Type 2”) OR (“dm 2”) OR (“insulin independent diabetes”) OR (“insulin independent diabetes mellitus”) OR (“ketosis resistant diabetes mellitus”) OR (“Ketosis-Resistant Diabetes Mellitus”) OR (“maturity onset diabetes”) OR (“maturity onset diabetes mellitus”) OR (“Maturity-Onset Diabetes”) OR (“Maturity-Onset Diabetes Mellitus”) OR (“MODY”) OR (“niddm”) OR (“NIDDM (non insulin dependent diabetes mellitus)”) OR (“non insulin dependent (type 2) diabetes mellitus”) OR (“non insulin dependent diabetes”) OR (“non insulin dependent diabetes mellitus”) OR (“noninsulin dependent (type 2) diabetes mellitus”) OR (“noninsulin dependent diabetes”) OR (“noninsulin dependent diabetes mellitus”) OR (“Noninsulin-Dependent Diabetes Mellitus”) OR (“non-insulin-dependent diabetes mellitus”) OR (“Slow-Onset Diabetes Mellitus”) OR (“Stable Diabetes Mellitus”) OR (“T2DM”) OR (“TIIDM”) OR (“type 2 (insulin independent) diabetes”) OR (“type 2 diabetes”) OR (“type 2 diabetes mellitus”) OR (“Type 2 Diabetes Mellitus”) OR (“type II diabetes”) OR (“type II diabetes mellitus”)) | 165878 |

## CNKI

The retrieval of the CNKI database was conducted on August 2, 2024, and a total of 95 records were retrieved.

(SU=’七味白术’+’七味白术散’+’七味白术方’ OR TKA=’七味白术’+’七味白术散’+’七味白术方’) AND (SU=’糖尿病’+’2型糖尿病’+’二型糖尿病’+’Ⅱ型糖尿病’+’消渴’+’消瘅’+’T2DM’+’DM’ OR TKA=’糖尿病’+’2型糖尿病’+’二型糖尿病’+’Ⅱ型糖尿病’+’消渴’+’消瘅’+’T2DM’+’DM’)


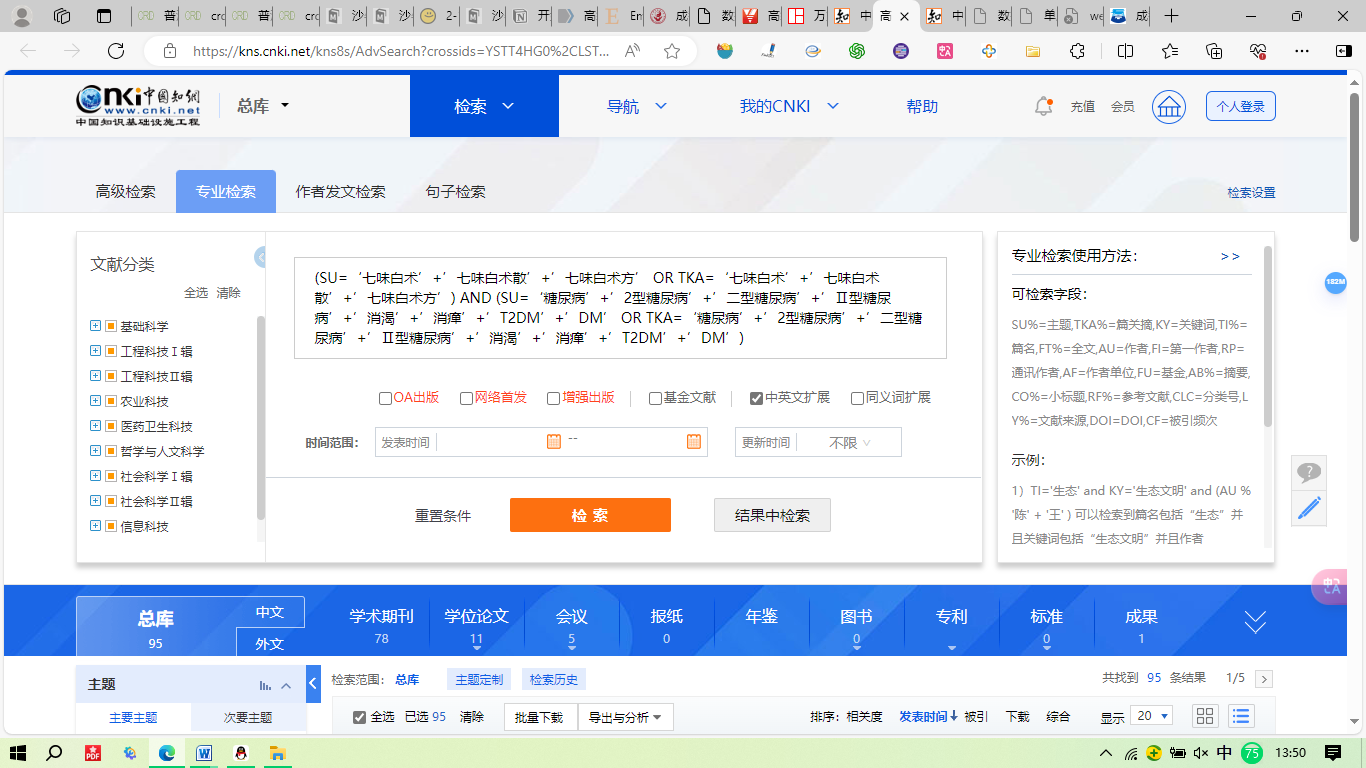


## Wan Fang

The retrieval of the Wan Fang database was conducted on August 2, 2024, and a total of 149 records were retrieved.

(主题:(七味白术 or 七味白术散 or 七味白术方) or 题名或关键词:(七味白术 or 七味白术散 or 七味白术方)) and (主题:( 糖尿病 or 2型糖尿病 or 二型糖尿病 or Ⅱ型糖尿病 or 消渴 or 消瘅 or T2DM or DM) or 题名或关键词:( 糖尿病 or 2型糖尿病 or 二型糖尿病 or Ⅱ型糖尿病 or 消渴 or 消瘅 or T2DM or DM))


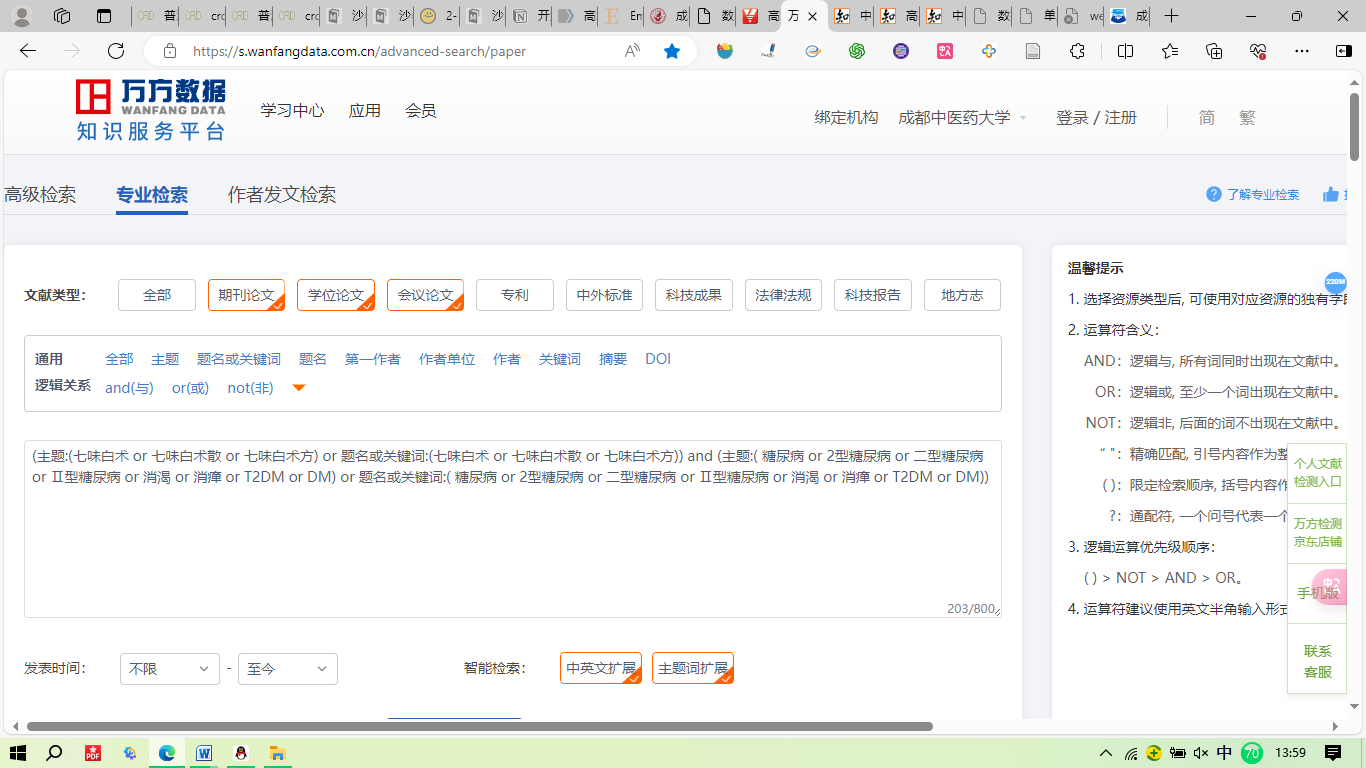


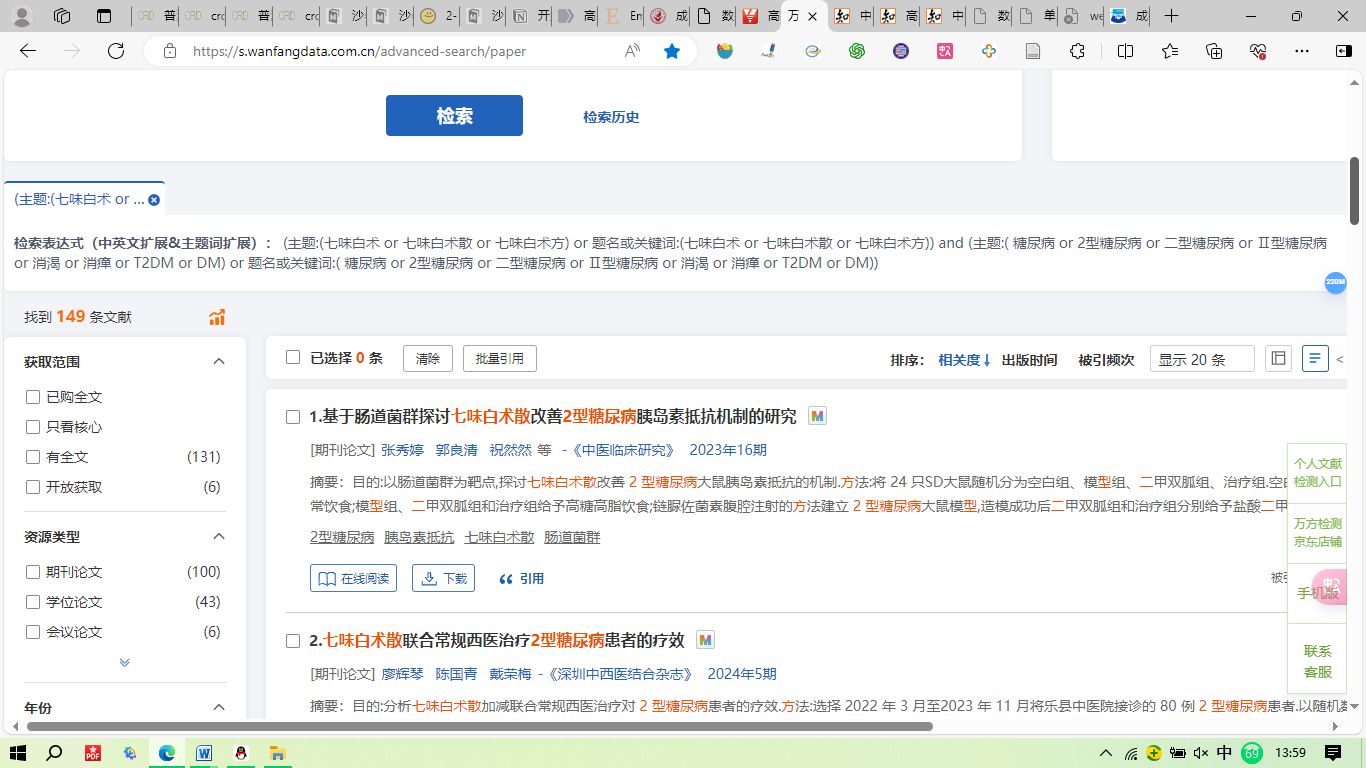


## VIP

The retrieval of the VIP database was conducted on August 2, 2024, and a total of 89 records were retrieved.

((M=(七味白术+七味白术散+七味白术方)) OR (R=(七味白术+七味白术散+七味白术方))) AND ((M=(糖尿病+2型糖尿病+二型糖尿病+Ⅱ型糖尿病+消渴+消瘅+T2DM+DM)) OR (R=(糖尿病+2型糖尿病+二型糖尿病+Ⅱ型糖尿病+消渴+消瘅+T2DM+DM)))
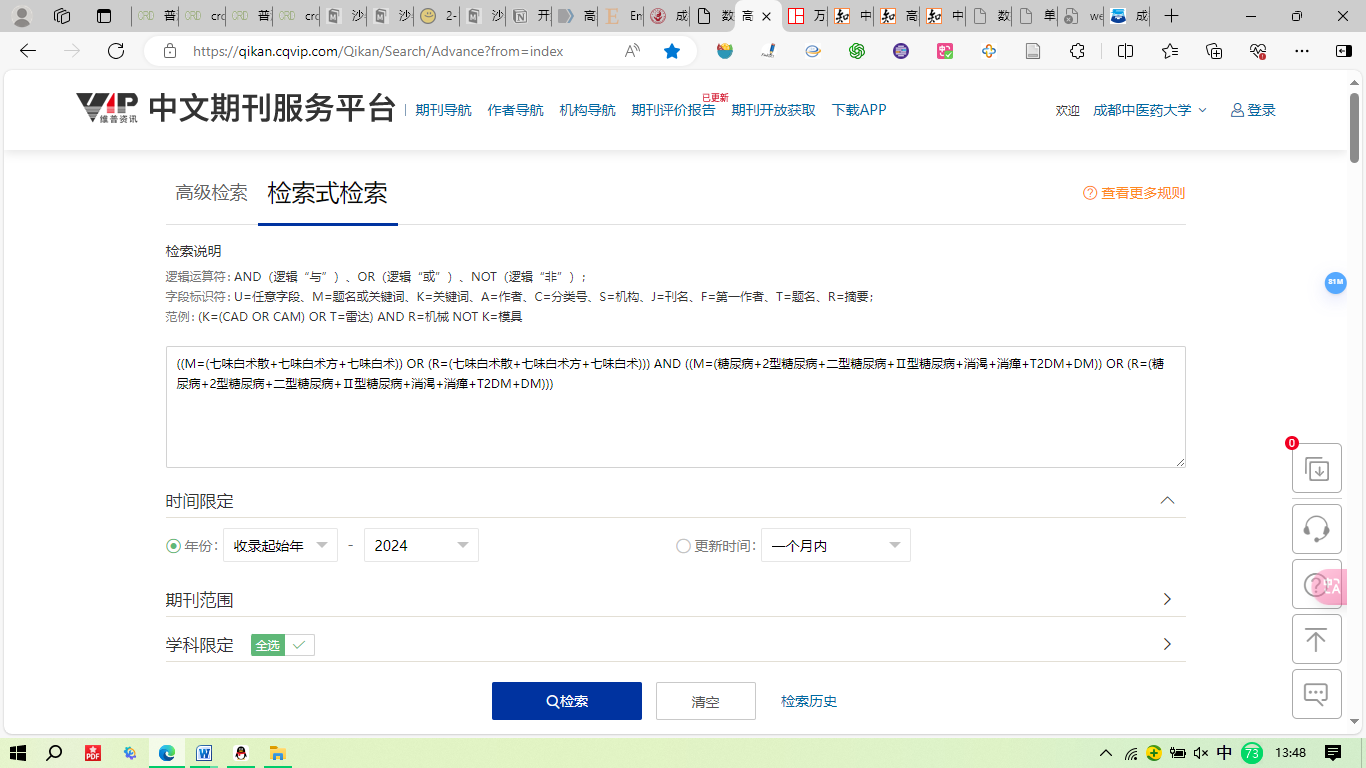

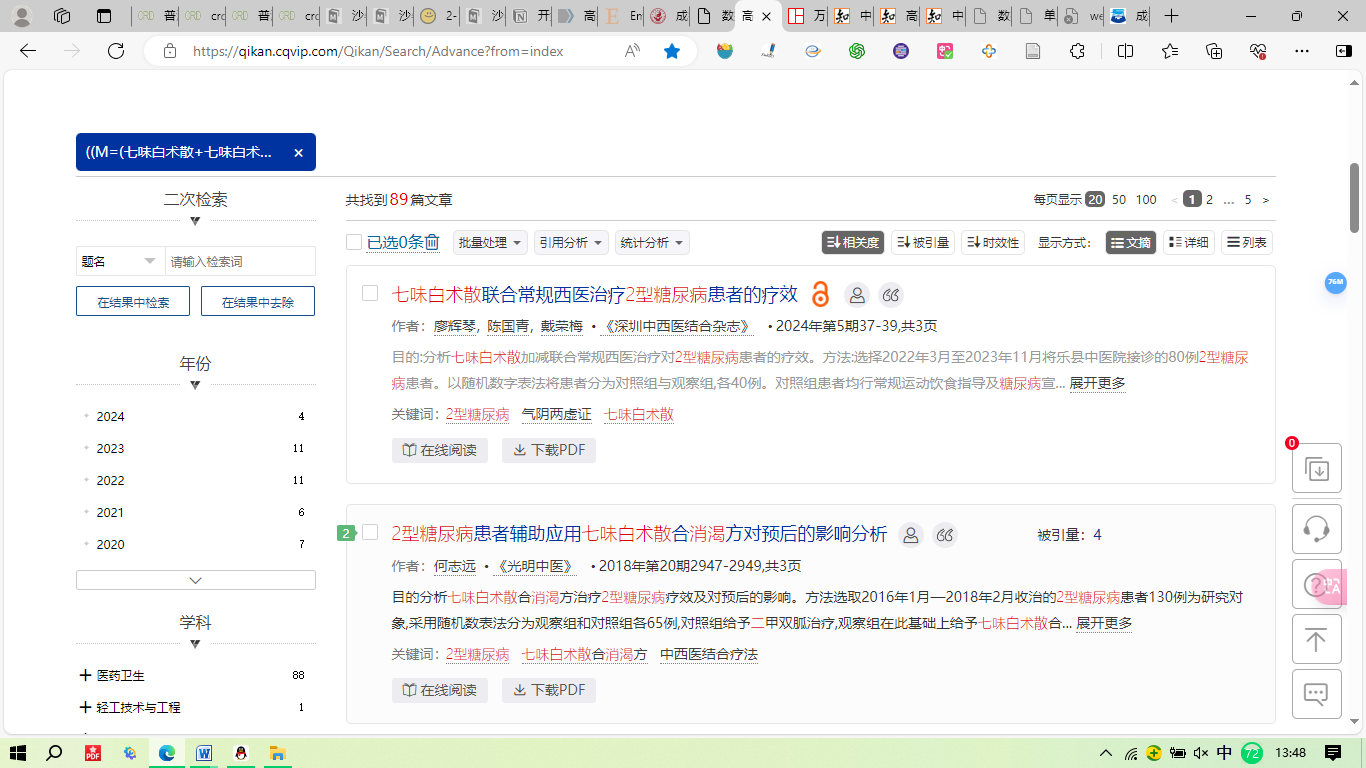


## CBM

The retrieval of the CBM database was conducted on August 2, 2024, and a total of 80 records were retrieved.

((“糖尿病, 2型”[不加权:扩展]) OR (“糖尿病”[常用字段:智能] OR “2型糖尿病”[常用字段:智能] OR “二型糖尿病”[常用字段:智能] OR “Ⅱ型糖尿病”[常用字段:智能] OR “消渴”[常用字段:智能] OR “消瘅”[常用字段:智能] OR “T2DM”[常用字段:智能] OR “DM”[常用字段:智能])) AND ((“七味白术散”[不加权:扩展]) OR (“七味白术”[常用字段:智能] OR “七味白术散”[常用字段:智能] OR “七味白术方”[常用字段:智能]))


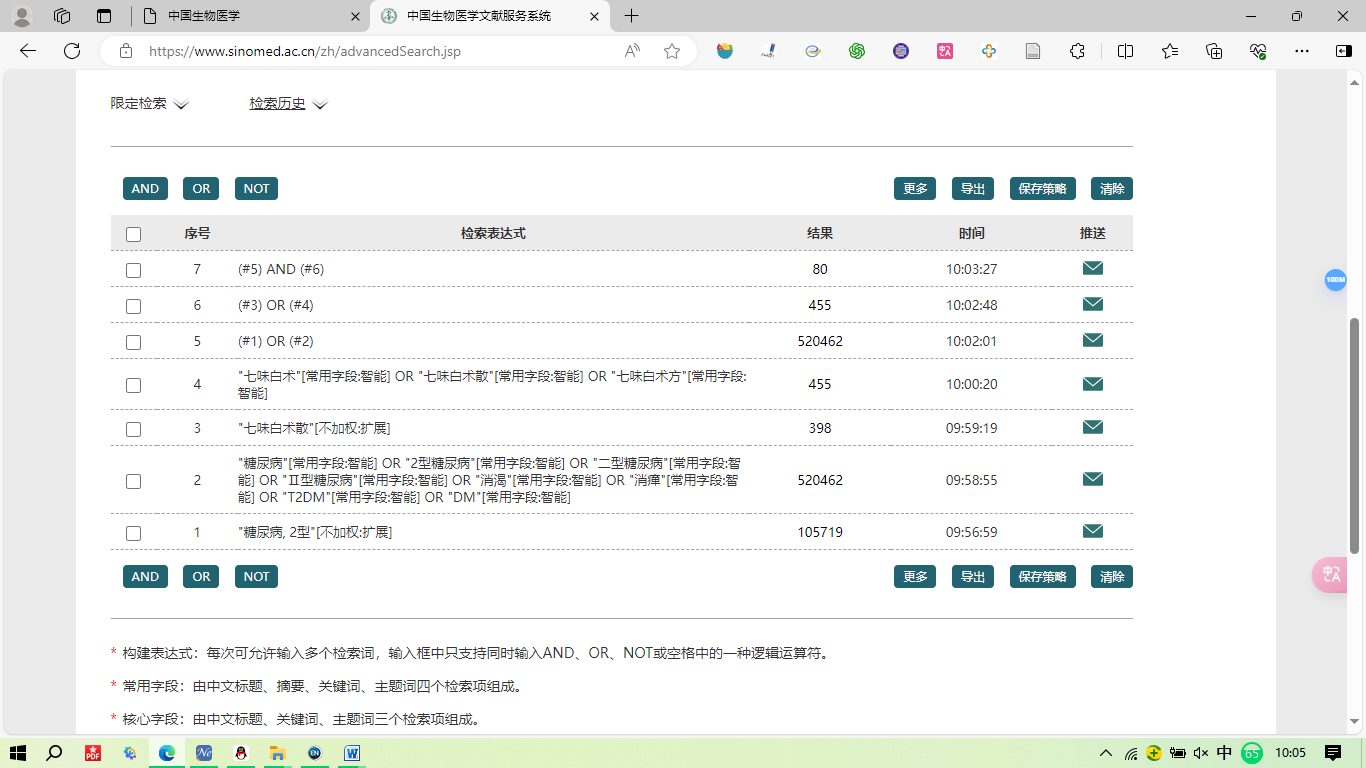


## ClinicalTrials.gov

The retrieval of the ClinicalTrials.gov database was conducted on August 2, 2024, and no records were retrieved.


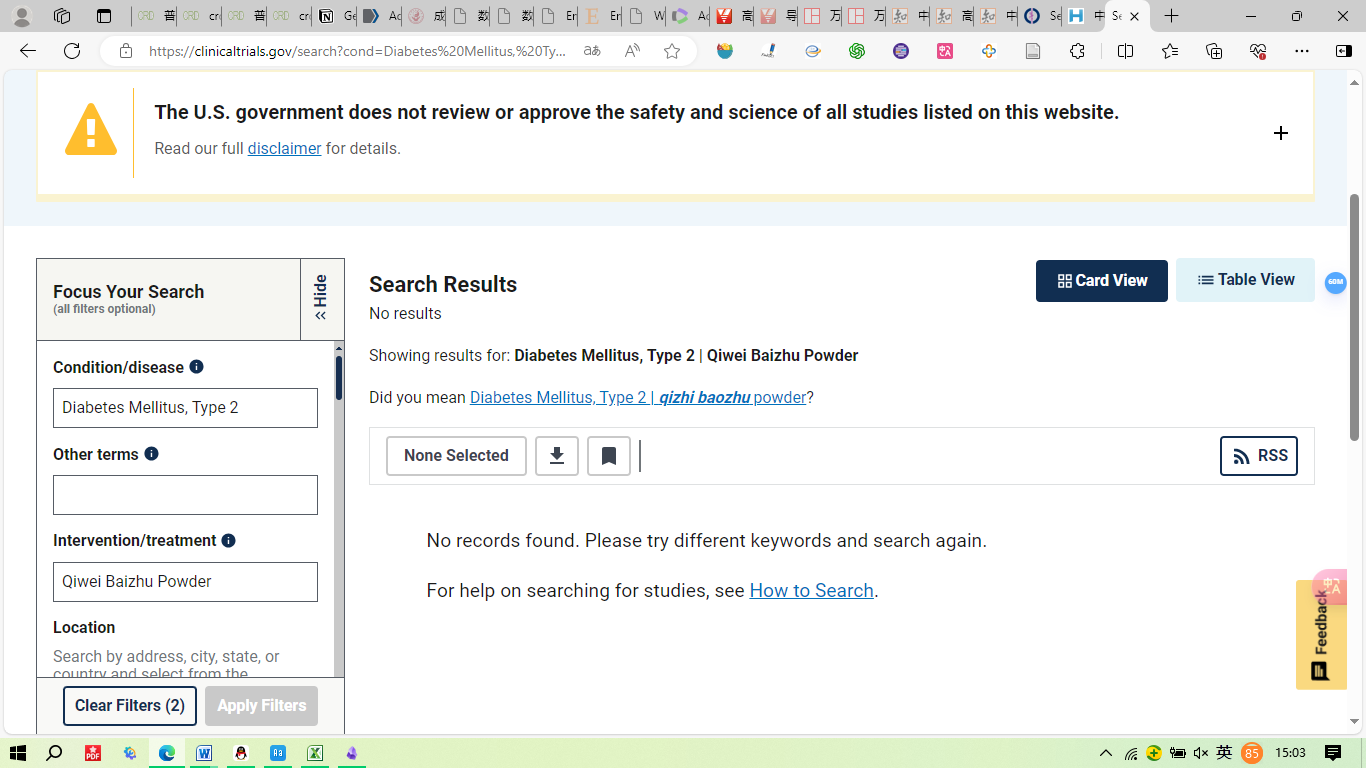


## Chinese Clinical Trial Registry

The retrieval of the Chinese Clinical Trial Registry was conducted on August 2, 2024, and no records were retrieved.
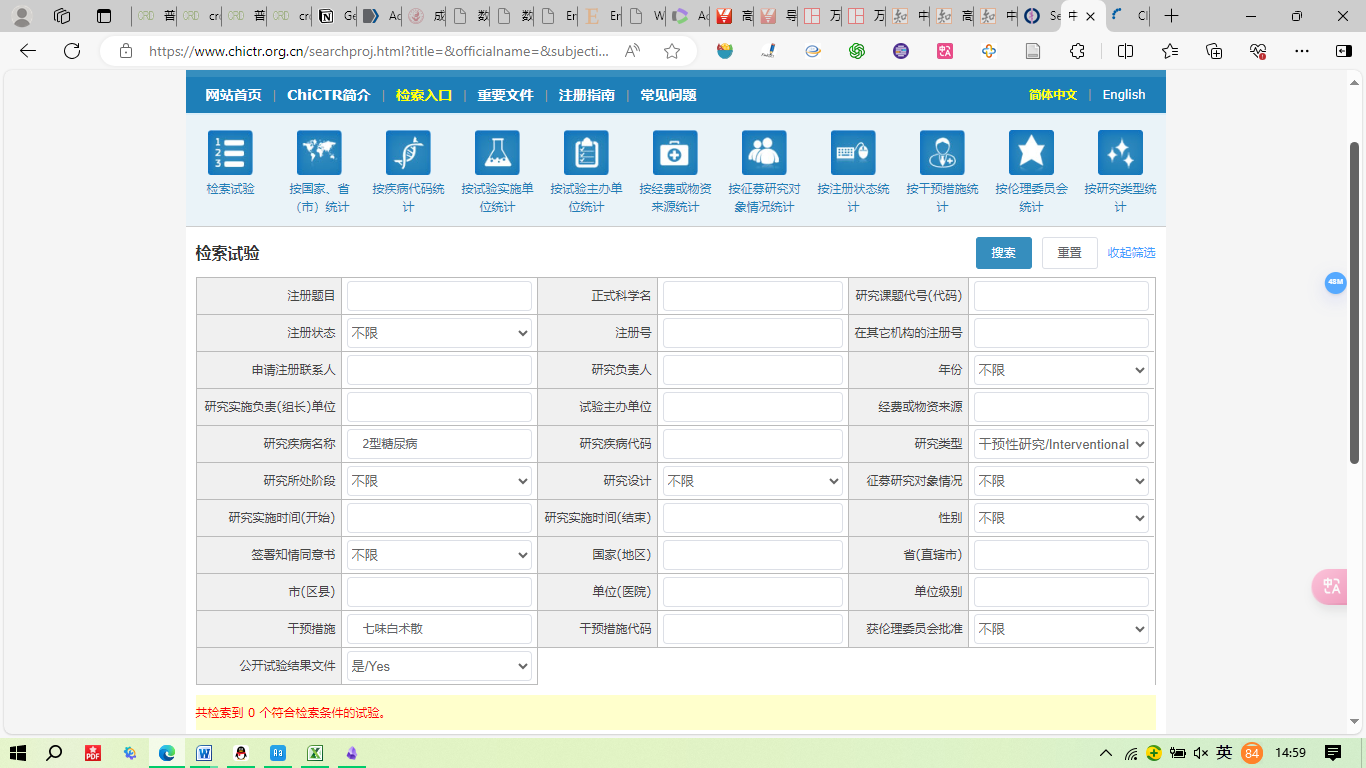


# Supplementary Material S3. The procedure for preliminary screening of the literature
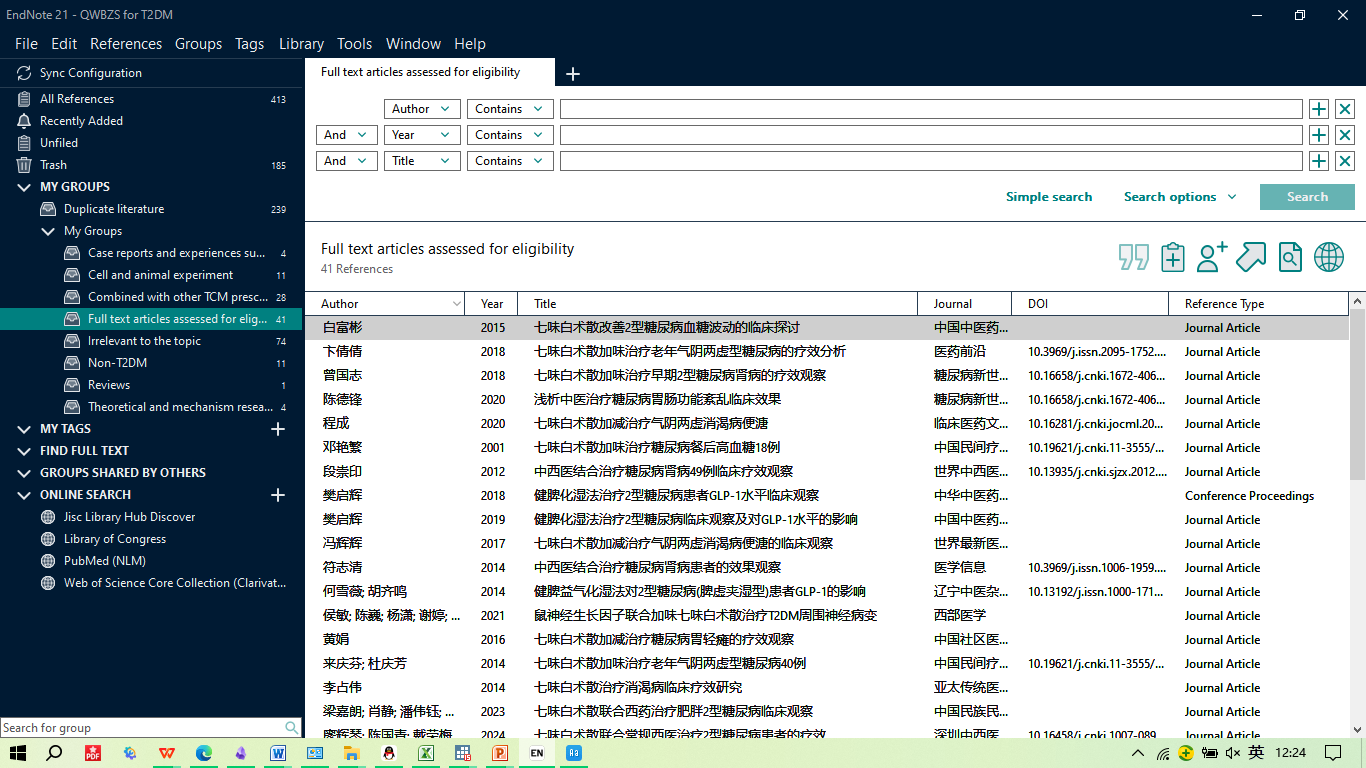


# Supplementary Material S4. Literature excluded after reading the full text and reasons

## The diagnosis in these studies was unclear or non-type 2 diabetes

[1] Bian, Q.Q. (2018). Analysis of the efficacy of modified qiwei baizhu san in the treatment of elderly diabetes mellitus with qi and yin deficiency. Frontiers in Medicine 8(1), 312-313. doi: 10.3969/j.issn.2095-1752.2018.01.269.

[2] Cheng, C.(2020). Qiwei Baizhu Powder for treating loose stool caused by deficiency of Qi-yin and Wasting and thirst disorders. Clinical medicine literature electron magazine 7 (53), 145-146. The doi: 10.16281 / j.carol carroll nki jocml. 2020.53.100.

[3] Deng, Y.F. (2001). Treatment of 18 cases of diabetic postprandial hyperglycemia with added flavor of qiwei baizhu powder. Chinese Folk Therapy (02), 40-41. doi: 10.19621/j.cnki.11-3555/r.2001.02.050.

[4] Feng, H. H. (2017). Clinical observation of modified qiwei baizhu powder in treating loose stool caused by deficiency of qi-yin and wasting and thirst disorders. World Latest Medical Information Digest 17(40), 162-163.

[5] Huang, J. (2016). Observation on the therapeutic effect of modified qiwei baizhu powder in the treatment of diabetic gastroparesis. Chinese Community Physician 32(20), 97-98.

[6] Lu, L., and Xu, H. (2014). Treatment of diabetic gastrointestinal dysfunction in 30 cases with addition and subtraction of modified qiwei baizhu powder. Henan Traditional Chinese Medicine 34(11), 2190-2191. doi: 10.16367/j.issn.1003-5028.2014.11.039.

[7] Ma, M.J. (2014). Clinical observation on the treatment of diabetic diarrhea by modified qiwei baizhu powder. Guangxi Traditional Chinese Medicine 37(03), 12-14.

[8] Pan, Y.K. (2021). Study on the effect of adding and subtracting seven-flavored white atractylodes drink in the treatment of qi and yin deficiency-type thirst-quenching disease. World Digest of Recent Medical Information 21(62), 219-220. doi: 10.3969/j.issn.1671-3141.2021.62.088.

[9] Su, H.L. (2017). Observations on the efficacy of addition and subtraction of modified qiwei baizhu powder with western medicine in treating diabetic nephropathy in 27 cases. National Medical Forum 32(04), 42-43. doi: 10.13913/j.cnki.41-1110/r.2017.04.021.

[10] Wang, Q.X., Zheng, X.J., and He, Z.Z. (2011). Treatment of diabetic enteropathy in 58 cases with qiwei baizhu powder. Massage and Rehabilitation Medicine (in Chinese) (3), 194.

[11] Zhang, J.D., Chen, J., Zhao, X. Ding, KK., Hao H.B., and Zou, Y. (2006). Clinical observation on 48 cases of diabetes mellitus treated with combination of Chinese and Western medicine. Journal of Practical Chinese Medicine 20(6), 627-628. doi: 10.3969/j.issn.1671-7813.2006.06.052.

[12] Zhang, M.D. (1997). Treatment of 29 cases of deficiency-cold diabetic diarrhea with qiwei baizhu powder. Journal of Practical Chinese Medicine (03), 15.

[13] Zhang, M.Q., Pi, Y., and Xie, Y.Q. (2015). Clinical study on the treatment of diabetic diarrhea with qiwei baizhu powder. Medical Information (31), 284-284,285. doi: 10.3969/j.issn.1006-1959.2015.31.424.

## The intervention in these studies was not QWBZS or was combined with other TCM therapies

[1] Chen, D. F. (2020). Clinical effects of chinese medicine in the treatment of diabetic gastrointestinal dysfunction. Diabetes New World 23(14), 58-60. doi: 10.16658/j.cnki.1672-4062.2020.14.058.

[2] Hou, M., Chen, W., Yang, X., Xie, T. and Guo, X.S. (2021). Treatment of T2DM peripheral neuropathy with murine nerve growth factor in combination with added flavor of qiwei baizhu powder. Western Medicine 33(10), 1486-1491.

## The study design was not a randomized controlled trial

[1] Bai, F.B. (2015). Clinical discussion on improvement of blood glucose fluctuation in type 2 diabetes mellitus by qiwei baizhu powder. Modern Distance Education of Chinese Traditional Medicine 13(20), 137-138.

[2] Lai, Q. F., and Du, Q. F. (2014). Treatment of 40 cases of diabetes mellitus with deficiency of qi and yin in the elderly by adding flavor to qiwei baizhu powder. Chinese Folk Therapy 22(02), 36-37. doi: 10.19621/j.cnki.11-3555/r.2014.02.031.

[3] Song, X.Z., Liu, C.H. (2001). Combination of Chinese and Western medicine in the treatment of diabetic diarrhea in 28 cases. Shandong Journal of Traditional Chinese Medicine 20(5).

[4] Liu, F., and Wang, B. (2020). Clinical effect of the addition and subtraction of modified qiwei baizhu powder in the treatment of diabetic nephropathy. Journal of Contemporary Medicine 18(24), 138-139. doi: 10.3969/j.issn.2095-7629.2020.24.095.

[5] Qu, Y.Y., and Liu, S.R.(2016). Self-designed thirst-quenching formula combined with modified qiwei baizhu powder to assist in the treatment of type 2 diabetes mellitus in 39 cases. World Digest of Recent Medical Information 16(52), 170+172.

## Lack of sufficient data results

[1] Sun, D. L. (1996). Treatment of 83 cases of type II diabetes mellitus with modified qiwei baizhu powder. Journal of Guiyang College of Traditional Chinese Medicine (04), 16-17. doi: 10.16588/j.cnki.issn1002-1108.1996.04.009.

[2] Zhang, Y.L., and Wang, H.Z. (2008). Treatment of diabetic diarrhea in 36 cases by adding flavor to qiwei baizhu powder. Shandong Journal of Traditional Chinese Medicine (05), 306-307. doi: 10.16295/j.cnki.0257-358x.2008.05.011.

[3] Zeng, G.Z. (2018). Observation on the efficacy of adding flavor to qiwei baizhu powder in the treatment of early type 2 diabetic nephropathy. Diabetes New World 21(06), 165-166+171. doi: 10.16658/j.cnki.1672-4062.2018.06.165.

[4] Xu, Y., and Ji, J.J. (2021). Qiwei Bai Zhu San combined with metformin for body mass control in diabetes combined with obesity. Scientific Nutrition 24(17), 234. doi: 10.3969/j.issn.1672-9714.2021.17.232.

## Duplicate literature

[1] Fan, Q. H. (2018). Clinical observation of GLP-1 level in patients with type 2 diabetes mellitus treated by strengthening the spleen and resolving dampness method. National Conference on Diabetes in Traditional Chinese Medicine of Diabetes Branch, Chinese Society of Traditional Chinese Medicine (19th), 2.

## Subjects may have severe renal impairment

[1] Duan, C.Y. (2012). Clinical efficacy of combined Chinese and Western medicine in treating diabetic nephropathy in 49 cases. World Journal of Integrative Medicine 7(06), 513-515. doi: 10.13935/j.cnki.sjzx.2012.06.022.

[2] Fu, Z. Q. (2014). Observation on the effect of combined Chinese and Western medicine in treating patients with diabetic nephropathy. Medical Information (12), 418-418. doi: 10.3969/j.issn.1006-1959.2014.12.464.

# Supplementary Material S5. The components of the original QWBZS or modified QWBZS

| Study | Formula | Components |
| --- | --- | --- |
| Fan,2019 | Modified QWBZS | Tangshen (Dangshen, Codonopsis pilosula (Franch.) Nannf.) 15g, Largehead Atractylodes (Baizhu, Atractylodes macrocephala Koidz.) 15g, Indian bread (Fuling, Poria cocos (Schw. ) Wolf.) 15g, Liquorice Root (Gancao, Glycyrrhiza glabra L.) 6g, Common aucklandia root (Muxiang, Aucklandia lappa Decne.) 6g, Wrinkled gianthyssop hearb (Huoxiang, Agastache rugosa (Fisch.etMey.)) 15g, Thomson Kudzuvine root (Gegen, Pueraria montana var. thomsonii (Benth.) M. R. Almeida) 15g. If pathogenic dampness was identified, Fortune’s Eupatorium Herb (Peilan, Eupatorium fortunei Turcz.) 15 g, Rhizoma Atractylodis (Cangzhu, Atractylodes lancea (Thunb.) DC.) 12 g and Corn stigma (Yumixu, Stigma Maydis) 20 g were added; If qi deficiency was identified, Milkvetch Root (Huangqi, Astragalus mongholicus Bunge) 20 g, Common Yam Rhizome (Shanyao, Dioscorea oppositifolia L.) 15 g and Coix Seed (Yiyiren, Coix lacryma-jobi L.) 15 g were added; If liver qi stagnation pattern was identified, Chinese Thorowax Root (Chaihu, Bupleurum falcatum L.) 12 g and Turmeric Root Tuber (Yujin, Curcuma longa L.) 12 g were added; If kidney deficiency pattern was identified, Figwort Root (Xuanshen, Scrophularia ningpoensis Hemsl.) 15 g, Dwarf Lilyturf Tuber (Maidong, Ophiopogon japonicus (Thunb.) Ker Gawl.) 15 g and Barbary Wolfberry Fruit (Gouqizi, Lycium barbarum L.) 15 g were added; If pathogenic heat was identified, Golden thread (Huanglian, Coptis chinensis Franch.) 6 g, Common Gardenia Fruit (Zhizi, Plumeria rubra L.) 10 g and Giant Knotweed Rhizome (Huzhang, Reynoutria japonica Houtt.) 15 g were added. |
| He et al.,2014 | Modified QWBZS | Heterophylly falsestarwort root (Taizishen, Pseudostellaria heterophylla (Miq.) Pax ex Pax et Hoffm.) 15g, Largehead Atractylodes (Baizhu, Atractylodes macrocephala Koidz.) 15g, Indian bread (Fuling, Poria cocos (Schw. ) Wolf.) 15g, Liquorice Root (Gancao, Glycyrrhiza glabra L.) 6g, Common aucklandia root (Muxiang, Aucklandia lappa Decne.) 6g, Wrinkled gianthyssop hearb (Huoxiang, Agastache rugosa (Fisch.etMey.)) 15g, Thomson Kudzuvine root (Gegen, Pueraria montana var. thomsonii (Benth.) M. R. Almeida) 15g. If pathogenic dampness was identified, Fortune’s Eupatorium Herb (Peilan, Eupatorium fortunei Turcz.) 15 g, Rhizoma Atractylodis (Cangzhu, Atractylodes lancea (Thunb.) DC.) 12 g and Corn stigma (Yumixu, Stigma Maydis) 20 g were added; If qi deficiency was identified, Milkvetch Root (Huangqi, Astragalus mongholicus Bunge) 20 g, Common Yam Rhizome (Shanyao, Dioscorea oppositifolia L.) 15 g and Coix Seed (Yiyiren, Coix lacryma-jobi L.) 15 g were added; If liver qi stagnation pattern was identified, Chinese Thorowax Root (Chaihu, Bupleurum falcatum L.) 12 g and Turmeric Root Tuber (Yujin, Curcuma longa L.) 12 g were added; If kidney deficiency pattern was identified, Figwort Root (Xuanshen, Scrophularia ningpoensis Hemsl.) 15 g, Dwarf Lilyturf Tuber (Maidong, Ophiopogon japonicus (Thunb.) Ker Gawl.) 15 g and Barbary Wolfberry Fruit (Gouqizi, Lycium barbarum L.) 15 g were added; If pathogenic heat was identified, Golden thread (Huanglian, Coptis chinensis Franch.) 6 g, Common Gardenia Fruit (Zhizi, Plumeria rubra L.) 10 g and Giant Knotweed Rhizome (Huzhang, Reynoutria japonica Houtt.) 15 g were added. |
| Li,2014 | Modified QWBZS | Tangshen (Dangshen, Codonopsis pilosula (Franch.) Nannf.) 20g, Largehead Atractylodes (Baizhu, Atractylodes macrocephala Koidz.) 15g, Indian bread (Fuling, Poria cocos (Schw. ) Wolf.) 15g, Liquorice Root (Gancao, Glycyrrhiza glabra L.) 6g, Common aucklandia root (Muxiang, Aucklandia lappa Decne.) 6g, Wrinkled gianthyssop hearb (Huoxiang, Agastache rugosa (Fisch.etMey.)) 12g, Thomson Kudzuvine root (Gegen, Pueraria montana var. thomsonii (Benth.) M. R. Almeida) 20g.If the patient is accompanied by severe thirst, Dwarf Lilyturf Tuber (Maidong, Ophiopogon japonicus (Thunb.) Ker Gawl.), Coastal Glehnia Root (Shashen, Glehnia littoralis Fr.SchmidtexMiq.) and Snakegourd Root (Tianhuafen, Trichosanthes kirilowii Maxim.) were added; If severe stomach fire pattern was identified, Gypsum (Shigao, Gypsum Fibrosum), Common Anemarrhena Rhizome (Zhimu, Anemarrhena asphodeloides Bunge) and Dwarf Lilyturf Tuber (Maidong, Ophiopogon japonicus (Thunb.) Ker Gawl.) were added; If lassitude was identified, Milkvetch Root (Huangqi, Astragalus mongholicus Bunge) was added; If the patient is accompanied by a large amount of urine, Chinese Magnoliavine Fruit (Wuweizi, Schisandra chinensis (Turcz.) Baill.) and Sharpleaf Galangal Fruit (Yizhiren, Alpinia oxyphylla Miq.) were added; If blood stasis was identified, Danshen Root (Danshen, Salvia miltiorrhiza Bunge), Tree Peony Bark (Mudanpi, Paeonia × suffruticosa Andrews) and Hirsute Bugleweed Herb (Zelan, Chromolaena odorata (L.) R.M.King & H.Rob.) were added. |
| Liang et al.,2023 | Original QWBZS | Ginseng Root (Renshen, Panax ginseng C. A. Meyer) 6g, Largehead Atractylodes (Baizhu, Atractylodes macrocephala Koidz.) 12g, Indian bread (Fuling, Poria cocos (Schw. ) Wolf.) 12g, Liquorice Root (Gancao, Glycyrrhiza glabra L.) 3g, Common aucklandia root (Muxiang, Aucklandia lappa Decne.) 6g, Wrinkled gianthyssop hearb (Huoxiang, Agastache rugosa (Fisch.etMey.)) 12g, Thomson Kudzuvine root (Gegen, Pueraria montana var. thomsonii (Benth.) M. R. Almeida) 15g. |
| Liao et al.,2024 | Modified QWBZS | Tangshen (Dangshen, Codonopsis pilosula (Franch.) Nannf.) 15g, Largehead Atractylodes (Baizhu, Atractylodes macrocephala Koidz.) 12g, Indian bread (Fuling, Poria cocos (Schw. ) Wolf.) 12g, Honey-broilled liquorice root (Zhigancao, Glycyrrhiza glabra L.) 6g, Common aucklandia root (Muxiang, Aucklandia lappa Decne.) 10g, Wrinkled gianthyssop hearb (Huoxiang, Agastache rugosa (Fisch.etMey.)) 12g, Thomson Kudzuvine root (Gegen, Pueraria montana var. thomsonii (Benth.) M. R. Almeida) 10g. If the patient is accompanied by bloating, Chicken's Gizzard-membrane (Jineijin, Gallus gallus domesticus Brisson) 6 g and Villous Amomum Fruit (Sharen, Amomum villosum Lour.) 6 g were added.; If the patient is accompanied by shortness of breath and excessive sweating, Chinese Magnoliavine Fruit (Wuweizi, Schisandra chinensis (Turcz.) Baill.) 6 g and Asiatic Cornelian Cherry Fruit (Shanzhuyu, Cornus officinalis Siebold & Zucc.) 6 g were added; If the patient is accompanied by thirst, Snakegourd Root (Tianhuafen, Trichosanthes kirilowii Maxim.) 9 g and Rehmannia Root (Dihuang, Rehmannia glutinosa (Gaertn.) DC.) 9 g were added. |
| Lin,2022 | Modified QWBZS | Tangshen (Dangshen, Codonopsis pilosula (Franch.) Nannf.) 20g, Largehead Atractylodes (Baizhu, Atractylodes macrocephala Koidz.) 12g, Indian bread (Fuling, Poria cocos (Schw. ) Wolf.) 12g, Honey-broilled liquorice root (Zhigancao, Glycyrrhiza glabra L.) 3g, Common aucklandia root (Muxiang, Aucklandia lappa Decne.) 10g, Wrinkled gianthyssop hearb (Huoxiang, Agastache rugosa (Fisch.etMey.)) 6g, Thomson Kudzuvine root (Gegen, Pueraria montana var. thomsonii (Benth.) M. R. Almeida) 20g. If the patient has a lack of appetite and abdominal distension, Chicken's Gizzard-membrane (Jineijin, Gallus gallus domesticus Brisson) 6 g and Villous Amomum Fruit (Sharen, Amomum villosum Lour.) 9 g were added.; If the patient is accompanied by shortness of breath and excessive sweating, Chinese Magnoliavine Fruit (Wuweizi, Schisandra chinensis (Turcz.) Baill.) 6 g and Asiatic Cornelian Cherry Fruit (Shanzhuyu, Cornus officinalis Siebold & Zucc.) 6 g were added; If the patient is accompanied by thirst, Snakegourd Root (Tianhuafen, Trichosanthes kirilowii Maxim.) 6 g and Rehmannia Root (Dihuang, Rehmannia glutinosa (Gaertn.) DC.) 9 g were added. |
| Liu,2020 | Original QWBZS | Tangshen (Dangshen, Codonopsis pilosula (Franch.) Nannf.) 15g, Largehead Atractylodes (Baizhu, Atractylodes macrocephala Koidz.) 15g, Indian bread (Fuling, Poria cocos (Schw. ) Wolf.) 30g, Liquorice Root (Gancao, Glycyrrhiza glabra L.) 10g, Common aucklandia root (Muxiang, Aucklandia lappa Decne.) 10g, Wrinkled gianthyssop hearb (Huoxiang, Agastache rugosa (Fisch.etMey.)) 10g, Thomson Kudzuvine root (Gegen, Pueraria montana var. thomsonii (Benth.) M. R. Almeida) 30g. |
| Luo,2024 | Modified QWBZS | Tangshen (Dangshen, Codonopsis pilosula (Franch.) Nannf.) 20g, Largehead Atractylodes (Baizhu, Atractylodes macrocephala Koidz.) 12g, Indian bread (Fuling, Poria cocos (Schw. ) Wolf.) 12g, Honey-broilled liquorice root (Zhigancao, Glycyrrhiza glabra L.) 3g, Wrinkled gianthyssop hearb (Huoxiang, Agastache rugosa (Fisch.etMey.)) 6g, Thomson Kudzuvine root (Gegen, Pueraria montana var. thomsonii (Benth.) M. R. Almeida) 20g. If the patient has a lack of appetite and abdominal distension, Villous Amomum Fruit (Sharen, Amomum villosum Lour.) 9 g and Chicken's Gizzard-membrane (Jineijin, Gallus gallus domesticus Brisson) 6 g were added.; If the patient is accompanied by shortness of breath and excessive sweating, Chinese Magnoliavine Fruit (Wuweizi, Schisandra chinensis (Turcz.) Baill.) 6 g and Asiatic Cornelian Cherry Fruit (Shanzhuyu, Cornus officinalis Siebold & Zucc.) 6 g were added; If the patient is accompanied by significant thirst, Rehmannia Root (Dihuang, Rehmannia glutinosa (Gaertn.) DC.) 9 g and Snakegourd Root (Tianhuafen, Trichosanthes kirilowii Maxim.) 6 g were added. |
| Meng et al.,2022 | Modified QWBZS | Ginseng Root (Renshen, Panax ginseng C. A. Meyer) 9g, Largehead Atractylodes (Baizhu, Atractylodes macrocephala Koidz.) 15g, Indian bread (Fuling, Poria cocos (Schw. ) Wolf.) 15g, Honey-broilled liquorice root (Zhigancao, Glycyrrhiza glabra L.) 6g, Common aucklandia root (Muxiang, Aucklandia lappa Decne.) 12g, Cablin potchouli herb (Guanghuoxiang, Pogostemon cablin (Blanco) Benth.) 6g, Thomson Kudzuvine root (Gegen, Pueraria montana var. thomsonii (Benth.) M. R. Almeida) 12g. If the patient is accompanied by significant thirst, Rehmannia Root (Dihuang, Rehmannia glutinosa (Gaertn.) DC.) 12 g and Snakegourd Root (Tianhuafen, Trichosanthes kirilowii Maxim.) 9 g were added; If the patient is accompanied by shortness of breath and excessive sweating, Milkvetch Root (Huangqi, Astragalus mongholicus Bunge) 12 g and Chinese Magnoliavine Fruit (Wuweizi, Schisandra chinensis (Turcz.) Baill.) 6 g were added; If the patient has a lack of appetite and abdominal distension, Villous Amomum Fruit (Sharen, Amomum villosum Lour.) 9 g and Hawthorn Fruit (Shanzha, Crataegus monogyna Jacq) 9 g were added. |
| Niu,2014 | Modified QWBZS | Ginseng Root (Renshen, Panax ginseng C. A. Meyer) 25g, Largehead Atractylodes (Baizhu, Atractylodes macrocephala Koidz.) 20g, Indian bread (Fuling, Poria cocos (Schw. ) Wolf.) 20g, Liquorice Root (Gancao, Glycyrrhiza glabra L.) 6g, Common aucklandia root (Muxiang, Aucklandia lappa Decne.) 15g, Wrinkled gianthyssop hearb (Huoxiang, Agastache rugosa (Fisch.etMey.)) 20g, Thomson Kudzuvine root (Gegen, Pueraria montana var. thomsonii (Benth.) M. R. Almeida) 18g. If excess heat in the lung pattern was identified, Tabasheer (Tianzhuhuang, Bambusa textilis McClure) 10 g, Clam Shell (Haigeke, Meretrix meretrix Linnaeus) 12 g and so on were added; If the patient is accompanied by significant thirst, Turtle Carapace (Biejia, Trionycis Carapax) 20 g, Solomonseal Rhizome (Huangjing, Vitex negundo L.) 15 g and so on were added; If the patient is accompanied by shortness of breath and excessive sweating, blighted wheat (Fuxiaomai, Triticum aestivum L.) 20 g, glutinous rice root (Nuodaogen, Oryza sativa L.var.glutinosa Matsum.) 15 g and so on were added; If the patient is accompanied by significant poor appetite, Rhizoma Atractylodis (Cangzhu, Atractylodes lancea (Thunb.) DC.) 15 g, Cynanchum auriculatum Royle (Geshanxiao, Cynanchum wilfordii (Maxim.) Hook. F) 15 g and so on were added. |
| Qian,2022 | Modified QWBZS | Tangshen (Dangshen, Codonopsis pilosula (Franch.) Nannf.) 15g, Largehead Atractylodes (Baizhu, Atractylodes macrocephala Koidz.) 15g, Indian bread (Fuling, Poria cocos (Schw. ) Wolf.) 15g, Honey-broilled liquorice root (Zhigancao, Glycyrrhiza glabra L.) 3g, Common aucklandia root (Muxiang, Aucklandia lappa Decne.) 6g, Wrinkled gianthyssop hearb (Huoxiang, Agastache rugosa (Fisch.etMey.)) 6g, Thomson Kudzuvine root (Gegen, Pueraria montana var. thomsonii (Benth.) M. R. Almeida) 30g, Tree Peony Bark (Mudanpi, Paeonia × suffruticosa Andrews) 12 g, Golden thread (Huanglian, Coptis chinensis Franch.) 6 g, Dried Ginger (Ganjiang, Zingiber officinale Roscoe) 3 g. |
| Wang,2022 | Modified QWBZS | Heterophylly falsestarwort root (Taizishen, Pseudostellaria heterophylla (Miq.) Pax ex Pax et Hoffm.) 15g, Largehead Atractylodes (Baizhu, Atractylodes macrocephala Koidz.) 15g, Indian bread (Fuling, Poria cocos (Schw. ) Wolf.) 15g, Liquorice Root (Gancao, Glycyrrhiza glabra L.) 6g, Common aucklandia root (Muxiang, Aucklandia lappa Decne.) 6g, Thomson Kudzuvine root (Gegen, Pueraria montana var. thomsonii (Benth.) M. R. Almeida) 10g, Fortune’s Eupatorium Herb (Peilan, Eupatorium fortunei Turcz.) 45g. |
| Wang,2023 | Original QWBZS | Tangshen (Dangshen, Codonopsis pilosula (Franch.) Nannf.) 15g, Largehead Atractylodes (Baizhu, Atractylodes macrocephala Koidz.) 15g, Indian bread (Fuling, Poria cocos (Schw. ) Wolf.) 15g, Honey-broilled liquorice root (Zhigancao, Glycyrrhiza glabra L.) 3g, Common aucklandia root (Muxiang, Aucklandia lappa Decne.) 6g, Wrinkled gianthyssop hearb (Huoxiang, Agastache rugosa (Fisch.etMey.)) 15g, Thomson Kudzuvine root (Gegen, Pueraria montana var. thomsonii (Benth.) M. R. Almeida) 30g. |
| Yang,2023 | Modified QWBZS | Tangshen (Dangshen, Codonopsis pilosula (Franch.) Nannf.) 20g, Largehead Atractylodes (Baizhu, Atractylodes macrocephala Koidz.) 15g, Indian bread (Fuling, Poria cocos (Schw. ) Wolf.) 12g, Liquorice Root (Gancao, Glycyrrhiza glabra L.) 6g, Common aucklandia root (Muxiang, Aucklandia lappa Decne.) 6g, Wrinkled gianthyssop hearb (Huoxiang, Agastache rugosa (Fisch.etMey.)) 9g, Thomson Kudzuvine root (Gegen, Pueraria montana var. thomsonii (Benth.) M. R. Almeida) 30g, Milkvetch Root (Huangqi, Astragalus mongholicus Bunge) 20 g, Common Yam Rhizome (Shanyao, Dioscorea oppositifolia L.) 12 g, Dwarf Lilyturf Tuber (Maidong, Ophiopogon japonicus (Thunb.) Ker Gawl.) 10 g. If the patient is accompanied by significant thirst, Rehmannia Root (Dihuang, Rehmannia glutinosa (Gaertn.) DC.), Snakegourd Root (Tianhuafen, Trichosanthes kirilowii Maxim.) and so on were added; If the patient is accompanied by shortness of breath and excessive sweating, Asiatic Cornelian Cherry Fruit (Shanzhuyu, Cornus officinalis Siebold & Zucc.), Chinese Magnoliavine Fruit (Wuweizi, Schisandra chinensis (Turcz.) Baill.) and so on were added. |

# Supplementary Material S6. Subgroup analysis

## Subgroup analysis of FBG for QWBZS combined with conventional treatment vs. conventional treatment


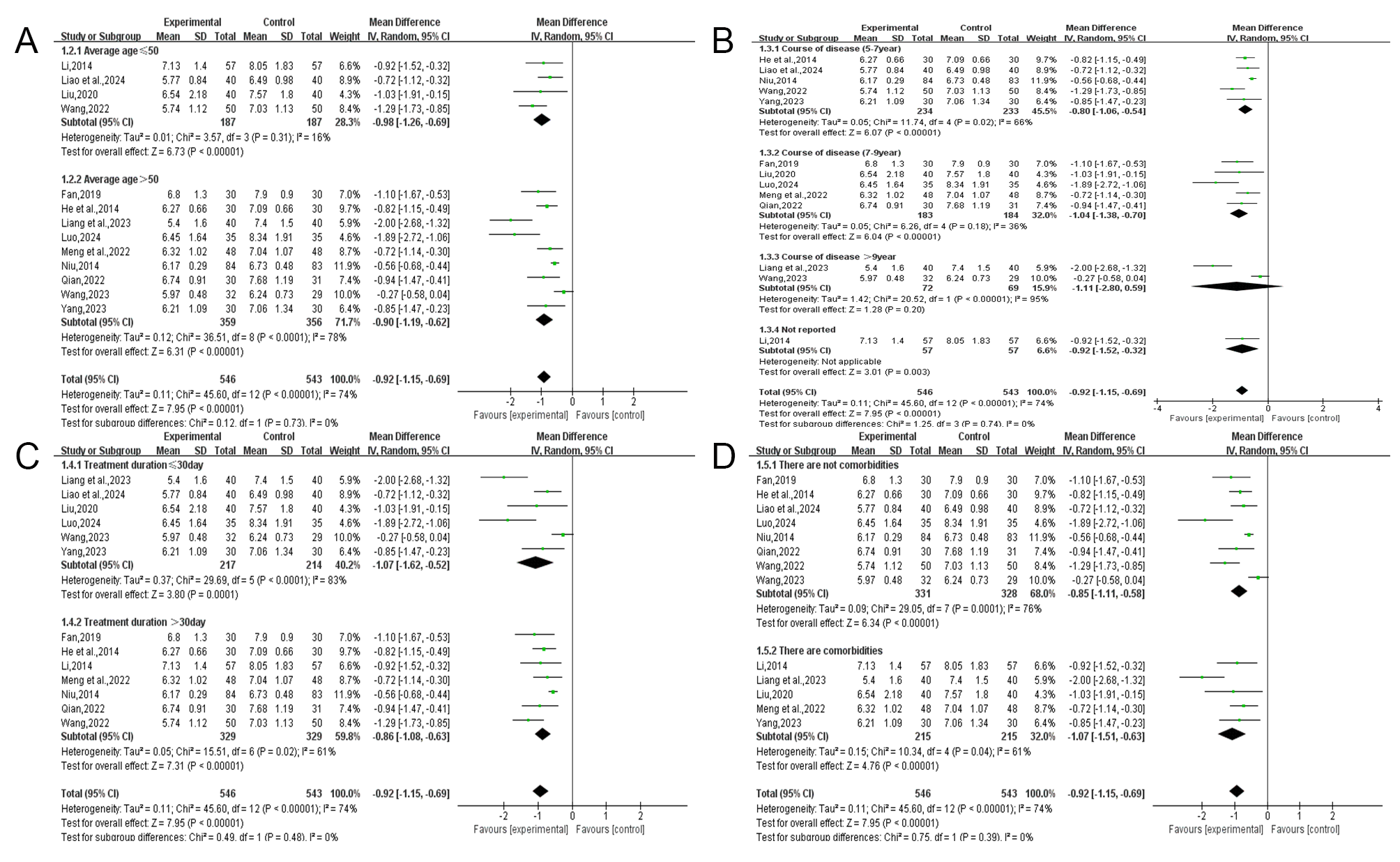


## Subgroup analysis of 2hPG for QWBZS combined with conventional treatment vs. conventional treatment


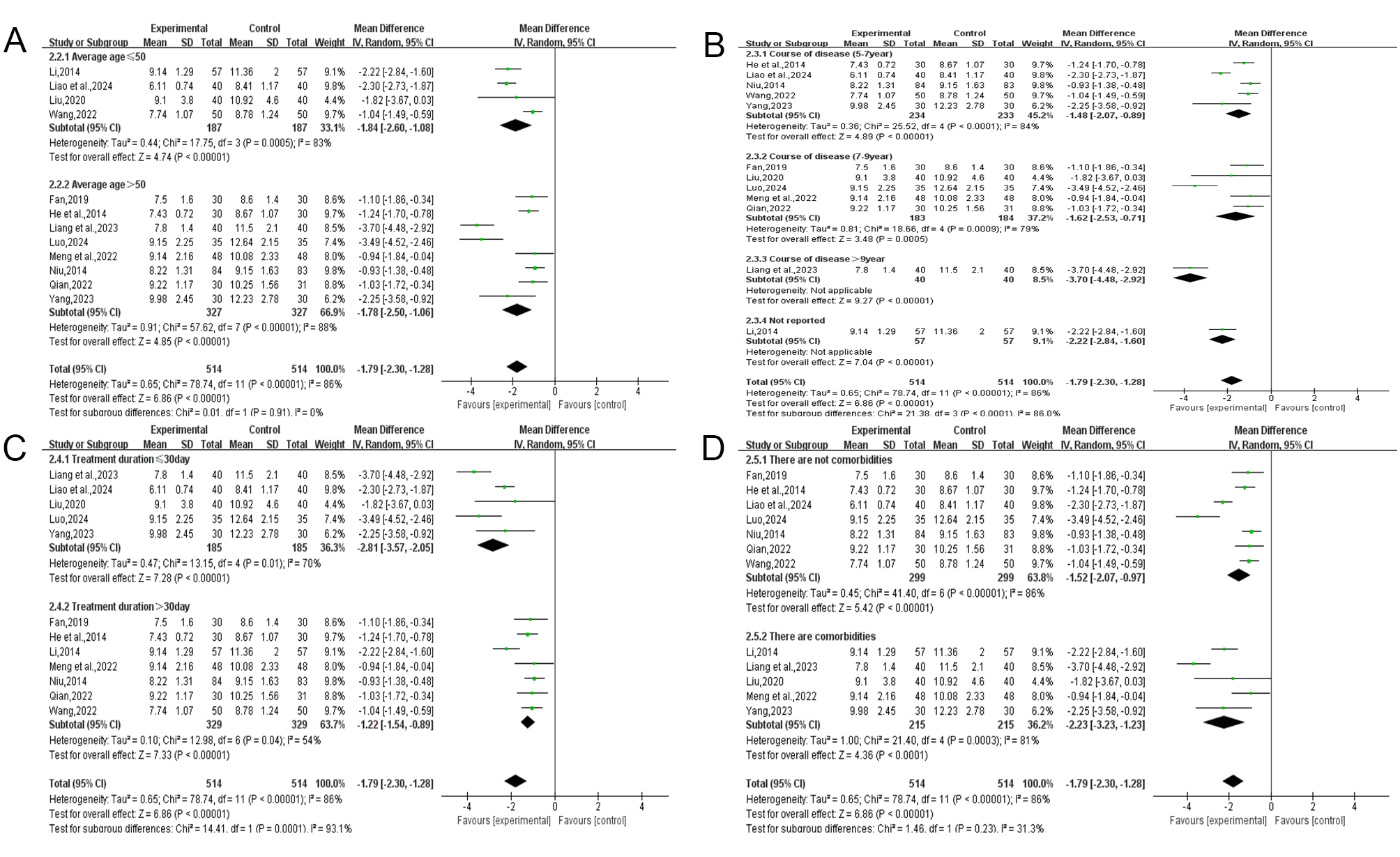


## Subgroup analysis of HbA1c for QWBZS combined with conventional treatment vs. conventional treatment


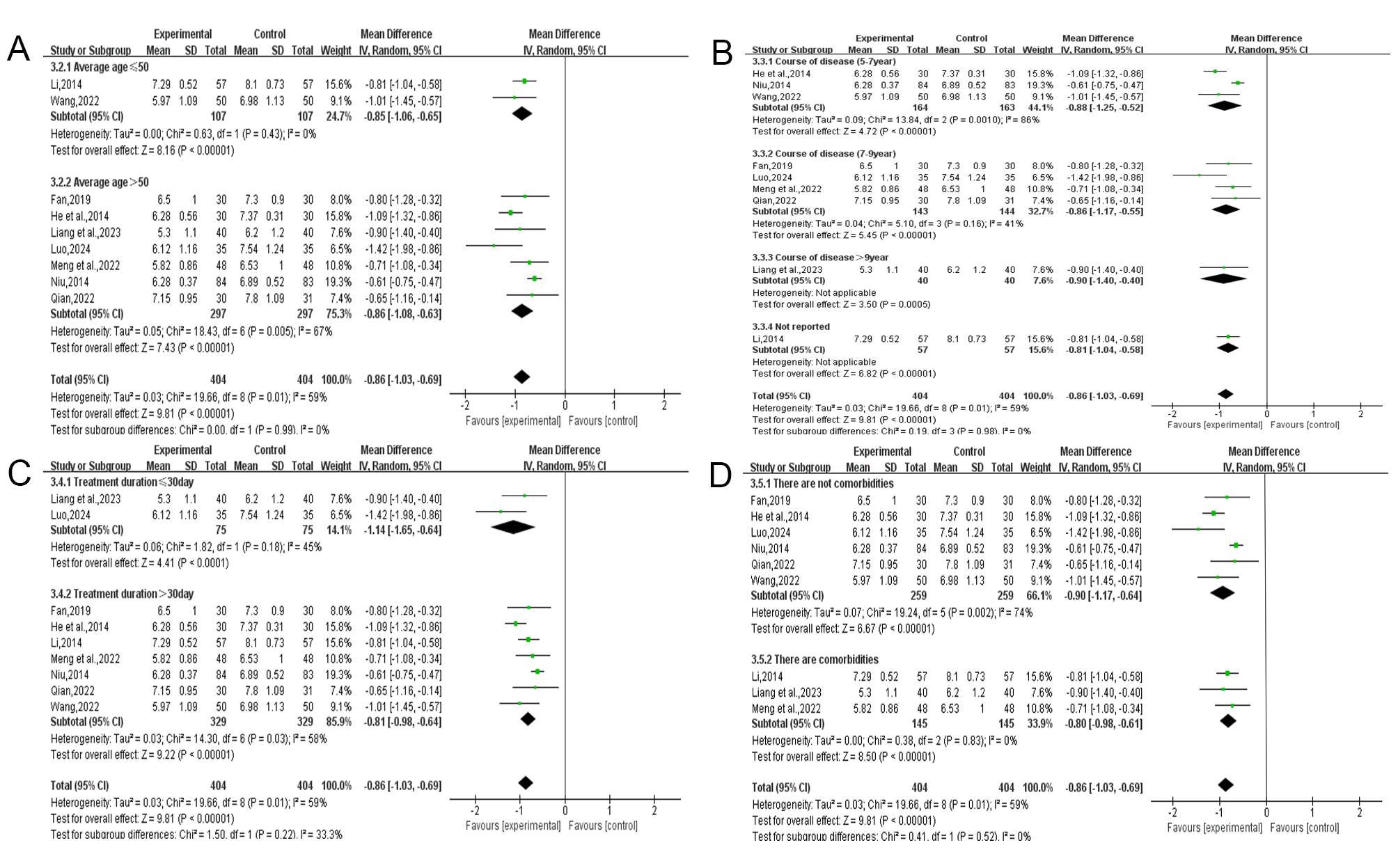


# Supplementary Material S7. Sensitivity analysis


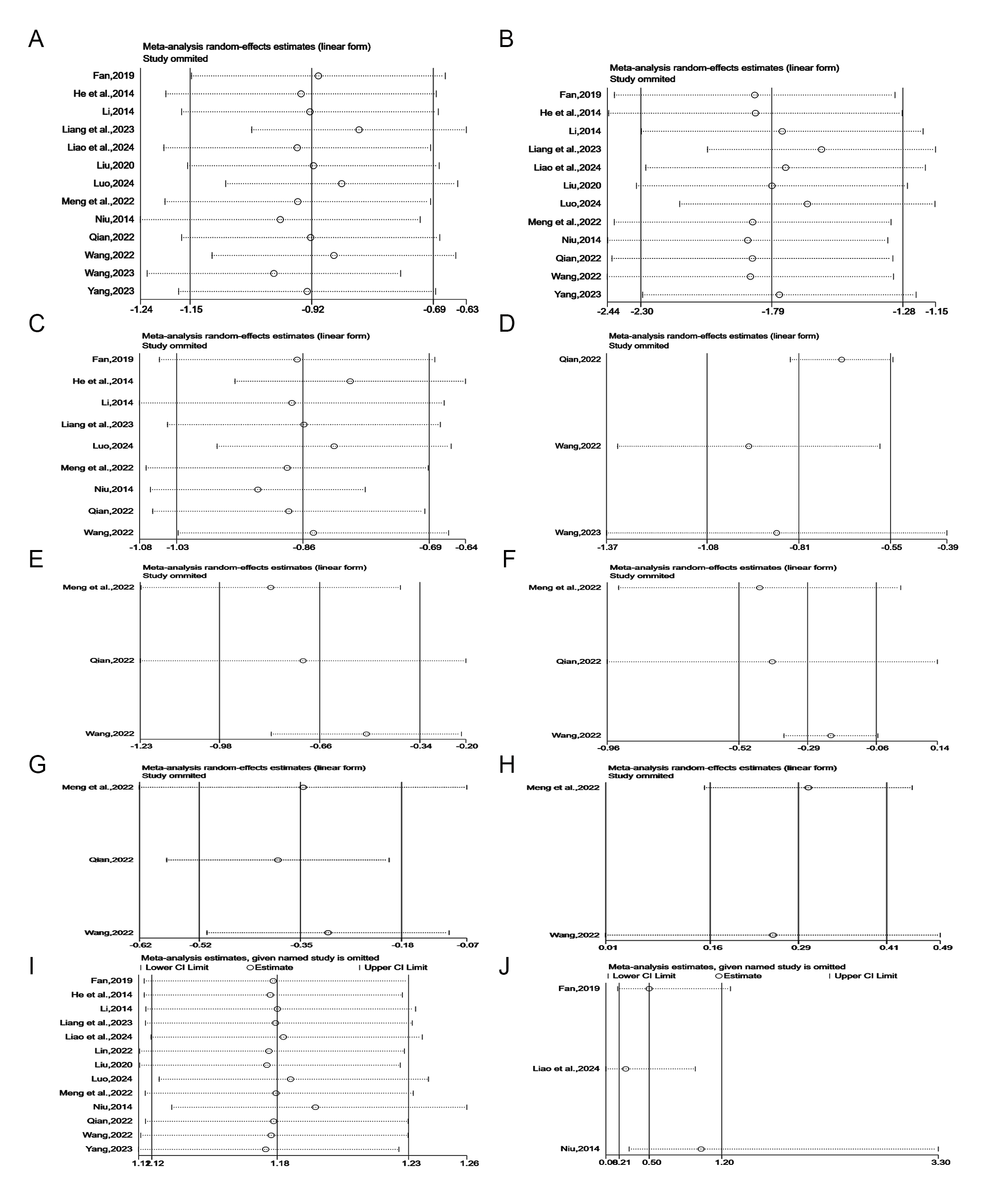


(A) FBG; (B) 2hPG; (C) HbA1c; (D) HOMA-IR; (E) TC; (F) TG; (G) LDL-C; (H) HDL-C; (I) Overall effective rate; (J) Adverse events rate.

# Supplementary Material S8. Labbe plot of overall effective rate


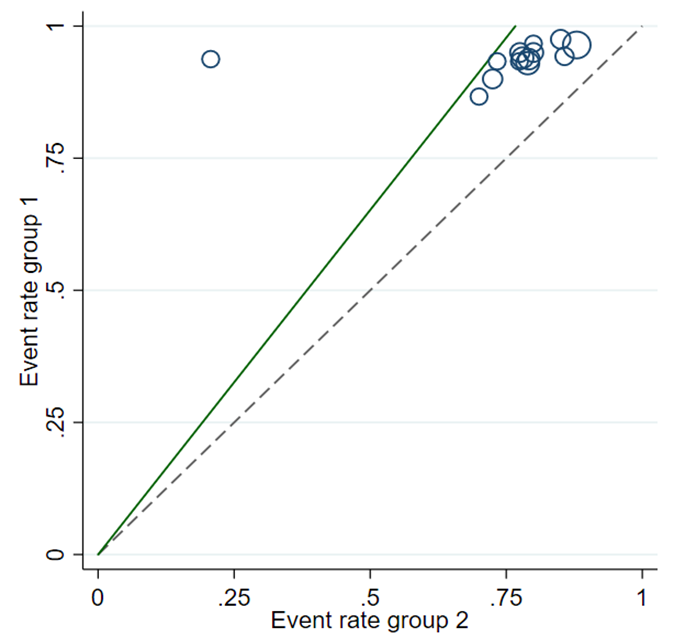


# Supplementary Material S9. Egger’s test of FBG, 2hPG and Overall effective rate


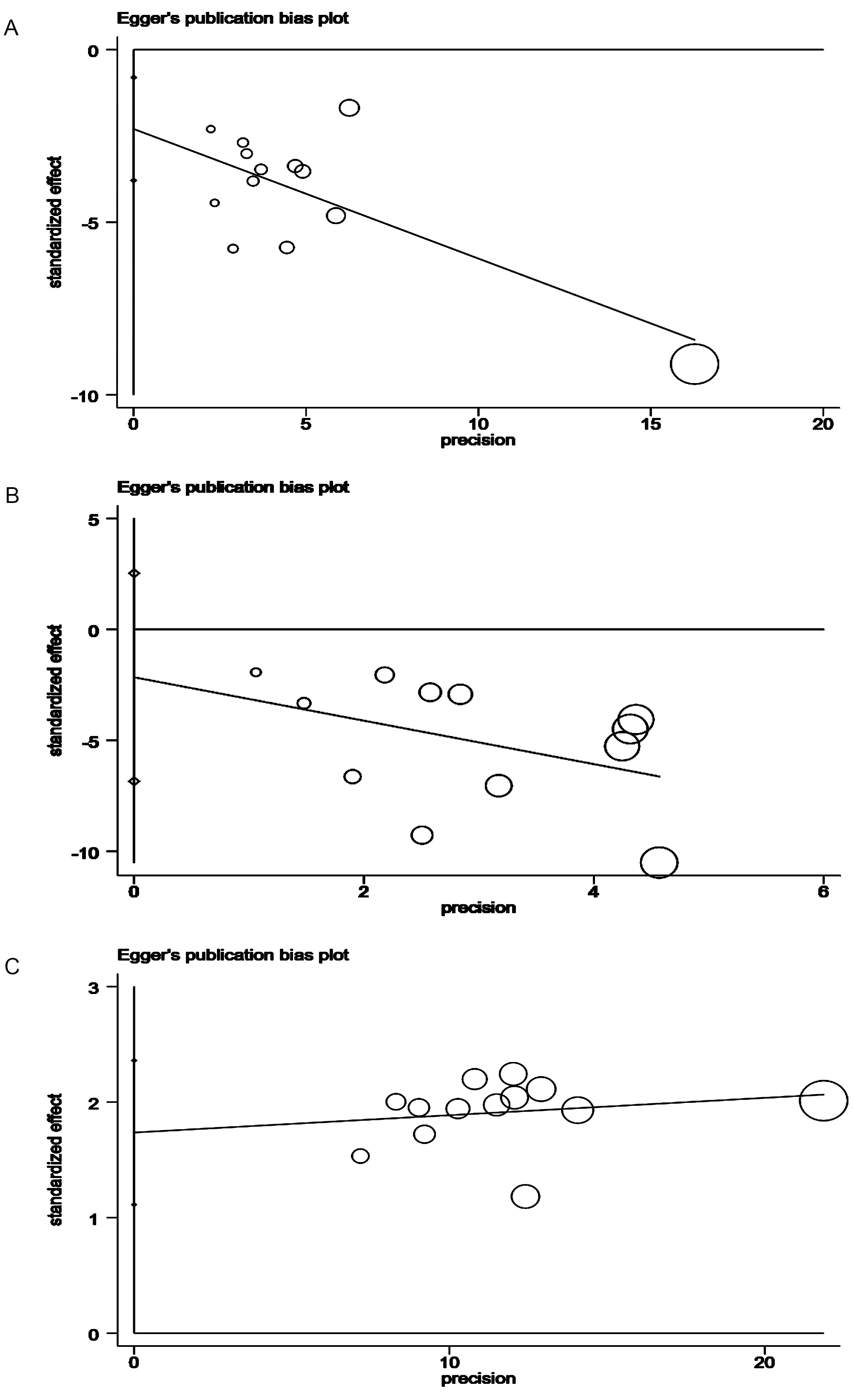


(A) FBG; (B) 2hPG; (C) Overall effective rate.

# Supplementary Material S10. Filled funnel plot


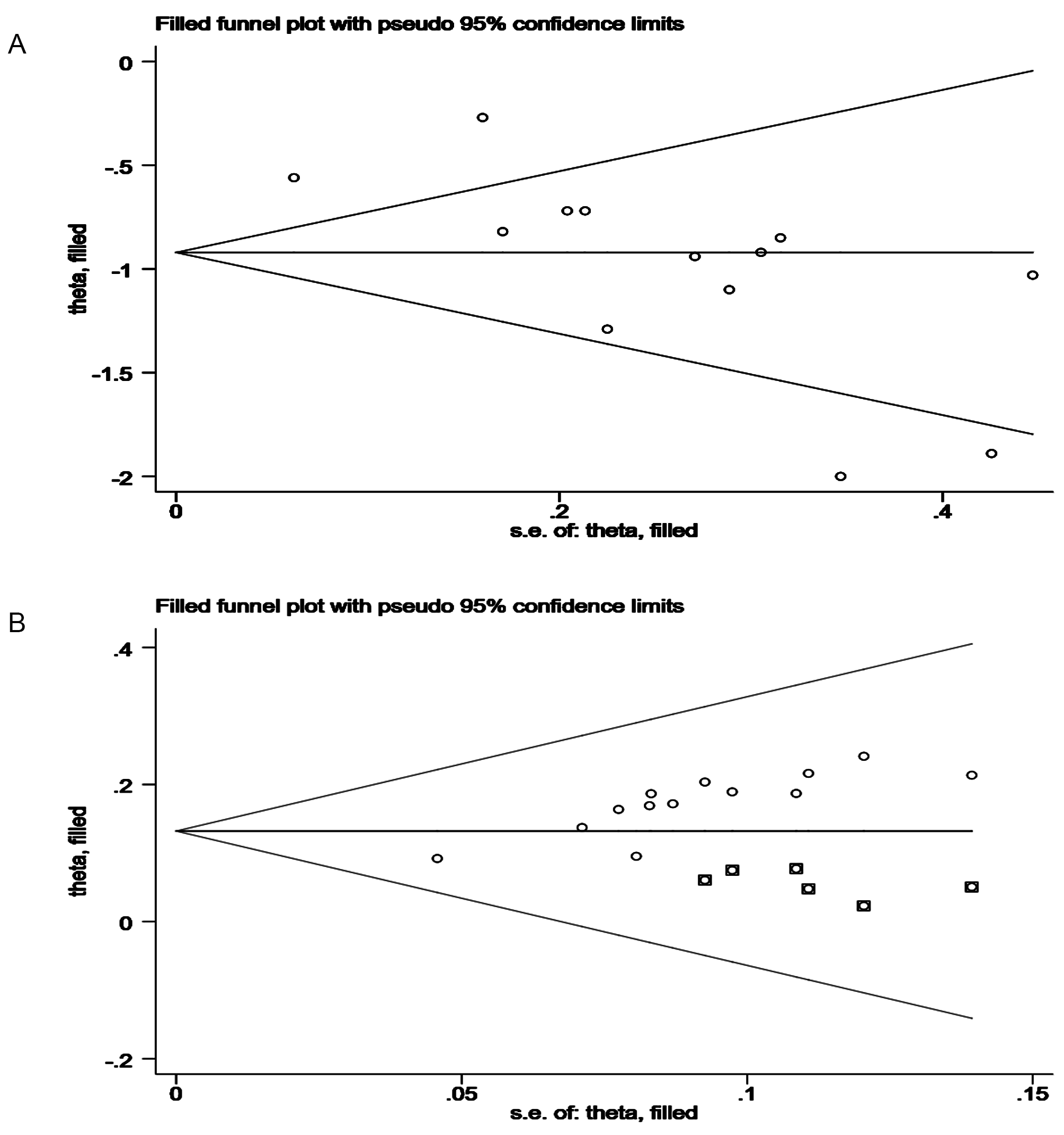


(A) FBG; (B) Overall effective rate.

# Supplementary Material S11. Assessment of evidence quality for each outcome

| **Quality assessment** | | | | | | | | | | | | | | | | | **No of patients** | | | | | | | **Effect** | | | | | | **Quality** | **Importance** |
| --- | --- | --- | --- | --- | --- | --- | --- | --- | --- | --- | --- | --- | --- | --- | --- | --- | --- | --- | --- | --- | --- | --- | --- | --- | --- | --- | --- | --- | --- | --- | --- |
|  |  |  |  |  |  |  |  |  |  |  |  |  |  |  |  |  |  |  |  |  |  |  |  |  |  |  |  |  |  |  |  |
| **No of studies** | **Design** | | | | | **Risk of bias** | **Inconsistency** | | | **Indirectness** | | | | **Imprecision** | | **Other considerations** | **Treatment** | | | **Control** | | | | **Relative (95% CI)** | | **Absolute** | | | |  |  |
| **FBG(mmol/L) (Better indicated by lower values)** | | | | | | | | | | | | | | | | | | | | | | | | | | | | | | | |
| 13 | randomised trials | | | | | serious^1^ | serious^2^ | | | no serious indirectness | | | | no serious imprecision | | reporting bias^4^ | 546 | | | 543 | | | | - | | MD 0.92 lower (1.15 to 0.69 lower) | | | |  VERY LOW | CRITICAL |
| **2hPG(mmol/L) (Better indicated by lower values)** | | | | | | | | | | | | | | | | | | | | | | | | | | | | | | | |
| 12 | randomised trials | | | | | serious^1^ | serious^2^ | | | no serious indirectness | | | | no serious imprecision | | none | 514 | | | 514 | | | | - | | MD 1.79 lower (2.3 to 1.28 lower) | | |  LOW | | CRITICAL |
| **HbA1c(%) (Better indicated by lower values)** | | | | | | | | | | | | | | | | | | | | | | | | | | | | | | | |
| 9 | randomised trials | | | | | serious^1^ | serious^2^ | | | no serious indirectness | | | | no serious imprecision | | none | 404 | | | 404 | | | | - | | MD 0.86 lower (1.03 to 0.69 lower) | | | |  LOW | CRITICAL |
| **HOMA-IR (Better indicated by lower values)** | | | | | | | | | | | | | | | | | | | | | | | | | | | | | | | |
| 3 | randomised trials | | | | | serious^1^ | serious^2^ | | no serious indirectness | | | | | serious^3^ | | none | 112 | | | | 110 | | | - | | MD 0.81 lower (1.08 to 0.55 lower) | | | |  VERY LOW | IMPORTANT |
| **TC(mmol/L) (Better indicated by lower values)** | | | | | | | | | | | | | | | | | | | | | | | | | | | | | | | |
| 3 | randomised trials | | | | serious^1^ | | no serious inconsistency | | no serious indirectness | | | | | serious^3^ | | none | 128 | | | | 129 | | | - | | MD 0.64 lower (0.9 to 0.38 lower) | | |  LOW | | IMPORTANT |
| **TG(mmol/L) (Better indicated by lower values)** | | | | | | | | | | | | | | | | | | | | | | | | | | | | | | | |
| 3 | randomised trials | | | serious^1^ | | | no serious inconsistency | no serious indirectness | | | | | | serious^3^ | | none | | 128 | | | | 129 | | - | | MD 0.25 lower (0.4 to 0.1 lower) | |  LOW | | | IMPORTANT |
| **LDL-C(mmol/L)** **(Better indicated by lower values)** | | | | | | | | | | | | | | | | | | | | | | | | | | | | | | | |
| 3 | randomised trials | | serious^1^ | | | | no serious inconsistency | | no serious indirectness | | | | | serious^3^ | | none | | 128 | | | | | 129 | - | | MD 0.35 lower (0.52 to 0.18 lower) | |  LOW | | | IMPORTANT |
| **HDL-C(mmol/L) (Better indicated by higher values)** | | | | | | | | | | | | | | | | | | | | | | | | | | | | | | | |
| 2 | randomised trials | | serious^1^ | | | | no serious inconsistency | | | | no serious indirectness | | | serious^3^ | none | | | 98 | | | | | 98 | | - | | MD 0.29 higher (0.16 to 0.41 higher) |  LOW | | | IMPORTANT |
| **Overall effective rate (Better indicated by higher values)** | | | | | | | | | | | | | | | | | | | | | | | | | | | | | | | |
| 13 | randomised trials | | serious^1^ | | | | no serious inconsistency | | | | | no serious indirectness | no serious imprecision | | reporting bias^4^ | | | 521/554  (94%) | | | | | 442/554  (79.8%) | | RR 1.18 (1.12 to 1.23) | | 144 more per 1000 (from 96 more to 184 more) |  LOW | | | IMPORTANT |
| **Adverse event rate (Better indicated by lower values)** | | | | | | | | | | | | | | | | | | | | | | | | | | | | | | | |
| 3 | randomised trials | serious^1^ | | | | | no serious inconsistency | | | | | no serious indirectness | | serious^3^ | | none | | | 7/154  (4.5%) | | | | 14/153  (9.2%) | | RR 0.5 (0.21 to 1.2) | | 46 fewer per 1000 (from 72 fewer to 18 more) |  LOW | | | IMPORTANT |

^1^ Poor methodological quality, such as not using blinding or not reporting in detail the specific methods for generating random sequences and allocation concealment.

^2^ Large differences in the effect size of each study point or small overlap of confidence intervals, or large heterogeneity.

^3^ Small sample size or wide confidence interval.

^4^ The funnel plot showed asymmetric distribution and Egger's test indicated possible publication bias.
